# Novel iodoquinazolinones bearing sulfonamide moiety as potential antioxidants and neuroprotectors

**DOI:** 10.1038/s41598-023-42239-2

**Published:** 2023-09-20

**Authors:** Aiten M. Soliman, Walid M. Ghorab, Dina M. Lotfy, Heba M. Karam, Mostafa M. Ghorab, Laila A. Ramadan

**Affiliations:** 1 Drug Chemistry Laboratory, Drug Radiation Research Department, National Center for Radiation Research and Technology (NCRRT), Egyptian Atomic Energy Authority (EAEA), Cairo, 11787 Egypt; 2 Pharmacology and Toxicology Laboratory, Drug Radiation Research Department, National Center for Radiation Research and Technology (NCRRT), Egyptian Atomic Energy Authority (EAEA), Cairo, 11787 Egypt; 3 Department of Pharmacology and Toxicology, Faculty of Pharmacy, Egyptian Russian University, Cairo-Suez Road, Badr City, Cairo 11829 Egypt

**Keywords:** Medicinal chemistry, Organic chemistry, Neurology

## Abstract

In a search for new antioxidants, a set of new iodoquinazolinone derivatives bearing benzenesulfonamide moiety and variable acetamide pharmacophores **5–17** were designed and synthesized. The structures of the synthesized compounds were confirmed based on spectral data. Compounds **5**–**17** were screened using in vitro assay for their antioxidant potential and acetylcholinesterase (AChE) inhibitory activity. The 2-(6-iodo-4-oxo-3-(4-sulfamoylphenyl)-3,4-dihydroquinazolin-2-ylthio)-*N*-(pyrazin-2-yl) acetamide **14** was the most active scaffold with potent AChE inhibitory activity. Compound **14** showed relative safety with a median lethal dose of 300 mg/kg (LD_50_ = 300 mg/kg), in an acute toxicity study. The possible antioxidant and neuroprotective activities of **14** were evaluated in irradiated mice. Compound **14** possessed in vivo AChE inhibitory activity and was able to modify the brain neurotransmitters. It was able to cause mitigation of gamma radiation-induced oxidative stress verified by the decline in Myeloperoxidase (MPO) and increase of glutathione (GSH) levels. Also, **14** restored the alterations in behavioral tests. Molecular docking of **14** was performed inside MPO and AChE active sites and showed the same binding interactions as that of the co-crystallized ligands considering the binding possibilities and energy scores. These findings would support that **14** could be considered a promising antioxidant with a neuromodulatory effect.

## Introduction

Exposure to ionizing radiation is harmful to living organisms as it causes damage to DNA leading to cancer and other diseases^1^. It is important to manage and minimize exposure to ionizing radiation, especially in medical, occupational and environmental surroundings^2,3^. Radiation used in medicine is the largest source of man-made radiation to which people are exposed^4^. It has been well documented that high-dose radiation exposure, such as brain radiotherapy, causes brain damage and cognitive impairment^5,6^. Even sustained exposure to low doses of ionizing radiation, such as repeated X-rays or computed tomography (CT) scans and non-ionizing radiation like mobile devices may also induce significant brain neuropathological changes and subsequent neurological and neuropsychological disorders^7^. Almost all human tissues are sensitive to the carcinogenic effects of radiation^8^. Prenatal exposure to radiation increases the vulnerability of developing fetal brain damage, leading to microcephaly, mental retardation, epilepsy and brain tumors^9^. Postpartum exposure can also have harmful effects on the nervous system, significantly reducing neurogenesis, glial formation and endothelial growth, leading to cognitive impairment and neuroinflammation with aging^10^. With an increasing incidence of head and neck malignancies, radiotherapy plays a key role in the treatment of head and neck cancers, either alone or in combination with surgery, chemotherapy and molecular targeting agents. However, it can cause damage to normal brain tissue, leading to serious health consequences such as neuroinflammation, neuronal loss, impairment of neurogenesis and subsequent cognitive impairment^11^. Hence, there is a need to use radioprotective drugs to protect organs at risk. This is because high-energy radiation is necessary for ion-beam radiotherapy, and the possible risk of high linear energy transfer radiation in the surrounding normal tissue may be of more general concern, even though the absolute dose level is reduced^12,13^. Radiation exposure causes a wide range of damages through the generation of reactive oxygen species (ROS) that lead to increased oxidative stress and altered levels of inflammatory mediators which have been strongly implicated in brain tissue and neuronal damage^14,15^, including loss of neural stem cells and damage to neuronal structures^16^. It can also cause changes in neural and cognitive functions and destruction of the blood brain barrier^17^.

The central nervous system (CNS) is inherently susceptible to oxidative stress and is highly active in oxidative metabolism, resulting in relatively high intracellular production of O_2_^−^ and other ROS^18^. Antioxidant supplementation is one conceivable policy to maintain redox homeostasis by quenching excessive ROS and reinforcing endogenous antioxidative defense systems against oxidative stress^19,20^.

Acetylcholinesterase (AChE) is a hydrolase that catalyzes the fast breakdown of the neurotransmitter acetylcholine (ACh) and so plays an important role in cholinergic transmission^21^. Therefore, studies of antioxidant and neuroprotector agents that might slow the progression of Alzheimer’s disease (AD) by protecting neurons from oxidative stress and acting as cholinesterase inhibitors have gained increasing interest^22^. Myeloperoxidase (MPO) is a hemeprotein member of the peroxidase family that can be found in the azurophil granules of polymorphonuclear neutrophils^23^. This strong oxidant species can cross the cell membrane, promoting not only the chlorination of lipids, nucleic acids and carbohydrates but also the deamination of amino acids^24^, which are strongly associated with chronic degenerative diseases such as cardiovascular disease and atherosclerosis^25^, cancer^26^, Alzheimer’s and Parkinson’s^27^. Indeed, radiation-induced toxicity in normal tissues is undesirable, but a common side effect of radiation exposure and an important limiting factor in radiotherapy^28^. Inflammatory responses to radiation are involved in tissue toxicity and MPO-containing neutrophils and macrophages are the key inflammatory cells recruited to exposed tissues^29^. Consequently, MPO plays a critical role in innate immunity and inflammatory diseases. Thus, MPO is an attractive target in drug design.

**Figure 1:**
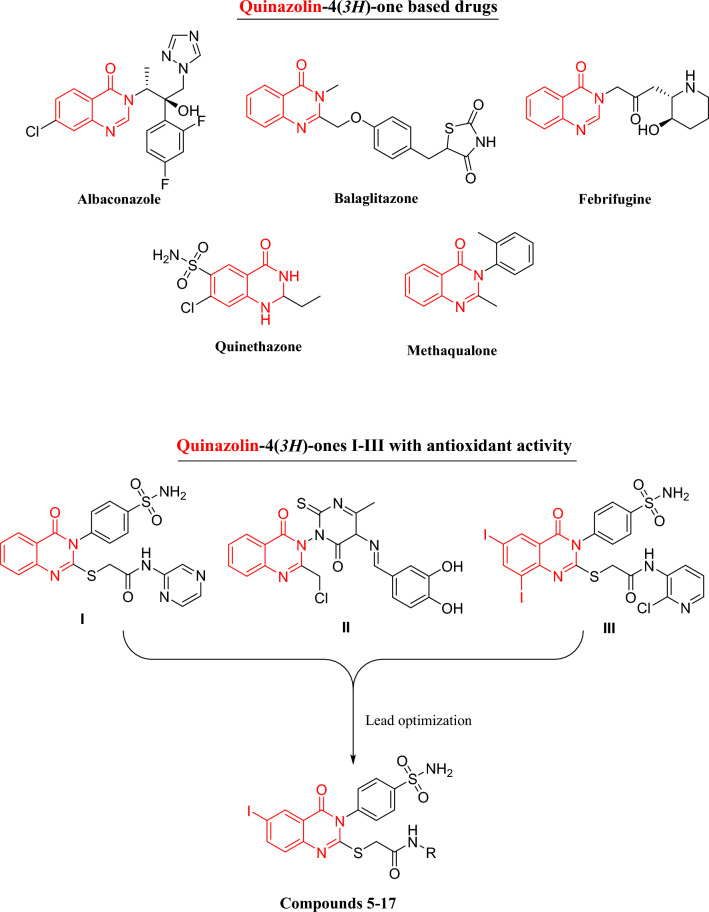
Quinazolinone based drugs and antioxidant agents.

Quinazoline is a fused heterocycle that serves as a crucial scaffold in medicinal chemistry because of its wide range of pharmacological activity both in vitro and in vivo and its ease of synthesis^30–38^. Different medical disorders have been treated clinically with a variety of synthetic and natural quinazoline-based medications. The most known drugs among 4(*3H*)-quinazolinones are the triazole antifungal drug albaconazole, the antihyperglycemic agent balaglitazone, the antimalarial agent febrifugine, the antihypertensive agent quinethazone and the GABAergic quinazolines (e.g., methaqualone) (Fig. 1)^39^. The antioxidant activity was also reported for structurally diverse quinazolines such as 2-((4-oxo-3-(4-sulfamoyl-phenyl)-3,4-dihydro-quinazolin-2-yl)thio)-*N*-(pyrazin-2-yl)-acetamide (**I**)^40^, the 2-(chloromethyl)-3-(4-methyl-6-oxo-5-((*E*)-phenyldiazenyl)-2-thioxo-5,6-dihydropyrimidine-1(2*H*)-yl)quinazoline-4(3*H*)-ones (**II**)^41^, and the iodinated quinazolinone bearing a benzensulfonamide moiety (**III**)^42^ (Fig. 1).

Consequently, we designed and synthesized a series of 2-((6-iodo-4-oxo-3-(4-sulfamoylphenyl)-3,4-dihydroquinazolin-2-yl)thio)-*N*-(substituted) acetamides anticipating this scaffold to exert AChE & MPO inhibition effects, and acting as antioxidant and neuromodulatory agents. The AChE inhibitory activities of all the new compounds were estimated in vitro. The scavenging activities of the synthesized compounds were measured using a DPPH assay, and an acute toxicity study was performed for the most active compound in vivo. The present study was extended to investigate the possible protective effect against whole-body gamma irradiation-induced brain damage and oxidative stress in experimental mice. This was carried out through the evaluation of mice behavior and the subsequent assessment of AChE and norepinephrine (NE) content in brain homogenates. Likewise, the antioxidant activity of compound **14** was measured by estimation of brain MPO and Glutathione (GSH) levels. Molecular docking was performed inside the binding sites of AChE and MPO to gain insights into the molecular interactions and possible modes of action.

## Materials and methods

### Chemistry

Uncorrected melting points were determined using a Gallen Kamp melting point apparatus (Sanyo Gallen Kamp, UK). For thin layer chromatography, precoated silica gel plates (Kieselgel 0.25 mm, 60 F254, Merck, Germany) were utilized. Applying an n-hexane/ethyl acetate (8:2) developing solvent mixture, the spots were identified using ultraviolet light. An FT-IR spectrophotometer (Perkin Elmer, USA) was used to record the IR spectra (KBr disc). On an NMR spectrophotometer (Bruker AXS Inc., Switzerland) operating at 500 and/or 400 MHz for ^1^H and 125.76 MHz for ^13^C, NMR spectra were scanned. Chemical shifts were measured using DMSO-*d*_*6*_ and are given as δ-values (ppm) in relation to an internal standard tetramethyl silane. A model 2400 CHNSO analyzer (Perkin Elmer, USA) was used to perform elemental analysis. Each value measured was within ± 0.4% of the theoretical values. The reagents used were all of AR grade.

#### 4-isothiocyanatobenzenesulfonamide (**2**)^43^

##### 4-(6-iodo-2-mercapto-4-oxoquinazolin-3(*4H*)-yl)benzenesulfonamide (**4**)^44^

*General procedure for the synthesis of 3,4-dihydroquinazolin-sulfonamide derivatives (****5***–***17****):* A mixture of 2-chloro-*N*-substituted acetamide derivatives (0.012 mol), compound **4** (3.33 g, 0.01 mol) and anhydrous K_2_CO_3_ (1.38 g, 0.01 mol) was stirred at room temperature for 12 h in dry acetone (50 mL). The reaction mixture was filtered and the product formed was crystallized from ethanol to give **5–17**.

##### 2-(6-Iodo-4-oxo-3-(4-sulfamoylphenyl)-3,4-dihydroquinazolin-2-ylthio)-*N*-(5-methylisoxazol-3-yl) acetamide (**5**)

**5:** Yield, 88%; m.p. 309.8 °C. IR: 3302, 3211, 3124 (NH_2_, NH), 3057 (arom.), 2991, 2854 (aliph.), 1699, 1662 (CO), 1340, 1190 (SO_2_). ^1^H NMR (DMSO-*d*_*6*_): *δ* 2.34 (s, 3H), 4.12 (s, 2H), 6.55 (s, 1H), 7.29 (d, 1H, *J* = 10 Hz), 7.62 (s, 1H, NH), 7.70 (d, 2H, *J* = 6 Hz, AB), 8.04–8.05 (m, 3H), 8.09 (d, 2H, *J* = 6 Hz*,* AB), 8.34 (d, 1H, *J* = 1.5 Hz). ^13^CNMR: 12.07, 30.60, 90.58, 96.39, 121.73 (2), 127.51 (2), 128.44 (2), 130.57, 135.16, 138.82, 143.73, 145.92, 146.73, 157.25, 159.76, 166.44, 170.05. Anal. Calcd. for C_20_H_16_IN_5_O_5_S_2_ (597.41): C, 40.21; H, 2.70; N, 11.72. Found: C, 40.57; H, 3.02; N, 12.05.

##### 2-(6-Iodo-4-oxo-3-(4-sulfamoylphenyl)-3,4-dihydroquinazolin-2-ylthio)-*N*-(thiazol-2-yl) acetamide (**6**)

**6:** Yield, 89%; m.p. 316.2 °C. IR: 3316, 3225, 3105 (NH_2_, NH), 3078 (arom.), 2963, 2845 (aliph.), 1679, 1662 (CO), 1335, 1167 (SO_2_). ^1^H NMR (DMSO-*d*_*6*_): *δ* 4.11 (s, 2H), 7.16 (d, 1H, *J* = 5 Hz), 7.25 (d, 1H, *J* = 6 Hz), 7.47–7.50 (m, 2H), 7.73 (d, 2H, *J* = 6 Hz, AB), 8.04–8.08 (m, 4H), 8.33 (s, 2H). ^13^CNMR: 30.80, 90.64, 113.83, 121.75 (2), 127.00, 127.52, 128.39 (2), 130.13, 135.16, 138.06, 138.81, 143.72, 145.94, 157.15, 158.24, 159.75, 166.33. Anal. Calcd. for C_19_H_14_IN_5_O_4_S_3_ (599.45): C, 38.07; H, 2.35; N, 11.68. Found: C, 38.39; H, 2.69; N, 12.05.

##### *N*-(6-Ethoxybenzo[d]thiazol-2-yl)-2-(6-iodo-4-oxo-3-(4-sulfamoylphenyl)-3,4-dihydroquinazolin-2-ylthio) acetamide (**7**)

**7:** Yield, 79%; m.p. 257.3 °C. IR: 3345, 3294, 3182 (NH_2_, NH), 3067 (arom.), 2975, 2853 (aliph.), 1669 (CO), 1389, 1158 (SO_2_). ^1^H NMR (DMSO-*d*_*6*_): *δ* 1.87 (t, 3H, *J* = 1.5 Hz), 3.69–3.73 (m, 4H), 6.13 (t, 1H, *J* = 10 Hz), 6.55 (d, 1H, *J* = 5 Hz), 6.68–6.72 (m, 2H), 6.98 (d, 2H, *J* = 9 Hz, AB), 7.36–7.39 (m, 4H), 7.67 (s, 2H), 7.68 (s, 1H). ^13^CNMR: 15.31, 38.76, 64.78, 91.03, 107.11, 114.77, 119.16, 120.31 (2), 122.44, 128.41, 129.32 (2), 131.45, 132.85, 136.13, 134.11, 144.35, 144.65, 147.53, 155.28, 160.62, 160.95, 166.80, 169.87. Anal. Calcd. for C_25_H_20_IN_5_O_5_S_3_ (693.56): C, 43.29; H, 2.91; N, 10.10. Found: C, 43.63; H, 3.24; N, 10.46.

##### 2-(6-Iodo-4-oxo-3-(4-sulfamoylphenyl)-3,4-dihydroquinazolin-2-ylthio)-*N*-(6-nitrobenzo[d]thiazol-2-yl) acetamide (**8**)

**8:** Yield, 71%; m.p 258.1 °C. IR: 3385, 3263, 3176 (NH_2_, NH), 3071 (arom.), 2950, 2857 (aliph.), 1684 (CO), 1328, 1163 (SO_2_). ^1^H NMR (DMSO-*d*_*6*_): *δ* 3.14 (s, 2H), 4.12 (s, 2H), 7.33 (d, 1H, *J* = 10 Hz), 7.62 (d, 1H, *J* = 5 Hz), 7.73 (d, 2H, *J* = 6 Hz, AB) 8.05–8.07 (m, 3H), 8.14–8.15 (m, 2H), 8.32 (d, 1H, *J* = 1.5 Hz), 8.71 (s, 1H). ^13^CNMR: 39.13, 90.33, 118.16, 119.08, 121.41, 121.72 (2), 127.54, 128.55, 130.64 (2), 133.13, 135.10, 139.08, 141.85, 143.65, 145.82, 146.95, 155.64 (2), 158.06, 159.86, 172.60. Anal. Calcd. for C_23_H_15_IN_6_O_6_S_3_ (694.50): C, 39.78; H, 2.18; N, 12.10. Found: C, 39.47; H, 2.01; N, 11.79.

##### 2-(6-Iodo-4-oxo-3-(4-sulfamoylphenyl)-3,4-dihydroquinazolin-2-ylthio)-*N*-morpholinoacetamide (**9**)

**9:** Yield, 68%; m.p. 306.1 °C. IR: 3302, 3284, 3145 (NH_2_, NH), 3083 (arom.), 2976, 2892 (aliph.), 1670, 1649 (CO), 1331, 1158 (SO_2_). ^1^H NMR (DMSO-*d*_*6*_): *δ* 3.04 (s, 2H), 3.38 (t, 4H, *J* = 12 Hz), 3.72 (s, 4H, *J* = 12 Hz), 4.09 (s, 2H), 7.38 (s, 1H, NH) 7.43 (d, 1H, *J* = 6 Hz), 7.74 (d, 2H, *J* = 6 Hz, AB), 8.03 (d, 2H, *J* = 9 Hz*,* AB), 8.14 (d, 1H, *J* = 6 Hz), 8.34 (d, 1H, *J* = 2 Hz). ^13^CNMR: 37.81, 49.28 (2), 66.56 (2), 91.06, 115.88 (2), 120.73, 121.89, 127.52 (2), 130.75, 135.16, 138.89, 143.86, 145.99, 157.62, 159.82, 165.19. Anal. Calcd. for C_20_H_20_IN_5_O_5_S_2_ (601.44): C, 39.94; H, 3.35; N, 11.64. Found: C, 39.60; H, 3.02; N, 11.27.

##### Ethyl 4-(2-(6-iodo-4-oxo-3-(4-sulfamoylphenyl)-3,4-dihydroquinazolin-2-ylthio) acetamido) piperidine-1-carboxylate (**10**)

**10:** Yield, 84%; m.p. 215.7 °C. IR: 3354, 3285, 3131 (NH_2_, NH), 3083 (arom.), 2972, 2878 (aliph.), 1686 (CO), 1334, 1159 (SO_2_). ^1^HNMR (DMSO-*d*_*6*_): *δ* 1.18 (t, 3H, *J* = 7 Hz), 1.69–1.72 (m, 4H), 2.90–2.95 (m, 4H), 3.71–3.88 (m, 3H), 4.05 (q, 2H, CH_2_), 7.36 (d, 1H, *J* = 8 Hz), 7.58 (s, 1H, NH), 7.71 (d, 2H, *J* = 8 Hz, AB), 8.01 (d, 2H, *J* = 8 Hz, AB), 8.15 (d, 1H, *J* = 8 Hz), 8.25 (d, 1H, *J* = 7 Hz), 8.34 (s, 2H, NH_2_). ^13^CNMR: 13.56, 30.60, 35.72 (2), 41.77 (2), 46.84, 60.42, 89.32, 120.92 (2), 126.75 (2), 127.63 (2), 129.74, 134.53, 138.15, 142.97, 145.15, 154.55, 156.71, 159.18, 166.07. Anal. Calcd. for C_24_H_26_IN_5_O_6_S_2_ (671.53): C, 42.93; H, 3.90; N, 10.43. Found: C, 42.58; H, 3.59; N, 10.11.

##### *N*-(1-Benzylpiperidin-4-yl)-2-(6-iodo-4-oxo-3-(4-sulfamoylphenyl)-3,4-dihydroquinazolin-2-ylthio) acetamide (**11**)

**11:** Yield, 80%; m.p. 266.7 °C. IR: 3307, 3317, 3219 (NH_2_, NH), 3066 (arom.), 2942, 2845 (aliph.), 1690 (CO), 1394, 1155 (SO_2_). ^1^H NMR (DMSO-*d*_*6*_): *δ* 1.87–1.90 (m, 4H), 2.50–2.97 (m, 4H), 3.71 (p, 1H), 3.88 (s, 2H), 4.22 (s, 2H), 7.37–7.59 (m, 6H), 7.70 (d, 2H, *J* = 8 Hz, AB), 7.92–7.94 (m, 2H), 8.01 (d, 2H, *J* = 8 Hz, AB), 8.15 (s, 2H, NH_2_), 8.51 (d, 1H, *J* = 1.5 Hz). ^13^CNMR: 31.19 (3), 40.31, 51.74 (2), 60.86, 91.07, 118.48 (2), 118.70, 121.84, 127.02, 128.68 (2), 129.10 (2), 130.26 (2), 135.13, 135.66, 138.88, 139.53, 143.88, 144.22, 157.61, 159.06, 176.80. Anal. Calcd. for C_28_H_28_IN_5_O_4_S_2_ (689.59): C, 48.77; H, 4.09; N, 10.16. Found: C, 49.06; H, 4.42; N, 10.45.

##### Ethyl 2-(2-(6-iodo-4-oxo-3-(4-sulfamoylphenyl)-3,4-dihydroquinazolin-2-ylthio) acetamido)benzoate (**12**)

**12:** Yield, 67%; m.p. 262.8 °C. IR: 3302, 3298, 3211 (NH_2_, NH), 3082 (arom.), 2976, 2845 (aliph.), br. 1669 (CO), 1396, 1156 (SO_2_). ^1^H NMR (DMSO-*d*_*6*_): *δ* 1.25 (t, 3H, *J* = 8 Hz), 4.09 (s, 2H, CH_2_), 4.30 (q, 2H, CH_2_O), 7.16 (t, 1H, *J* = 8 Hz), 7.31 (d, 1H, *J* = 8 Hz), 7.61 (t, 1H, *J* = 7 Hz), 7.68 (d, 2H, *J* = 8 Hz, AB), 7.91 (dd, 1H, *J* = 8 & 1.5 Hz), 7.98 (d, 2H, *J* = 8 Hz, AB), 8.07 (d, 1H, *J* = 1.5 Hz), 8.09 (d, 1H, *J* = 1.5 Hz), 8.32 (d, 1H, *J* = 1.5 Hz), 8.37 (s, 1H, NH), 8.39 (s, 2H, NH_2_). ^13^CNMR: 17.18, 37.81, 55.96, 89.39, 115.70, 120.01, 120.94 (2), 122.64, 125.84, 126.25, 127.72 (2), 128.91, 130.16, 133.63, 134.42, 139.58, 142.83, 146.10, 156.31, 158.26, 159.32, 166.05, 167.44. Anal. Calcd. for C_25_H_21_IN_4_O_6_S_2_ (664.49): C, 45.19; H, 3.19; N, 8.43. Found: C, 45.47; H, 3.54; N, 8.78.

##### Ethyl 3-(2-(6-iodo-4-oxo-3-(4-sulfamoylphenyl)-3,4-dihydroquinazolin-2-ylthio) acetamido)benzoate (**13**)

**13:** Yield, 72%; m.p. 274.6 °C. IR: 3311, 3245, 3170 (NH_2_, NH), 3100 (arom.), 2977, 2862 (aliph.), br. 1693 (CO), 1374, 1156 (SO_2_). ^1^H NMR (DMSO-*d*_*6*_): *δ* 1.32 (t, 3H, *J* = 5 Hz), 4.14 (s, 2H), 4.31 (q, 2H, *J* = 5 Hz), 7.34 (s, 1H, NH), 7.46 (d, 1H, *J* = 6.5 Hz), 7.48 (s, 2H), 7.62 (d, 1H, *J* = 5 Hz), 7.66 (t, 1H, *J* = 5 Hz), 7.76 (d, 2H, *J* = 5.5 Hz, AB), 7.82 (d, 1H, *J* = 10 Hz), 7.84 (d, 1H, *J* = 6.5 Hz), 8.04 (d, 2H, *J* = 5.5 Hz, AB), 8.28 (d, 1H, *J* = 2 Hz), 8.34 (d, 1H, *J* = 1.5 Hz). ^13^CNMR: 14.64, 37.76, 61.32, 91.10, 119.99, 121.90 (2), 123.97, 124.55, 127.53, 128.56, 129.80, 130.76 (2), 130.91, 135.17, 138.87, 139.71, 143.85, 146.02, 146.83, 157.54, 159.80, 165.97, 166.36. Anal. Calcd. for C_25_H_21_IN_4_O_6_S_2_ (664.49): C, 45.19; H, 3.19; N, 8.43. Found: C, 45.56; H, 3.57; N, 8.79.

##### 2-(6-Iodo-4-oxo-3-(4-sulfamoylphenyl)-3,4-dihydroquinazolin-2-ylthio)-*N*-(pyrazin-2-yl) acetamide (**14**)

**14:** Yield, 89%; m.p. 207.7 °C. IR: 3364, 3287, 3149 (NH_2_, NH), 3088 (arom.), 2982, 2853 (aliph.), 1682 (CO), 1304, 1159 (SO_2_). ^1^H NMR (DMSO-*d*_*6*_): *δ* 4.22 (s, 2H), 7.29 (d, 1H, *J* = 6.5 Hz), 7.62 (s, 2H), 7.76 (d, 2H, *J* = 5 Hz, AB), 8.04 (d, 2H, *J* = 5 Hz, AB), 8.11 (d, 1H, *J* = 6 Hz), 8.32 (d, 1H, *J* = 6.5 Hz), 8.39 (s, 1H, *J* = 6.5 Hz), 8.45 (s, 1H), 9.26 (s, 1H), 11.20 (s, 1H). ^13^CNMR: 37.25, 91.11, 121.87 (2), 127.54, 128.54, 130.74 (2), 135.15, 136.59 (2), 138.87 (2), 140.41, 143.24, 146.75, 149.02, 157.43, 159.79, 167.41. Anal. Calcd. for C_20_H_15_IN_6_O_4_S_2_ (594.41): C, 40.41; H, 2.54; N, 14.14. Found: C, 40.78; H, 2.92; N, 14.46.

##### *N*-(2,4-Dioxo-1,2,3,4-tetrahydropyrimidin-5-yl)-2-(6-iodo-4-oxo-3-(4-sulfamoylphenyl)-3, 4-dihydroquinazolin-2-ylthio) acetamide (**15**)

**15:** Yield, 66%; m.p. 281.9 °C. IR: 3359, 3254, 3161 (NH_2_, NH), 3074 (arom.), 2972, 2911 (aliph.), 1698, 1662 (CO), 1341, 1157 (SO_2_). ^1^H NMR (DMSO-*d*_*6*_): *δ* 4.06 (s, 2H), 4.10–4.13 (m, 2H), 7.48 (d, 1H, *J* = 5 Hz), 7.69 (d, 2H, *J* = 10 Hz, AB), 7.71 (s, 1H), 8.03 (d, 2H, *J* = 10 Hz, AB), 8.09 (s, 2H), 8.11 (d, 1H, *J* = 5 Hz), 8.33 (s, 1H), 9.65 (s, 1H). ^13^CNMR: 30.20, 90.73, 113.19, 121.73 (2), 127.51, 128.87, 129.01 (2), 130.58, 135.04, 138.80, 143.60, 145.91, 146.73, 149.93, 157.38, 159.77, 165.31, 166.42. Anal. Calcd. for C_20_H_15_IN_6_O_6_S_2_ (626.40): C, 38.35; H, 2.41; N, 13.42. Found: C, 38.69; H, 2.74; N, 13.79.

##### *N*-(1,3-Dimethyl-2,6-dioxo-1,2,3,6-tetrahydropyrimidin-4-yl)-2-(6-iodo-4-oxo-3-(4-sulfamoylphenyl)-3, 4-dihydroquinazolin-2-ylthio) acetamide (**16**)

**16:** Yield, 91%; m.p. 310.1 °C. IR: 3389, 3275, 3206 (NH_2_, NH), 3072 (arom.), 2984, 2834 (aliph.), 1713, 1649 (CO), 1324, 1150 (SO_2_). ^1^H NMR (DMSO-*d*_*6*_): *δ* 3.13 (s, 3H), 3.23 (s, 3H), 4.01 (s, 2H), 4.35 (d, 1H, *J* = 5 Hz), 7.35 (d, 1H, *J* = 5 Hz), 7.71 (d, 2H, *J* = 8.5 Hz, AB), 8.02 (d, 2H, *J* = 8.5 Hz, AB), 8.09–8.10 (m, 3H), 8.30 (d, 1H, *J* = 5 Hz), 10.18 (s, 1H). ^13^CNMR: 27.82, 29.34, 29.36, 90.51, 91.10, 121.72 (2), 127.46 (2), 128.80 (2), 130.73, 135.01, 139.33, 143.67, 145.72, 147.04, 151.12, 157.99, 159.91, 162.74, 162.78. Anal. Calcd. for C_22_H_19_IN_6_O_6_S_2_ (654.46): C, 40.37; H, 2.93; N, 12.84. Found: C, 40.69; H, 3.23; N, 13.07.

##### 2-(6-Iodo 4-oxo-3-(4-sulfamoylphenyl)-3, 4-dihydroquinazolin-2-ylthio)-*N*-(2-methyl-1, 3-dioxoisoindolin-5-yl) acetamide (**17**)

**17:** Yield, 75%; m.p. 238.4 °C. IR: 3384, 3264, 3163 (NH_2_, NH), 3100 (arom.), 2985, 2873 (aliph.), 1766, 1694 (CO), 1382, 1160 (SO_2_). ^1^H NMR (DMSO-*d*_*6*_): *δ* 3.01 (s, 3H), 4.17 (s, 2H), 7.30 (d, 1H, *J* = 6 Hz), 7.63 (s, 2H), 7.76 (d, 2H, *J* = 5 Hz, AB), 7.82 (d, 1H, *J* = 6 Hz), 7.87–7.88 (m, 2H), 8.05 (d, 2H, *J* = 5 Hz, AB), 8.10 (d, 1H, *J* = 10 Hz), 8.16 (d, 1H, *J* = 2 Hz), 8.32 (d, 1H, *J* = 1.5 Hz). ^13^CNMR: 23.70, 31.23, 91.13, 113.10, 121.89 (2), 123.55, 124.67, 126.37, 127.55, 128.51, 130.75 (2), 133.87, 135.17, 138.85 (2), 143.87, 144.63, 146.77, 157.44, 159.78, 167.12, 168.03, 168.21. Anal. Calcd. for C_25_H_18_IN_5_O_6_S_2_ (675.47): C, 44.45; H, 2.69; N, 10.37. Found: C, 44.14; H, 2.31; N, 10.04.

### Biological evaluation

#### In vitro evaluation

##### Acetylcholine esterase (AChE) inhibitory activity

The cholinesterase enzymes' inhibitory activity was evaluated using modified Osman et al*.*^45^ method. Briefly, samples were initially dissolved in DMSO to achieve a concentration of 10 mg/mL. Then 0.1 mL of the sample was diluted to 1 mL methanol to reach the concentration of 1 mg/mL (concentration 1). From concentration (1), 0.1 mL was further diluted to 1 mL to reach the concentration of 0.1 mg/mL (concentration 2). Concentration 1 (1 mg/mL) will become 0.1 mg/mL inside the plate and concentration 2 will become 0.01 mg/mL due to the addition of the reagents of the assay that dilute the sample ten folds. Samples that achieved inhibition above 50% were further analyzed to determine their IC_50_ values. Donepezil and Physostigmine were used as a reference standard, 10 μL of the indicator solution (0.4 mM in buffer (1): 100 mM tris buffer PH = 7.5 was transferred to a 96-well plate followed by 20 μL of enzyme solution (acetylcholine esterase 0.02 U/mL final concentration in buffer (2): 50 mM tris buffer PH = 7.5 containing 0.1% bovine serum albumin). Then 20 μL of the sample/standard solution was added followed by 140 μL of buffer (1). The mixture was allowed to stand for 15 min at room temperature. Afterward, 10 μL of the substrate (0.4 mM acetylcholine iodide buffer (1) was added immediately to all wells. The plate was incubated in a dark chamber for 20 min at room temperature. At the end of the incubation period, the color was measured at 412 nm. Data are represented as means ± SD. The results were recorded using a microplate reader FluoStar Omega. Each test was conducted in triplicate. The Absorbances of the test samples were corrected by subtracting the absorbance of their respective blank (methanol/ethyl acetate in 50 mmol/L Tris–HCl, (pH 8).

The percentage inhibition was calculated using the equation: Inhibition (%) = 1 – (A sample/A control) × 100 where A sample is the absorbance of the sample extracts and A control is the absorbance of the blank. Extract concentration providing 50% inhibition (IC_50_) was obtained by plotting the percentage inhibition against extract concentration.

##### Free radical scavenging activity

The antioxidant activities of all newly synthesized compounds were measured as radical scavenging activities using 1,1-diphenyl-2-picrylhydrazyl (DPPH) (Sigma Chemical Co., Steinheim, Germany) and compared with ascorbic acid as a reference standard (Sigma-Aldrich Chemie GmbH, Taufkirchen, Germany)^46^.

Where, 1 mL of the test compounds or ascorbic acid at concentrations (12.5, 25, 50 and 100 mg/mL) were mixed with 1 ml of the DPPH solution. The mixtures were incubated for 30 min at room temperature in dark. The absorbance was read using a JENWAY 6315 spectrophotometer (Keison Products, Chelmsford, England) at 517 nm against blank.

It was carried out in triplicate, and average values were used to determine the inhibitory percentage of DPPH according to the following equation: percent inhibition = [(B − A)/B]*100, where A is the absorbance of the compound or standard and B is the absorbance of blank. IC_50_ (µM) of each compound was calculated according to the nonlinear regression dose response-inhibition curve using Prism 5.03 (GraphPad, San Diego, CA, USA) and expressed as mean ± standard error (S.E).

#### In vivo evaluation

##### Animals

Swiss albino male mice (20–25 g) were obtained from the breeding unit of the National Center for Radiation Research and Technology (NCRRT), Cairo, Egypt. Animals were retained in polypropylene cages under well-preserved laboratory conditions (25 ± 5 °C, 40–60% humidity), with alternating cycles of 12 h light/dark. They were fed a standard mouse pellet diet, had water ad libitum, and were housed for acclimatization to the lab environment for 1 week before the experimental study. Mice were treated gently; squeezing, pressure and tough handling were avoided, this was in accordance with ARRIVE guidelines.

The experimental protocol received ethical approval from the Animal Care Committee of the National Centre for Radiation Research and Technology (NCRRT), Cairo, Egypt (Permit Number: 34 A/22), which complies with the guidelines of the US National Institutes of Health for the appropriate care and use of laboratory animals (NIH Publication No. 85-23, revised 2011).

##### Irradiation processes

Mice were exposed to gamma radiation as a single dose of 5 Gy using a Canadian Gamma Cell-40 biological irradiator (^137^Cs) located at the NCRRT, Cairo, Egypt. The dose rate was 0.622 rad/s.

##### Acute toxicity study

Acute toxicity of the most active acetylcholinesterase inhibitor was evaluated through the determination of median lethal dose (LD_50_) which was determined according to Chinedu et al.^47^.

##### Experimental design

Twenty-one mice were blindly allocated into three groups (n = 7). The first group (control) was injected i.p. with 10% DMSO, daily, for 7 days. The second one (irradiated) was treated as a control, and after 1 h from the last DMSO injection, the mice were exposed to 5 Gy gamma radiation^48^. A third group (Compound **14** + irradiation) received 30 mg/kg/day i.p. (1/10 LD_50_) of compound **14**, daily for 7 days. Then on the last day, after 1 h of injection, animals were irradiated at a dose of 5 Gy. After 24 h. of the last injection, behavioral tests were carried out. Immediately after behavioral test, mice were anesthetized using urethane (1.2 mg/kg i.p.)^49^ and sacrificed by cervical dislocation then brain tissues were dissected out, rinsed with ice-cold saline, and dried on a filter paper. Then it was homogenized in ice-cold 0.1 M phosphate buffer saline (pH = 7.4) and stored at – 80 °C for subsequent biochemical analysis.

##### Behavioral tests

All behavioral tests have been carried out under blind conditions to avoid any bias in evaluating the animal's behavior. Mice were allowed to a pre-test session a day before scarification for habituation.

##### Open field test (OFT)

This evaluates locomotors and exploratory activities in mice where, the apparatus is transparent walls and a white floor 30 × 30 × 15 cm, divided into 16 squares of equal area. Each mouse was placed in the center of the open field test apparatus and allowed to roam freely for 5 min and latency time (ie, time taken to start moving) was detected as well as locomotion frequency as ambulation (ie, number of squares crossed), rearing frequency (ie, number of times the mice stand on its hind legs or with its forearm against the wall or in the free air)^50^.

##### Forced swimming test

Mice were independently forced to swim in an open cylindrical container (22 cm diameter, 40 cm height) that contained water (23–25 °C). Each mouse was observed for 5 min to record the immobility time^51^.

##### Biochemical parameters investigated in brain tissue homogenate

Brain homogenates were used for measuring AChE using Mouse Acetylcholinesterase (AChE) ELISA Kit content using the Mouse Acetylcholinesterase ELISA Kit from MyBioSource Cat.No. MBS260553 (San Diego, CA., USA) according to the manufacturer’s instructions. The generation of norepinephrine (NE) in brain tissues was measured using an Eliza kit (MBS2600834) from MyBioSource (San Diego, CA, USA) according to the manufacturer’s instructions.

The antioxidant activity of compound **14** was measured by estimation of MPO activity using an ELISA Kit from MyBioSource Cat.No.: CSB-E08723m (Houston, TX., USA) as well as the level of GSH was evaluated using a colorimetric kit according to the method of E. Beutler^52^.

##### Statistical analysis

Prism 5.0 (GraphPad, San Diego, CA, USA) was used for analyzing the data, and it was expressed as means ± standard error. Comparisons between groups were analyzed by one-way analysis of variance (ANOVA) followed by Bonferroni’s multiple comparison test. IC_50_ values were calculated by the nonlinear regression dose response-inhibition curve.

### Molecular docking

The Molecular Operating Environment (MOE, 10.2008) software was employed to conduct molecular docking study^53,54^. The X-ray structure of the native ligand 2-((3,5-bis(trifluoromethyl)benzyl)amino)-*N*-hydroxy-4-oxo-1,4-dihydropyrimidine-5-carboxamide complexed to human MPO (PDB 4C1M)^55^ and the co-crystallized ligand (1R)-1,6-dimethyl-1,2-dihydronaphtho[1,2-*g*][1]benzofuran-10,11-dione (Dihydrotanshinone I) complexed to AChE (PDB: 4M0E)^56^ were obtained from the protein data bank and utilized as the target receptors. Water molecules were removed from the protein and protonation using the *3D Protonate* protocol was performed to get the receptors prepared for the docking simulation. Using the Triangle Matcher placement and London dG scoring, the co-crystallized ligands were employed to determine the binding location and score. The MMFF94X force field was used to minimize energy with an RMSD grade of 0.01 kcal/mol/Å, taking partial charges into account. The native ligands were re-docked into the active sites of both enzymes to validate the docking technique. The validation showed that the docking approach is appropriate because it accurately recreated the docked pose of the co-crystallized ligands and their binding mode in the active site.

## Results and discussion

### Chemistry

**Scheme 1:**
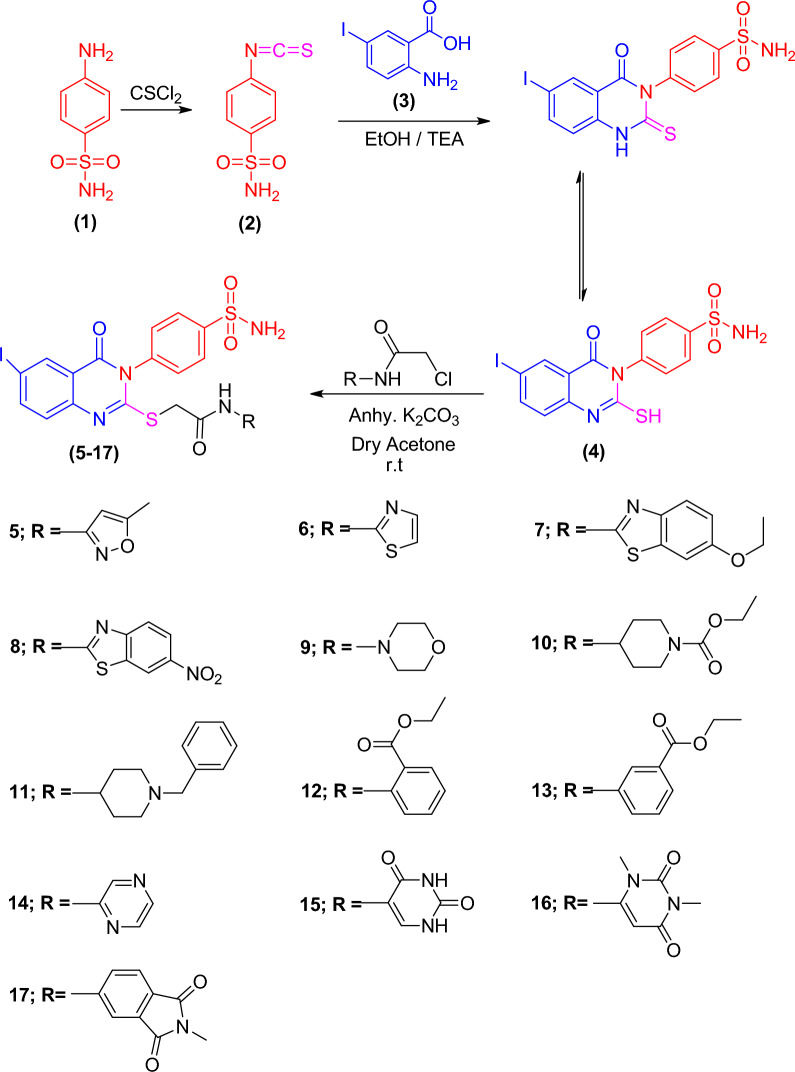
The synthetic pathway for the formation of iodoquinazolinone derivatives** 5**–**17**.

**Figure 2:**
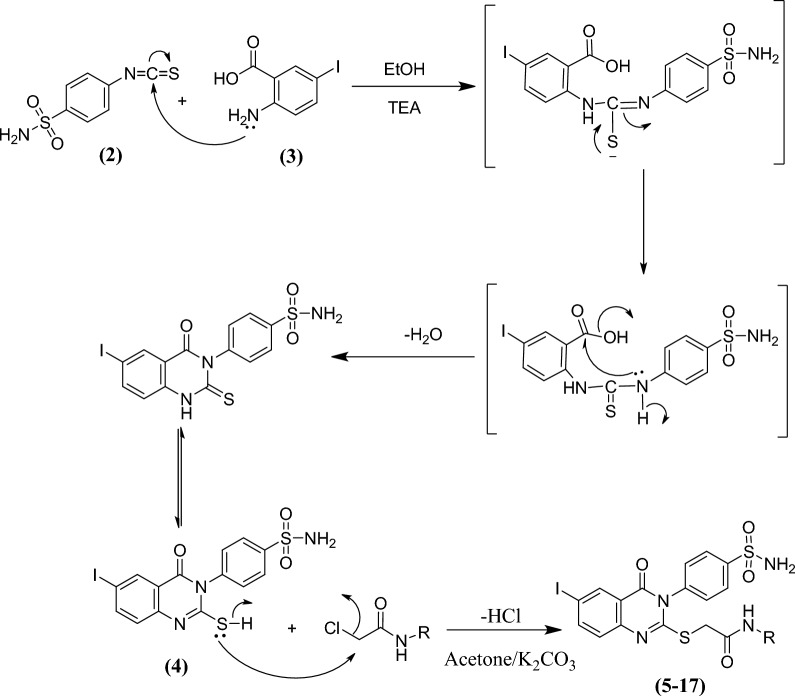
Mechanism of formation of the quinazolinone derivatives **5–17**.

The synthesis of the quinazolinone derivatives **5**–**17**, featuring a biologically active benzenesulfonamide moiety is illustrated in Scheme 1. The starting material 4-(6-iodo-2-mercapto-4-oxoquinazolin-3(*4H*)-yl)benzenesulfonamide **4**^44^ was synthesized in a quantitative yield by cyclo-condensing 4-isothiocyanatobenzenesulfonamide **2**^57^ with 2-amino-5-iodobenzoic acid **3** in refluxing ethanol containing a few drops of triethylamine (TEA). The corresponding iodoquinazolinone derivatives **5**–**17** were obtained by reaction of **4** with 2-chloro-*N*-arylacetamide derivatives in dry acetone containing anhydrous K_2_CO_3_ (1.38 g) (Fig. 2). Based on elemental analysis, IR, ^1^H-NMR and ^13^C-NMR, the structures of all the synthesized compounds were established. IR spectra of **5**–**17** revealed an additional acetamide group showing NH and CO bands at their designated regions. Three singlet signals were detected in ^1^H-NMR spectra of **5–17**, one signal in the range of 3.62–4.17 ppm referring to the SCH_2_, the second at 7.35–8.71 ppm attributed to the NH proton and the third at 7.39–8.09 ppm for SO_2_NH_2_ protons, and aromatic protons displayed in the range of 6.13–8.34 ppm. ^13^C-NMR of **5–17** displayed three characteristic signals peculiar to the SCH_2_ and 2CO carbons.

### Biological activity

#### In vitro screening

##### AChE inhibition activity

Acetylcholinesterase (AChE) is an enzyme that plays a crucial role in cholinergic transmission by catalyzing the rapid hydrolysis of the neurotransmitter acetylcholine^21,58^. The application of acetylcholinesterase inhibitors leads to various effects, which can alleviate the symptoms of Alzheimer's disease^59^. Compounds **5**–**17** were evaluated in vitro for their inhibitory activities against AChE. The ability of the new compounds to inhibit AChE is shown in Table 1. Results indicated that the pyrazine derivative **14** displayed the most potent AChE inhibitory activity as indicated by its lowest IC_50_ value of 11.57 ± 0.45 nM compared to the other compounds in this series, followed by the 1-benzyl piperidine derivative **11** (the second most active compound) with IC_50_ of 23.89 ± 1.41 nM. Donepezil and Physostigmine were used as reference standard drugs, which revealed IC_50_ of 16.40 ± 1.03 nM and 40.01 ± 2.01 nM, respectively. These findings were matched with other studies stating that pyrazine derivatives can act as Acetylcholinesterase inhibitors^60,61^. Besides, compounds **7, 12, 13, 16** and **17** showed adequate inhibitory activities toward AChE with IC_50_ values of 90.02 ± 5.83, 60.65 ± 2.80, 74.83 ± 3.06, 86.17 ± 2.14 and 77.43 ± 1.29 nM, respectively. Meanwhile, the remaining compounds in this series; **5, 6, 8, 9, 10** and **15** have low AChE inhibitory activity (IC_50_ > 100).

Therefore, compound **14** was further chosen as the most active compound to be tested for its antioxidant activity and in vivo evaluations as an antioxidant and neuroprotector. Recent studies confirmed that as Alzheimer’s (AD) progresses, the activity of AChE decreases^62^. Thus, the new cholinesterase inhibitor **14** could act as a valuable therapeutic agent as a neuroprotector, especially in AD.

**Table 1:** AChE inhibition activity assay for compounds **5**–**17** in comparison to Donepezil and Physostigmine.

Compound no.	AChE IC_50_ (nM)^a^
**5**	> 100
**6**	> 100
**7**	90.02 ± 5.83
**8**	> 100
**9**	> 100
**10**	> 100
**11**	23.89 ± 1.41
**12**	60.65 ± 2.80
**13**	74.83 ± 3.06
**14**	11.57 ± 0.45
**15**	> 100
**16**	86.17 ± 2.14
**17**	77.43 ± 1.29
**Donepezil**	16.40 ± 1.03
**Physostigmine**	40.01 ± 2.01

IC_50_ values were calculated using non-linear regression analysis.

^a^Each value indicates the mean ± S.E (n = 3).

##### Free radical scavenging activity (DPPH) assay

In the present study, the DPPH assay was used for evaluating the free radical scavenging activity of all the newly synthesized compounds by measuring their ability to quench free radicals (Table 2). Compound **14** was able to reduce the stable radical DPPH to the yellow-colored diphenyl picryl hydrazine. Thus, compound **14** displayed significant concentration-dependent inhibition of DPPH activity, with IC_50_ 95.54 µM. compared to vitamin C (IC_50_ = 112.78 µM). These results indicate that compound **14** is considered a potent antioxidant as it could act as an electron or hydrogen donator to scavenge DPPH^⋅^ radicals. These results indicate that **14** has strong scavenging power. This complies with the previous studies that confirmed the free radical scavenging activity of quinazolinones bearing Sulfonamide derivatives^42,63^.

**Table 2:** Free radical scavenging activity using DPPH assay for all the newly synthesized compounds in comparison to ascorbic acid.

Compound no.	DPPH IC_50_ (µM)^a^
**5**	149.8 ± 1.26
**6**	143.36 ± 1.12
**7**	118.76 ± 0.92
**8**	176.56 ± 1.99
**9**	148.01 ± 1.53
**10**	154.92 ± 1.24
**11**	109.03 ± 1.09
**12**	122.31 ± 0.92
**13**	129.12 ± 1.03
**14**	95.54 ± 1.67
**15**	136.31 ± 1.30
**16**	137.35 ± 0.29
**17**	126.54 ± 1.93
**Ascorbic acid**	112.78 ± 1.03

IC_50_ values were calculated using non-linear regression analysis.

^a^Each value indicates the mean ± S.E (n = 3).

Heterocyclic compounds are well known for their capability to bind free radicals^64^. Furthermore, sulfonamides are known for their redox properties^65^, the 2-pyrazinyl derivative **14** has excellent antioxidant properties. Pyrazine derivatives have the potential to defend cells from oxidative harm caused by ionizing radiation through the transfer of electrons and hydrogen. The aminopyrazine derivatives are inhibitors of lipid peroxidation and good scavengers of peroxynitrite^66^. The mechanism by which compound **14** exerts its antioxidant activity is likely through its ability to donate the hydrogens of the NH_2_ group of benzenesulfonamide, thereby interacting with the divalent nitrogen atom of the DPPH and forming hydrazine DPPH-H^67^. These findings suggest that compound **14** has the potential to provide radioprotective effects.

#### In vivo evaluation

##### Lethal dose fifty (LD_50_)

Lethal dose fifty (LD_50_) was determined for the most promising compound **14** to estimate its acute toxicity in albino male mice. The LD_50_ value was found to be 300 mg/Kg (i.p.) bodyweight. Consequently, one-tenth of this dose was selected as the therapeutic dose for further evaluation of the potential radioprotective effects of **14**. This was following our previous studies^42,63,68^.

##### Behavior study

**Figure 3:**
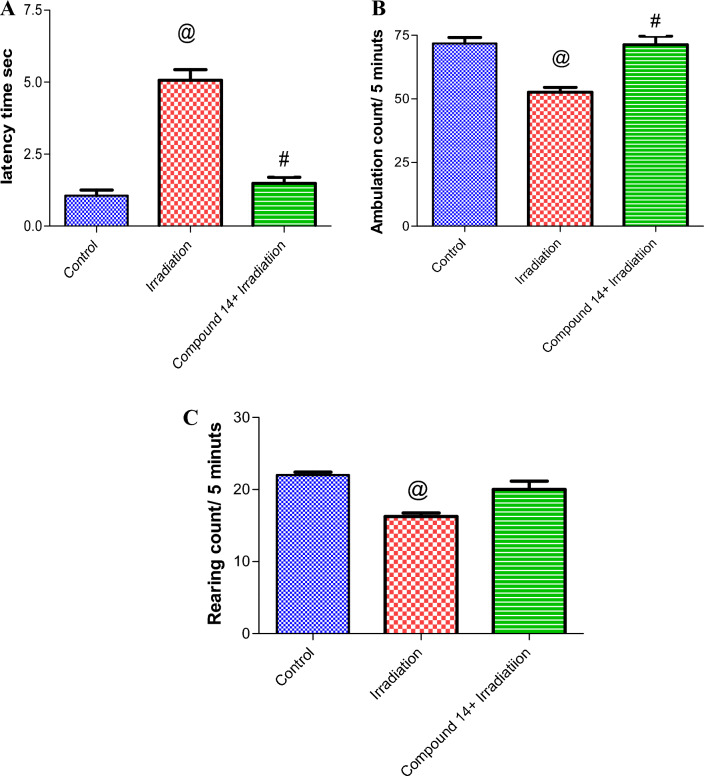
Effect of compound **14** on open field test: latency time (**A**), ambulation (**B**) and rearing (**C**). Each value represents mean ± SE. Statistical analysis was carried out by one way ANOVA followed by Bonferroni’s multiple comparison test. ^@^Significantly different from control group, ^#^significantly different from combined group (Radiation + compound **14**). P < 0.05 (n = 7).

*Open field test (OFT)* As shown in Fig. 3A, latency time was significantly elevated (4.8 folds) in the irradiation group in comparison with the control. Mice treated with **14** followed by irradiation exhibited a significant decrease in latency time by 72.4% compared with mice that received radiation alone. Furthermore, irradiation intensely decreased locomotor activity of mice expressed as ambulation (by 33%) as compared with control mice. However, compound **14** abolished the effect of radiation on ambulation (by 31%) (Fig. 3B). This complied with the reported studies^69,70^. Whereas, data revealed a decrease in rearing in radiation group relative to control (by 25.7%) (Fig. 3C). Remarkably, administration of **14** before irradiation normalized all open field parameters.

**Figure 4:**
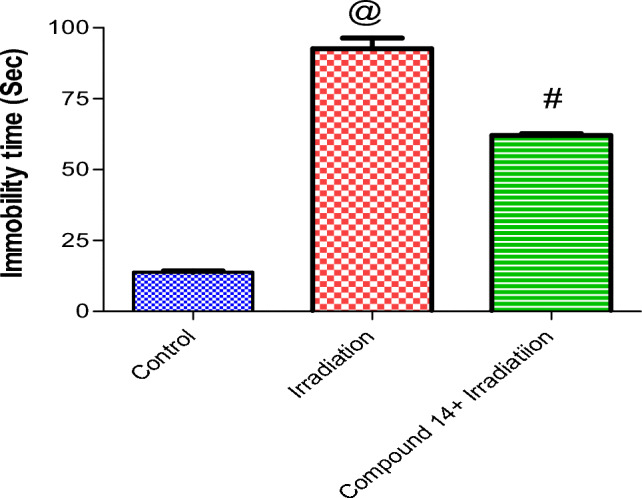
Effect of compound **14** on immobility time in a forced swimming test. Each value represents mean ± SE. Statistical analysis was carried out by one way ANOVA followed by Bonferroni’s multiple comparison test. ^@^Significantly different from control group, ^#^significantly different from combined group (Radiation + compound **14**). P < 0.05 (n = 7).

*Forced swimming test* As shown in Fig. 4, irradiation significantly increased immobility time in the forced swimming test (FST) 7 folds, as compared to the control group. The prior administration of **14** significantly decreased the mean immobility time in mice as compared to animals that received radiation only (by 33%)^71^.

##### Biochemical parameters investigated in brain tissue homogenate

**Figure 5:**
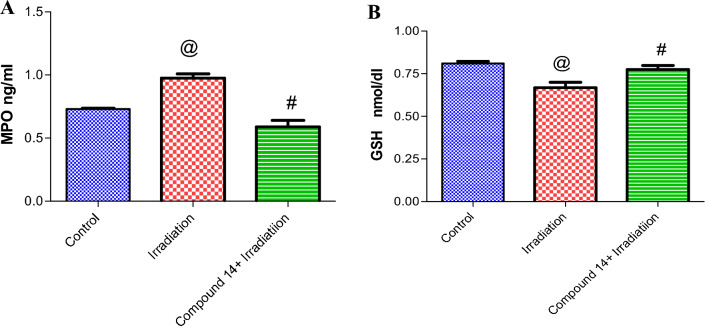
(**A**) Effect of compound **14** on Myeloperoxidase (MPO). (**B**) Glutathione (GSH). Each value represents mean ± SE. Statistical analysis was carried out by one way ANOVA followed by Bonferroni’s multiple comparison test. ^@^Significantly different from control group, ^#^significantly different from combined group (Radiation + compound **14**). *P* < 0.05. (n = 7).

A significant increase in MPO levels by 33.5% as well as a decrease in the levels of GSH by 18%, was observed in irradiated mice brains when compared to the non-irradiated group. Treatment of irradiated mice with **14** led to a decrease in MPO by 39.6% and an increase in GSH levels by 17% respectively, as compared to irradiated mice (Fig. 5A,B).

**Figure 6:**
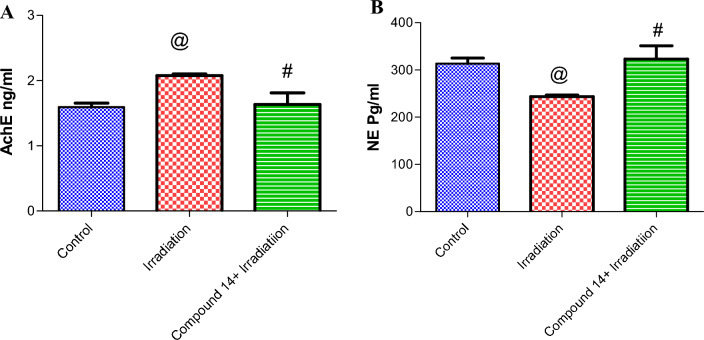
(**A**) Effect of compound **14** on Acetylcholinesterase (AChE). (**B**) Norepinephrine (NE). Each value represents mean ± SE. Statistical analysis was carried out by one way ANOVA followed by Bonferroni’s multiple comparison test. ^@^Significantly different from control group, ^#^significantly different from combined group (Radiation + compound **14**). P < 0.05. (n = 7).

As shown in Fig. 6A, irradiation significantly increased AChE by 24%, as compared to the control group. The prior administration of **14** significantly decreased AChE by 21% as compared to the animals that received radiation only. Besides, irradiation intensely decreased Norepinephrine (NE) by 22% as compared with control mice. However, compound **14** abolished the effect of radiation on ambulation by 25% (Fig. 6B).

It is well-known that radiation generates excessive amounts of reactive oxygen species (ROS) leading to oxidative stress which is responsible for tissue damage following irradiation^72,73^. Oxidative stress (OS) plays a critical role in the pathophysiology of several brain-related disorders, including neurodegenerative diseases and ischemic stroke, which are the major causes of dementia^74,75^. The present study revealed amplified MPO activity in the brain samples of irradiated mice, which indicates the induction of inflammatory response^76^. Besides, the reduction in brain antioxidant status in mice is evidenced by depleted GSH levels, mostly due to its consumption by ROS^77^. GSH is a free radical scavenger that exerts its antioxidant function by reaction with superoxide radicals, hydrogen peroxide (H_2_O_2_), peroxide free radicals (OH˙), and singlet oxygen that are considered important inhibitors of free radical arbitrated lipid peroxidation^78,79^ since membranes within the brain are known to be rich in peroxidizable fatty acids, thus they undergo peroxidation under oxidative damages^80–82^. It is well-considered that inflammation underlies a wide range of pathological processes and is closely linked to oxidative stress via generation of ROS that can prolong and augment inflammatory cascades and prompt tissue damage^83^. Inflammation is involved in the pathogenesis of radiation-induced brain injury^84,85^.

In this study, the iodoquinazolinone derivative **14** revealed beneficial effects in relieving oxidative damage, as compound **14** administration boosted GSH levels as compared to irradiated group, which could be attributed to its high scavenging power as potent antioxidant findings. As proved in current study findings (in vitro DPPH assay) appears to support this idea. This coincided with other previous studies that reported the antioxidant ability of quinazolinones derivatives to improve the antioxidant status in irradiation-induced hepatotoxicity^63,68^. Previous studies reported that iodoquinazolinones ameliorated oxidative/antioxidant parameters after liver injury in rats^42^. In the current study, the observed significant upsurge in AChE activity and depletion of NA level in irradiated animals is an indication of neuro-damages. Previous investigators accounted for comparable data^29,86,87^.

Acetylcholinesterase (AChE) plays a key role in cholinergic transmission by catalyzing the hydrolysis of the neurotransmitter acetylcholine which is an essential neurotransmitter in the regulation of motor function and locomotion^88,89^. The use of AChE inhibitors elicits several responses, which mediate the symptoms of Alzheimer’s disease^39^. The in vitro study indicates that **14** is the most active AChE inhibitor in this series. Likewise, the administration of **14** restored AChE activity to basal levels, which could be one of the mechanisms involved in mitigating neurobehavioral dysfunction. Furthermore, the observed decline in MPO and AChE activity after treatment with **14** indicates amelioration of inflammation and consequently, improving cholinergic neurotransmission and restoring locomotor functions as that confirmed through behavioral evaluation findings in the current study. These results are in concordance with^90–92^.

Thus, current research has shed light on the effects of iodoquinazolinone-bearing sulfonamide derivative **14** as a promising antioxidant and neuroprotector. These neuroprotective effects should be kept in mind for further research to evaluate the possible therapeutic use for Alzheimer's treatment.

### Molecular docking

#### Docking on myeloperoxidase

**Figure 7:**
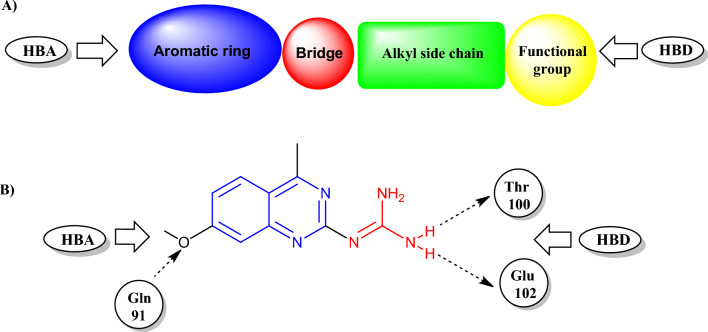
(**A**,**B**) Structural elements of MPO inhibitor (2-(7-methoxy-4-methyl-quinazolin-2-yl)guanidine) in the MPO active site.

To explore the binding mode of the designed iodoquinazolinone derivatives, we obtained and analyzed the docked structures of the synthesized compounds within the catalytic site of MPO and compared it with that co-crystallized ligand 2-(7-methoxy-4-methylquinazolin-2-yl)guanidine (Fig. 7A,B). Soubhye et al*.*^93^ reported that the 2-(7-methoxy-4-methylquinazolin-2-yl)guanidine was found to be a mechanism-based MPO inhibitor of high inhibitory potency. It achieved the best binding mode with a remarkable docking score of − 16.20 kcal/mol. The binding mode of the aforementioned guanido compound, with the receptor, revealed the interaction between the amino group of guanidine (Hydrogen bond donor HBD) attached to Thr100 and Glu102 through a hydrogen bond. While the methoxy group (Hydrogen bond acceptor HBA) of the fused benzene ring attached to Gln91.

**Figure 8:**
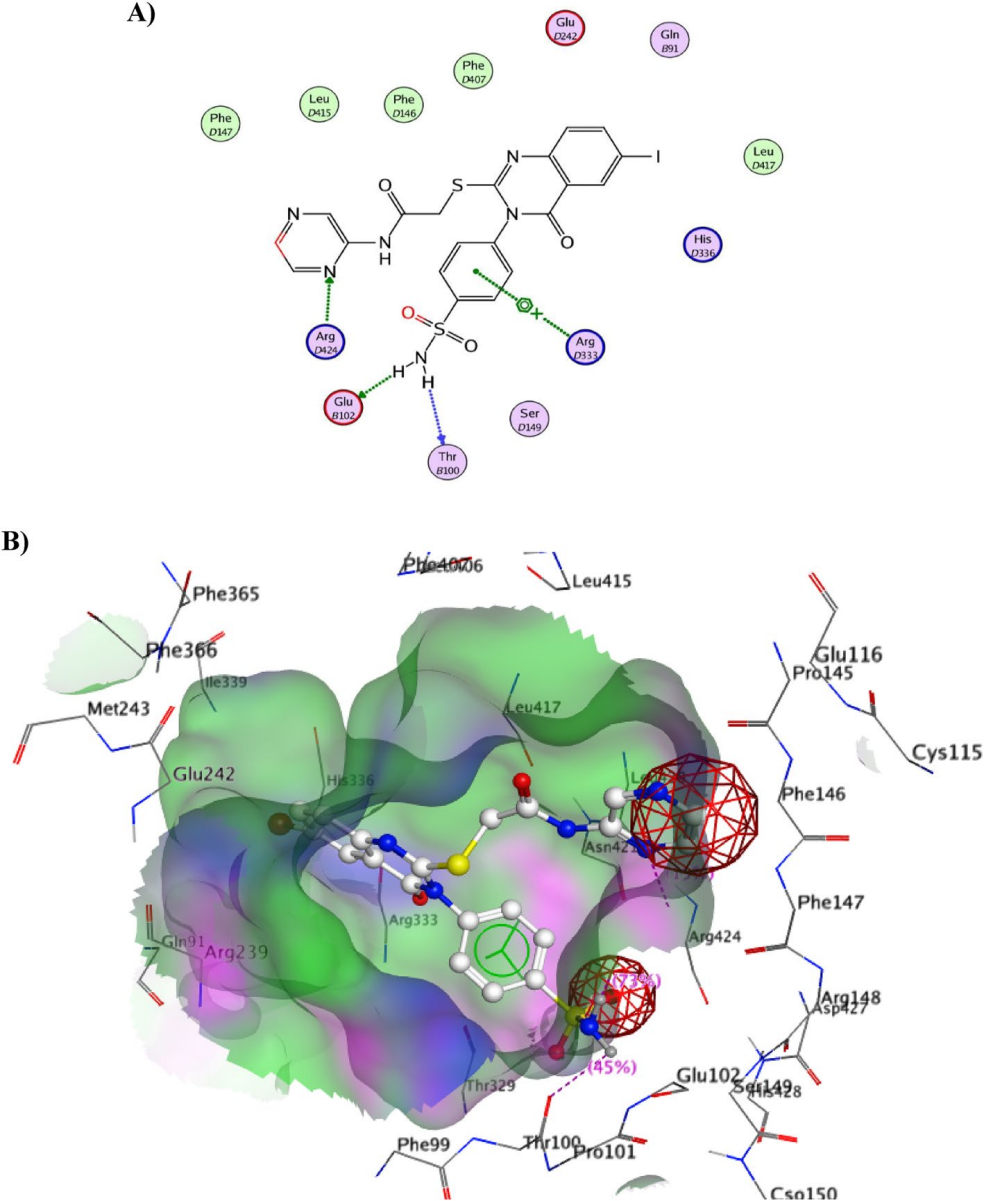
(**A**) 2D view of **14** and **(B**) 3D surface view of **14** inside the active site of MPO (4C1M).

Compound **14** binds to the active site of MPO in the same manner as that of the native ligand, through the amino group of sulfonamide (have the same role as guanido group) binds to Thr100 and Glu102 as hydrogen bond donar, the benzene of sulfonamide group with Arg333 through a cation-pi interaction. Also, the nitrogen of pyrazine ring acts as HBA group and is attached to Arg424^93^ (Fig. 8). Table 3 revealed the binding interactions and energy scores of the co-crystallized ligand and compound **14** inside the active site of MPO.

**Table 3:** Docking scores of the co-crystallized ligand and compound **14** inside the active site of 4C1M and 4M0E enzymes.

Receptor	Compound no.	Scores (Kcal/mol)	Amino acids	Interacting groups	Length (Å)
4C1M	Ligand	− 11.46	Thr 100	NH_2_ of guanidine	2.51
			Glu 102	NH_2_ of guanidine	2.59
			Gln 91	Methoxy group	3.11
	14	− 10.91	Thr 100	NH_2_ of sulfonamide	2.91
			Glu 102	NH_2_ of sulfonamide	2.57
			Arg 333	NH of sulfonamide	2.29
			Arg 424	N of pyarazine	2.43
4M0E	Ligand	− 9.74	Arg 296	CO benzofuran	2.35 2.41 3.13 3.10
			Ser 293	CO benzofuran	
			Trp 286	Benz. naphthol	
	14	− 9.88	Arg296	O of sulfonamide	2.73
			Trp 286	Benz. sulfonamide	2.43
			Tyr 124	N of pyrazine	2.22
			Tyr 341	Pyrazine ring	2.39

#### Docking on acetylcholinesterase

**Figure 9:**
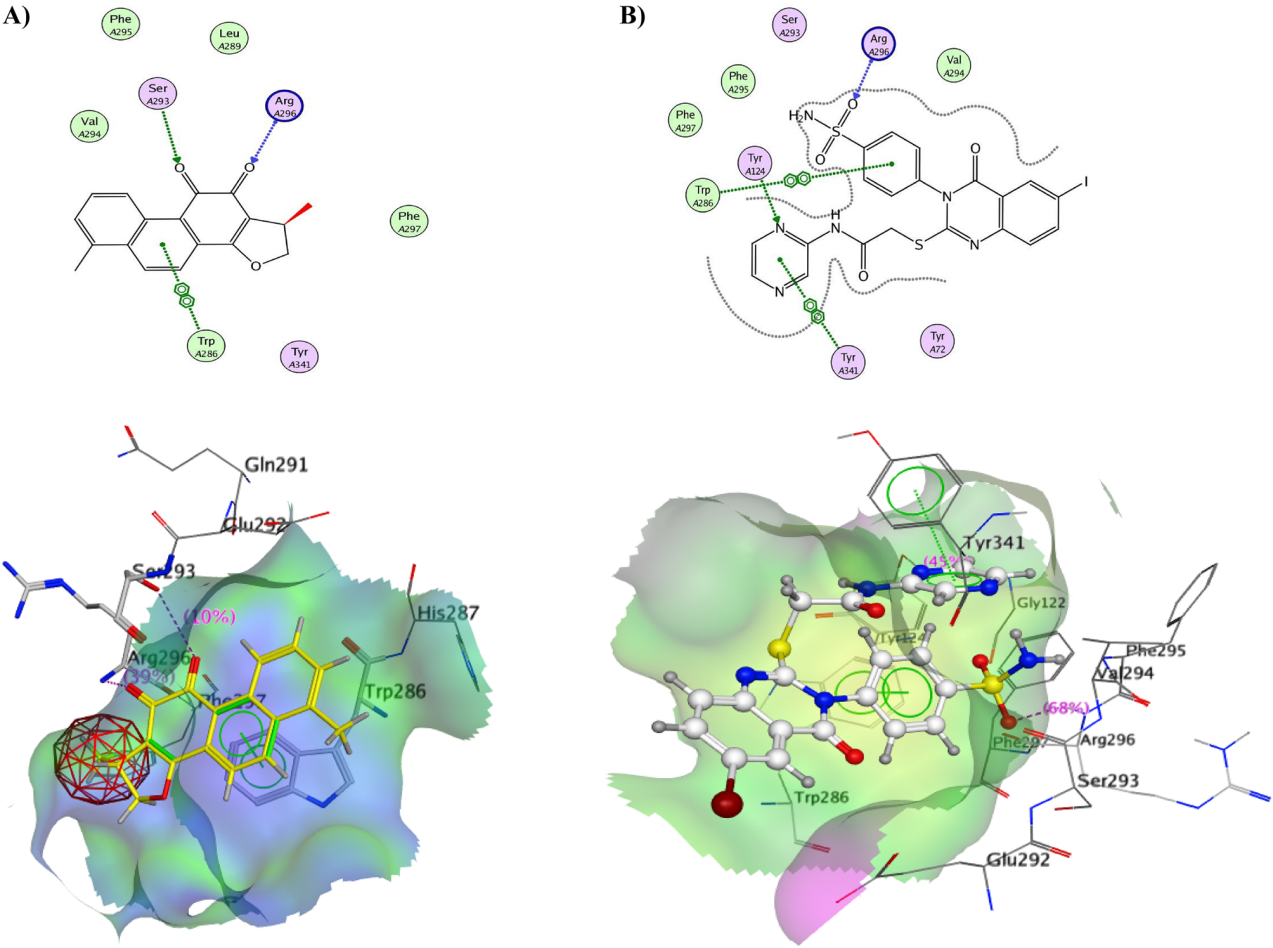
(**A**) 2D view and 3D surface view of docking validation and binding mode of the lead compound (yellow) in AChE pocket 4M0E. (**B**) 2D view and 3D surface view of **14** inside AChE pocket 4M0E.

The hypothetical binding modes of the most active compound **14** were calculated using MOE docking in the active site of AChE-dihydrotanshinone I complex. Based on the structural similarity between **14** and acetylcholinesterase inhibitors, a crystal structure of the inactive conformation of AChE (PDB: 4M0E) was chosen. This crystal structure was also selected because of the similar structural motifs in the tested compound and the structure of the co-crystallized ligand, (1R)-1,6-dimethyl-1,2-dihydronaphtho[1,2-*g*][1]benzofuran-10,11-dione (Dihydrotanshinone I). First, the docking algorithm was validated for its ability to reproduce the co-crystal binding mode of the reference compound. The reference compound was extracted from the complex and re-docked in the active site using the same parameters that will be used for the tested compound. The calculated binding modes of **14** revealed very interesting results as shown in Fig. 9A,B, the oxygen atom of sulfonamide moiety binds to the Arg296 by one hydrogen bonding. The benzene ring attached to sulfonamide moiety gives a hydrophobic interaction with Trp286. On the other hand, another hydrophobic binding interaction occurred between Tyr341 and pyrazine ring. The nitrogen atom of the pyrazine ring is attached through a hydrogen bond to Tyr124. Figure 9A,B demonstrated the binding mode of dihydrotanshinone and its overlay with the re-docked lead compound. Table 1 revealed the energy scores and binding interactions of the native ligand and **14** inside the active site of AChE.

## Conclusion

In summary, a hybridization strategy was adopted using the iodoquinazolinone scaffold and benzenesulfonamide moiety to produce the 2-((6-iodo-4-oxo-3-(4-sulfamoylphenyl)-3, 4-dihydroquinazolin-2-yl)thio)-*N*-(substituted) acetamide derivatives **5–17**. Different substitutions were introduced to the acetamide group to study the SAR. The structures of the synthesized compounds were confirmed based on their spectral and microanalytical data. All the newly synthesized compounds were screened for their potential AChE inhibitory and antioxidant activities. The 2-pyrazinyl derivative **14** was the most active scaffold that possessed the greatest inhibitory activity in the AChE assay with IC_50_ = 11.57 nM. Its antioxidant activity was estimated using DPPH assay (IC_50_ = 95.54 µM) in comparison to ascorbic acid (IC_50_ = 112.78 µM). Compound **14** showed relative safety with a median lethal dose of 300 mg/kg. The possible antioxidant and neuroprotective activities of compound **14** were evaluated in irradiated mice. Before irradiation, compound **14** was guarded against the changes in all the measured parameters. Also, it was able to cause mitigation of gamma radiation-induced oxidative stress verified by the decline in MPO and increase in GSH levels. It was able to modify the brain neurotransmitters (NA). Finally, molecular docking of **14** inside the active sites of MPO and AChE proved the same binding interactions as that of the co-crystallized ligands confirming its possible inhibitory effect against both receptors. These outcomes hold great promise for the use of compound **14** as a safe antioxidant agent and its inhibition of cholinesterase is of interest concerning neurodegenerative disorders such as Alzheimer’s disease. Future preclinical investigations would be carried out to confirm the specific and exact mechanism of action.

### Supplementary Information

Supplementary Information.

## Data Availability

All data generated or analyzed for this study are included in this published paper (and its Supplementary Information files). All biologically measured parameters were done using ELISA kits and the kit number is illustrated in the biological experimental section.
