# Supplementary figures and images for: Novel iodoquinazolinones bearing sulfonamide moiety as potential antioxidants and neuroprotectors
Source: Sci Rep. 2023 Sep 20;13:15546. doi: 10.1038/s41598-023-42239-2 (PMC10511408; doi:10.1038/s41598-023-42239-2)

**Supplementary Data**


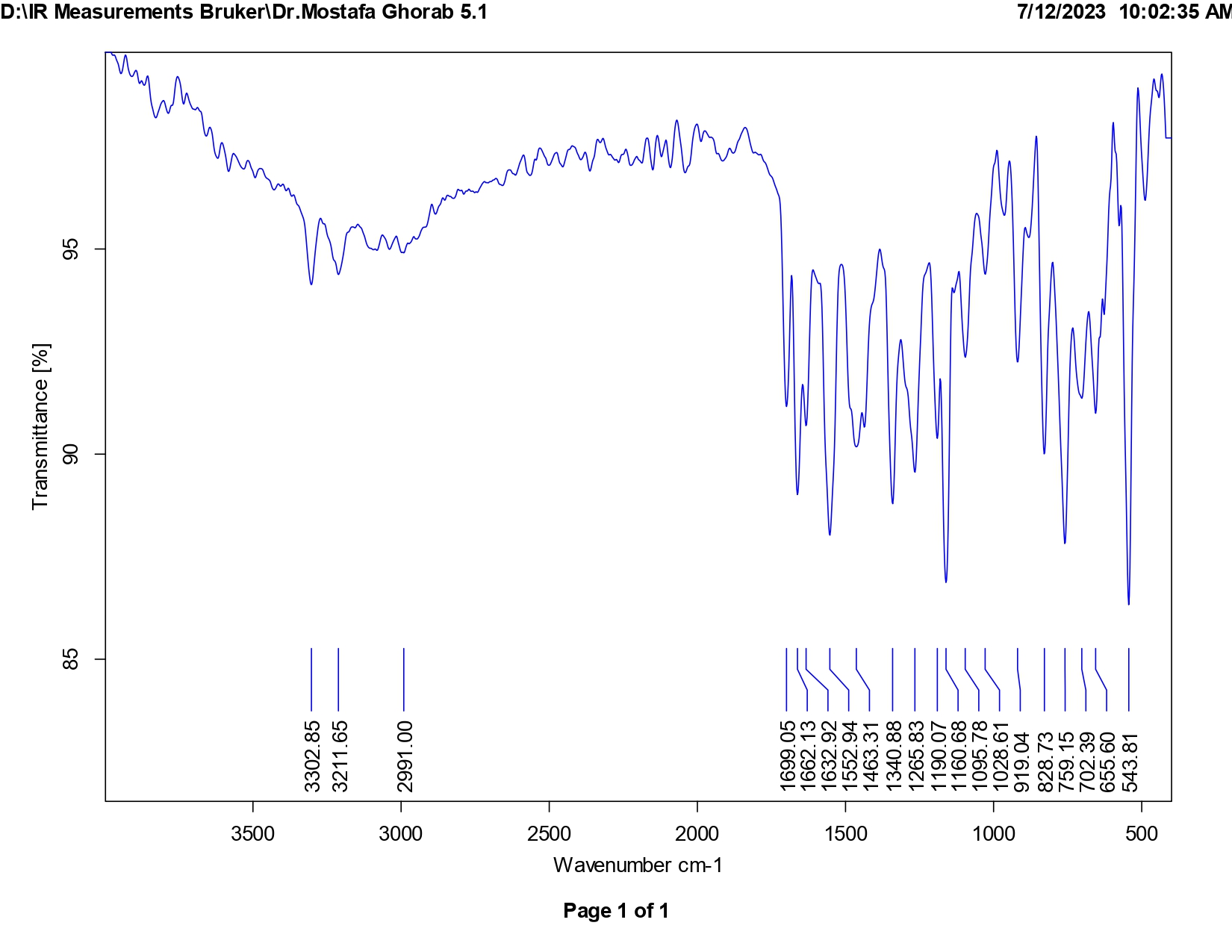


**
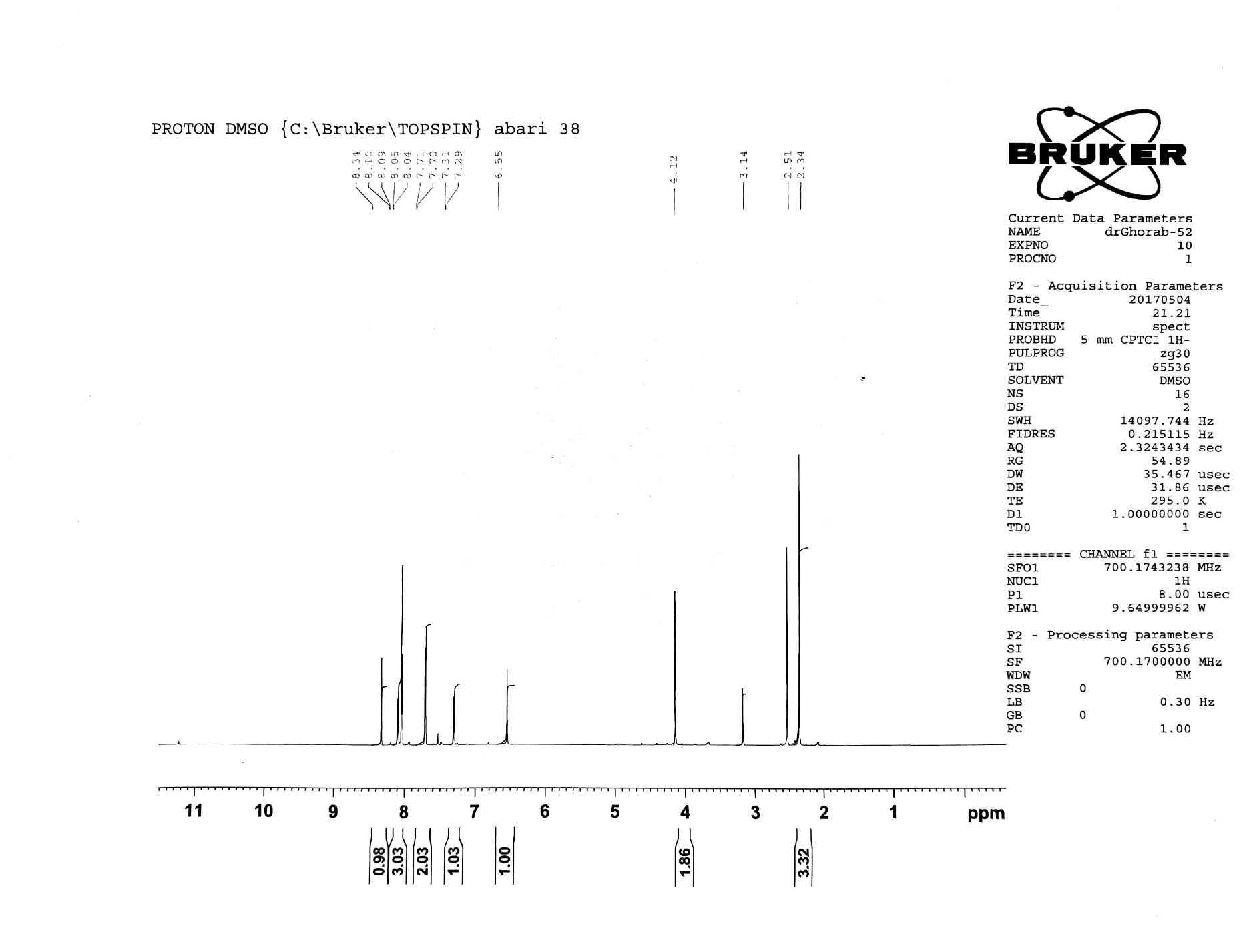
**


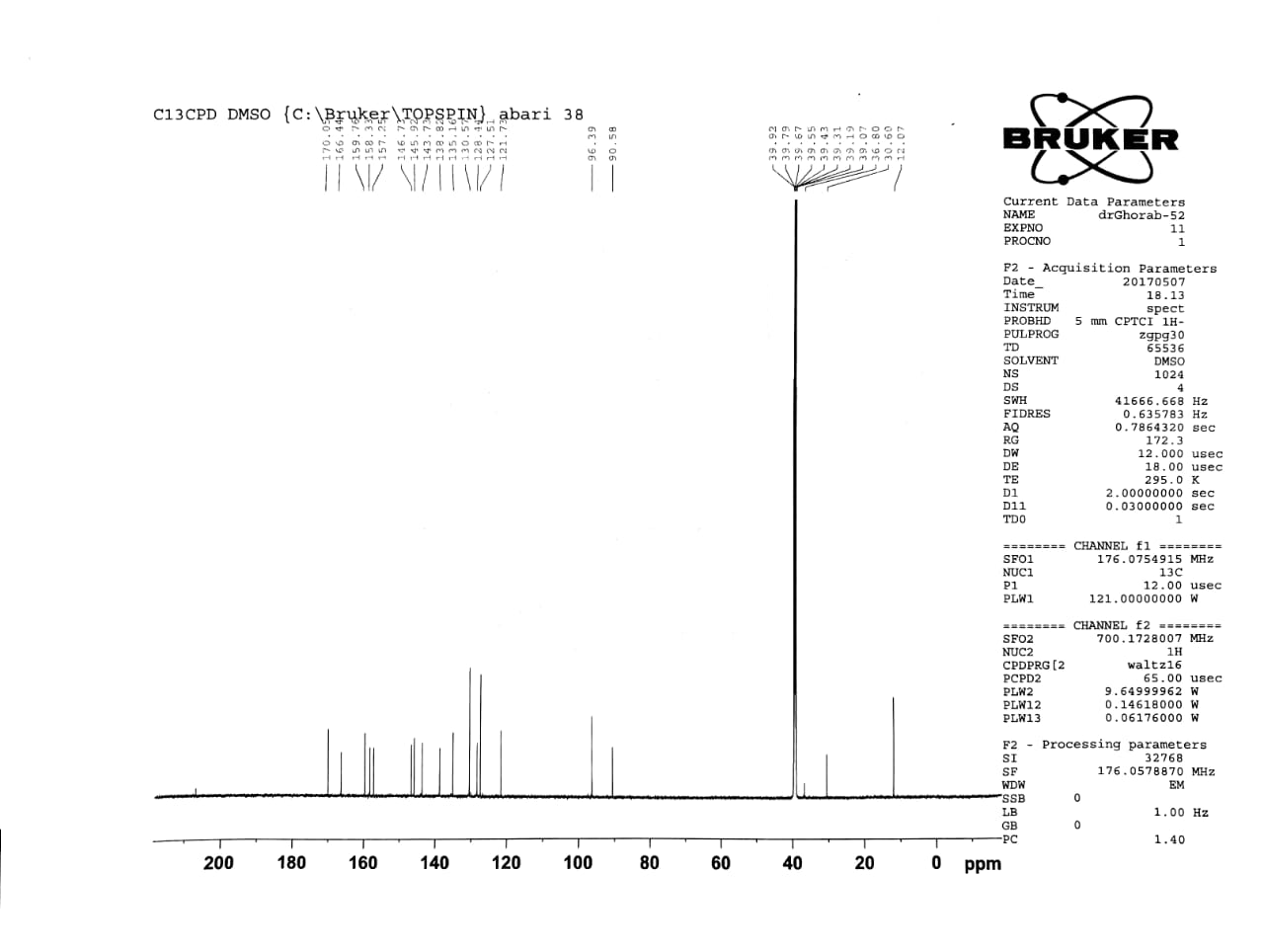


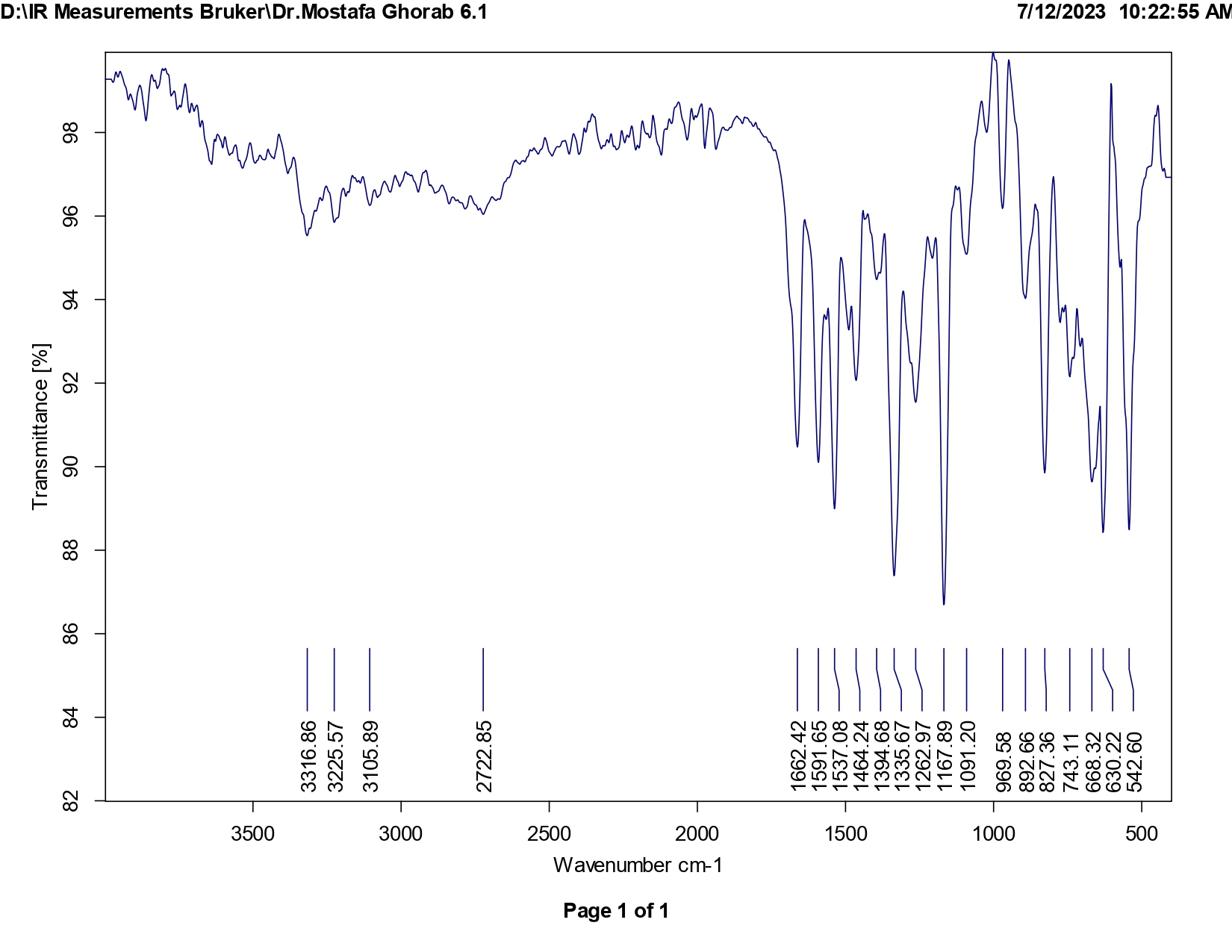


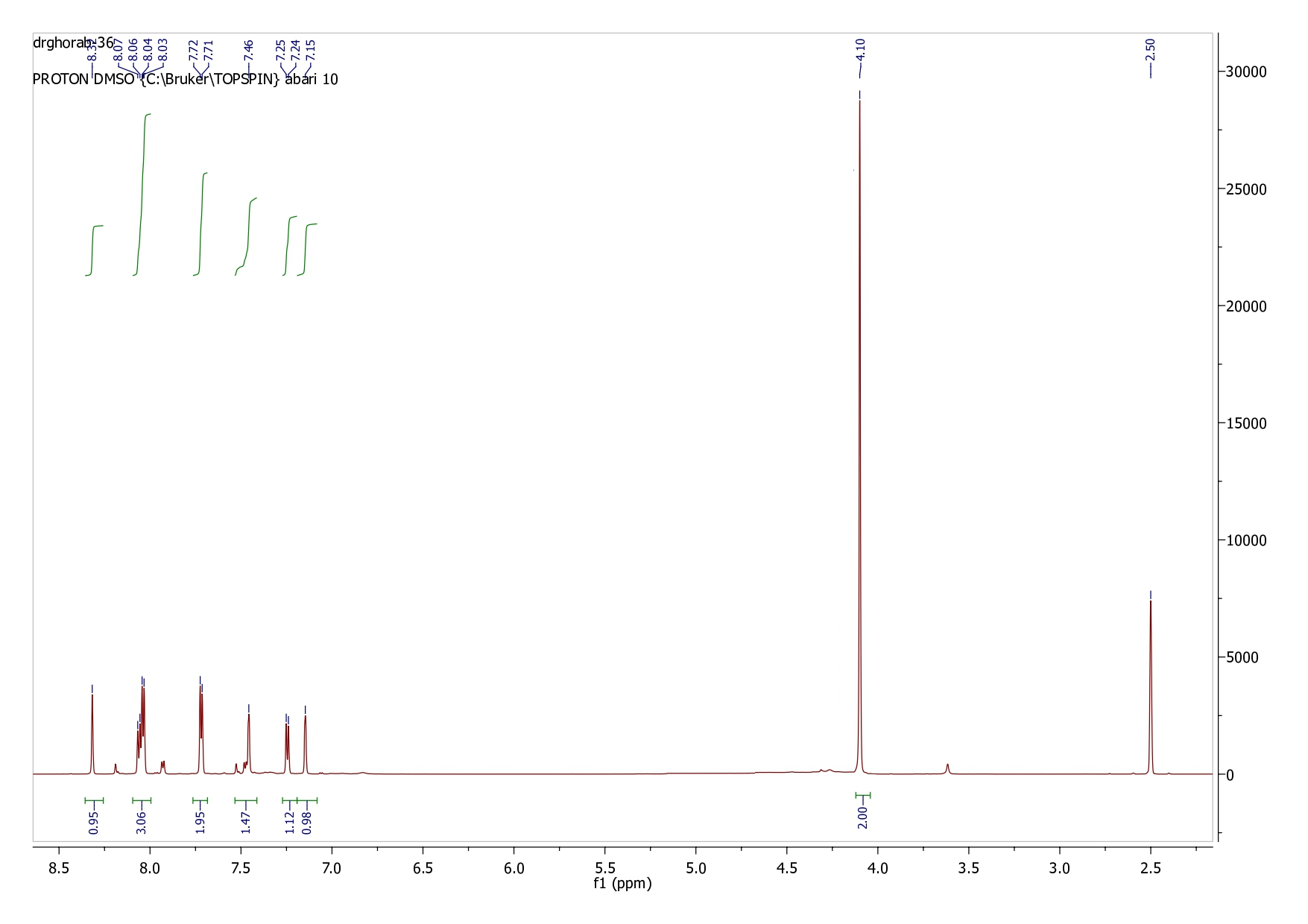


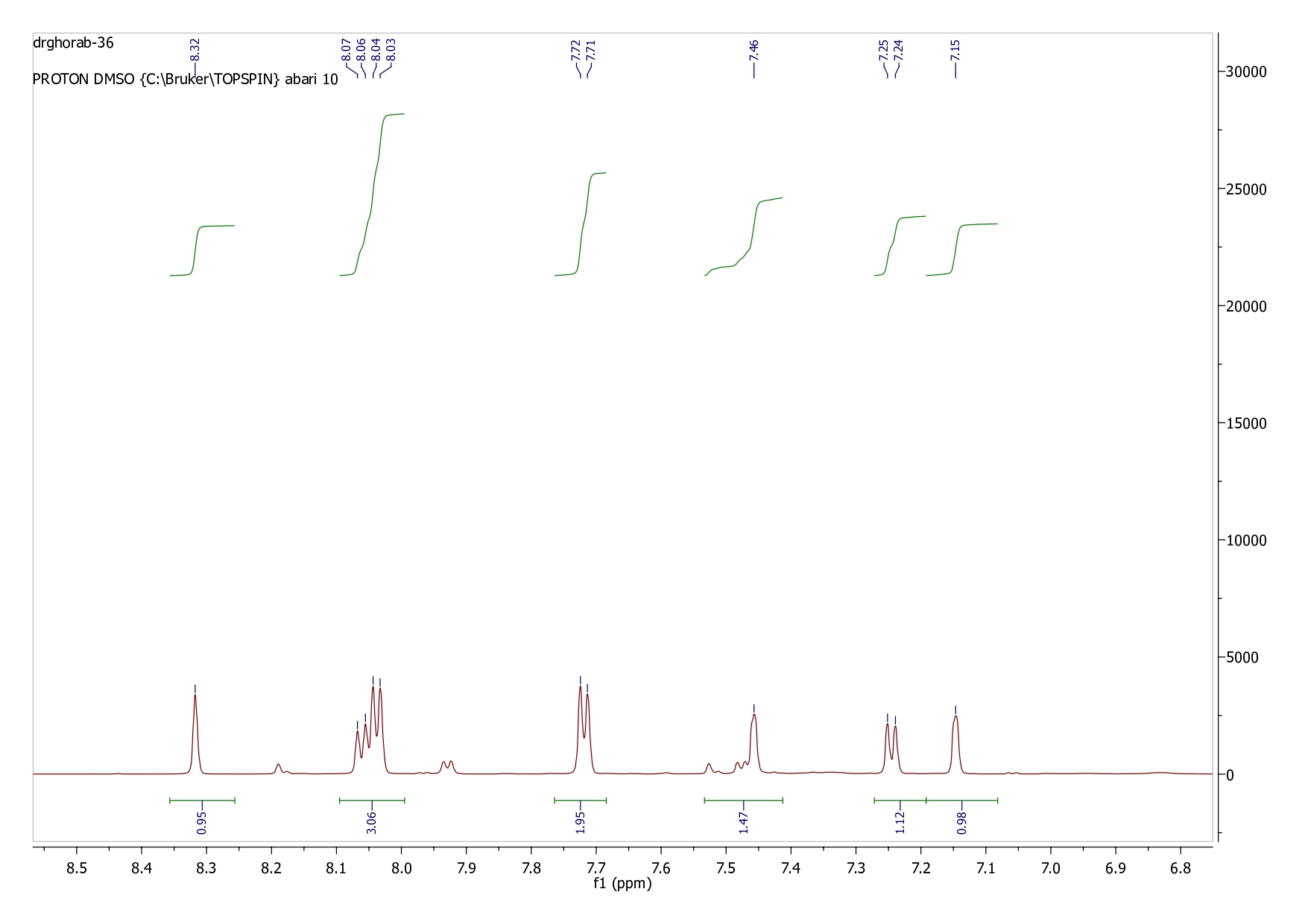


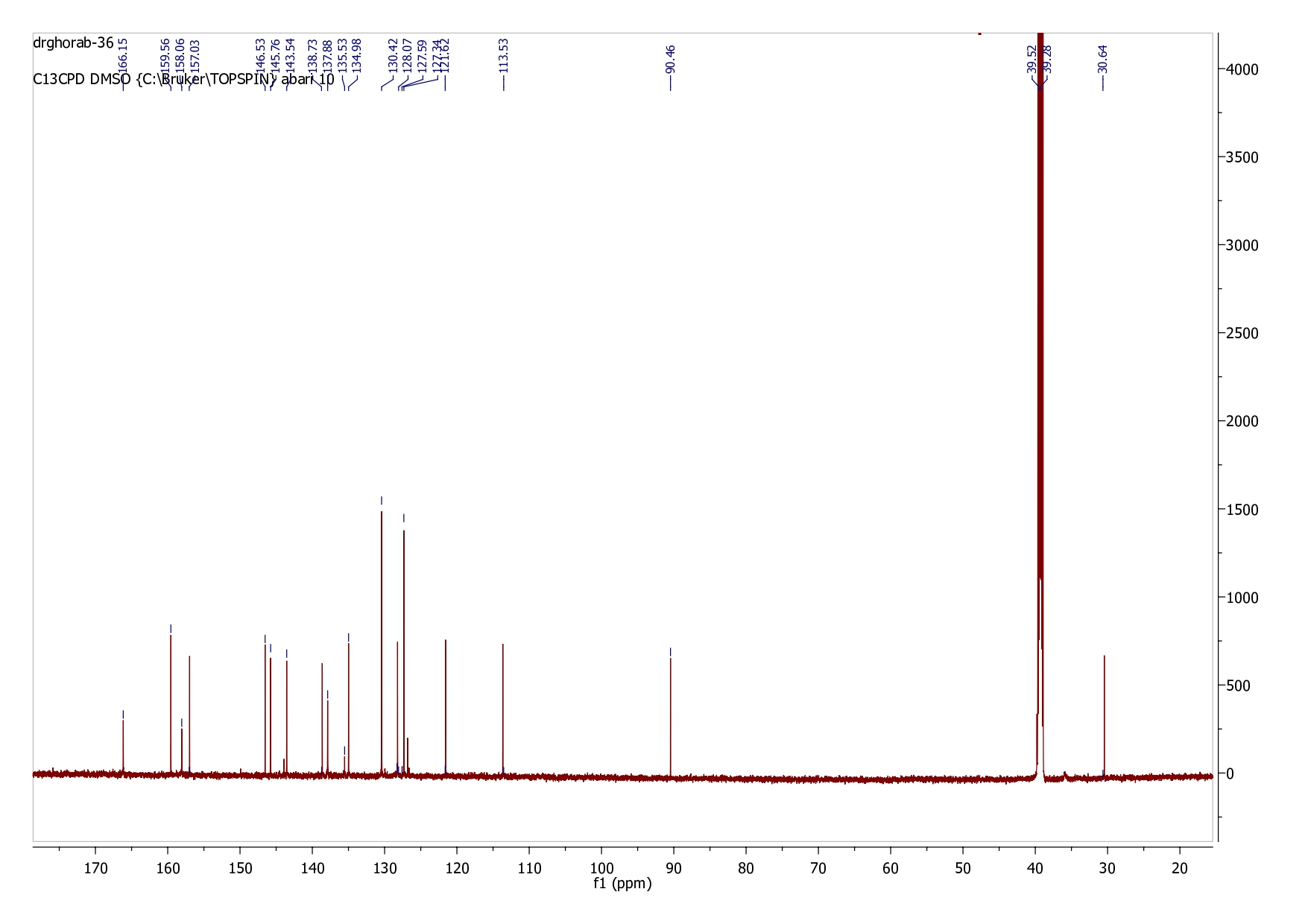


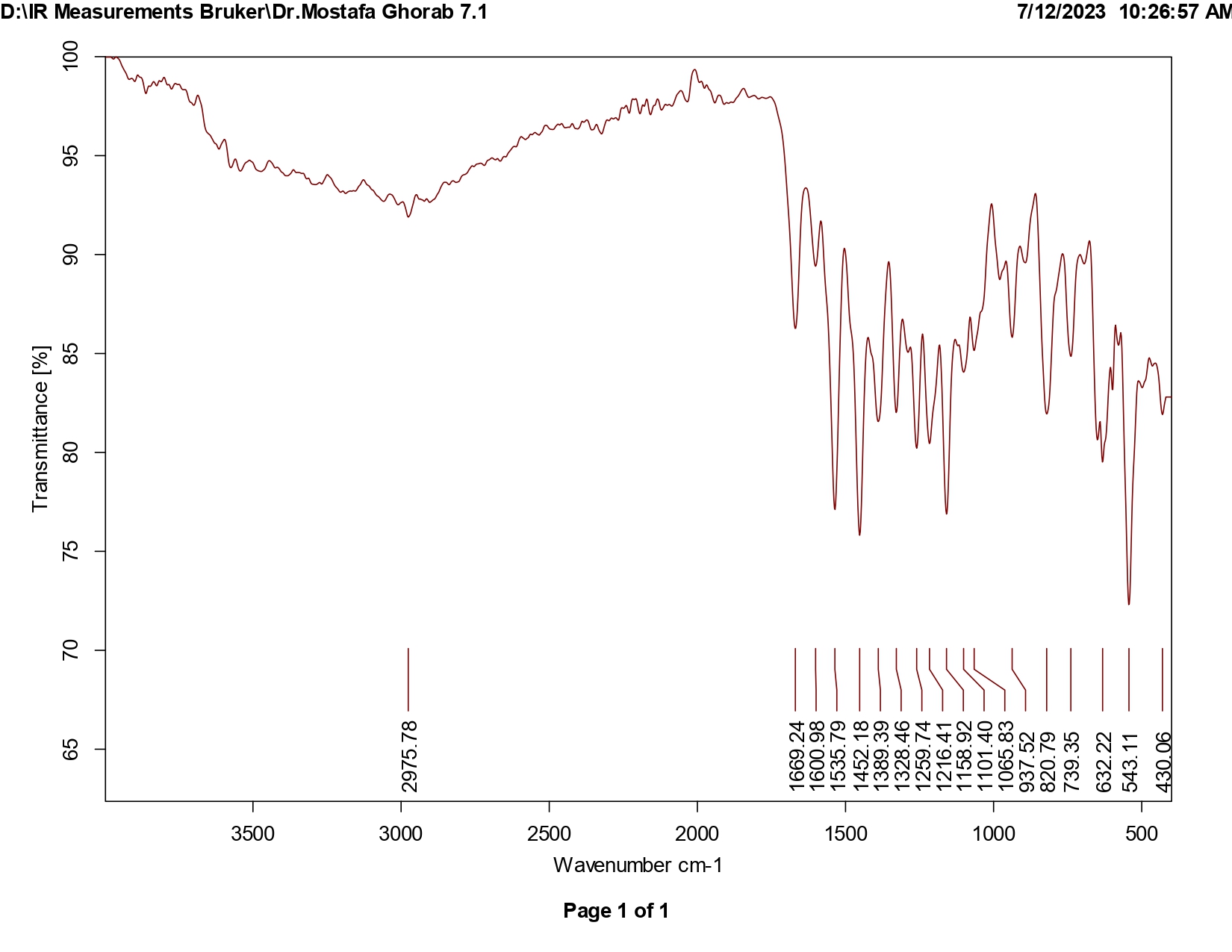

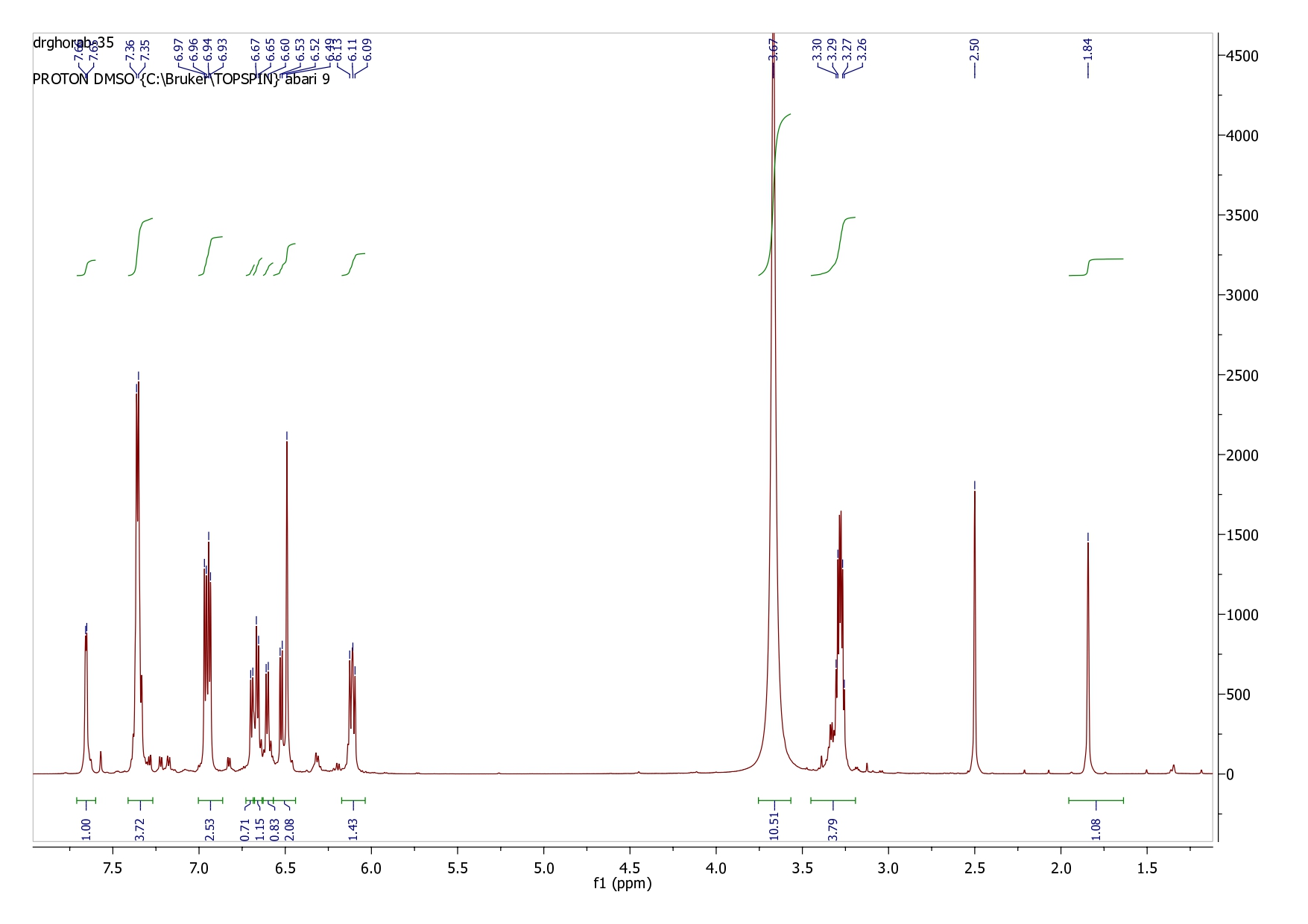


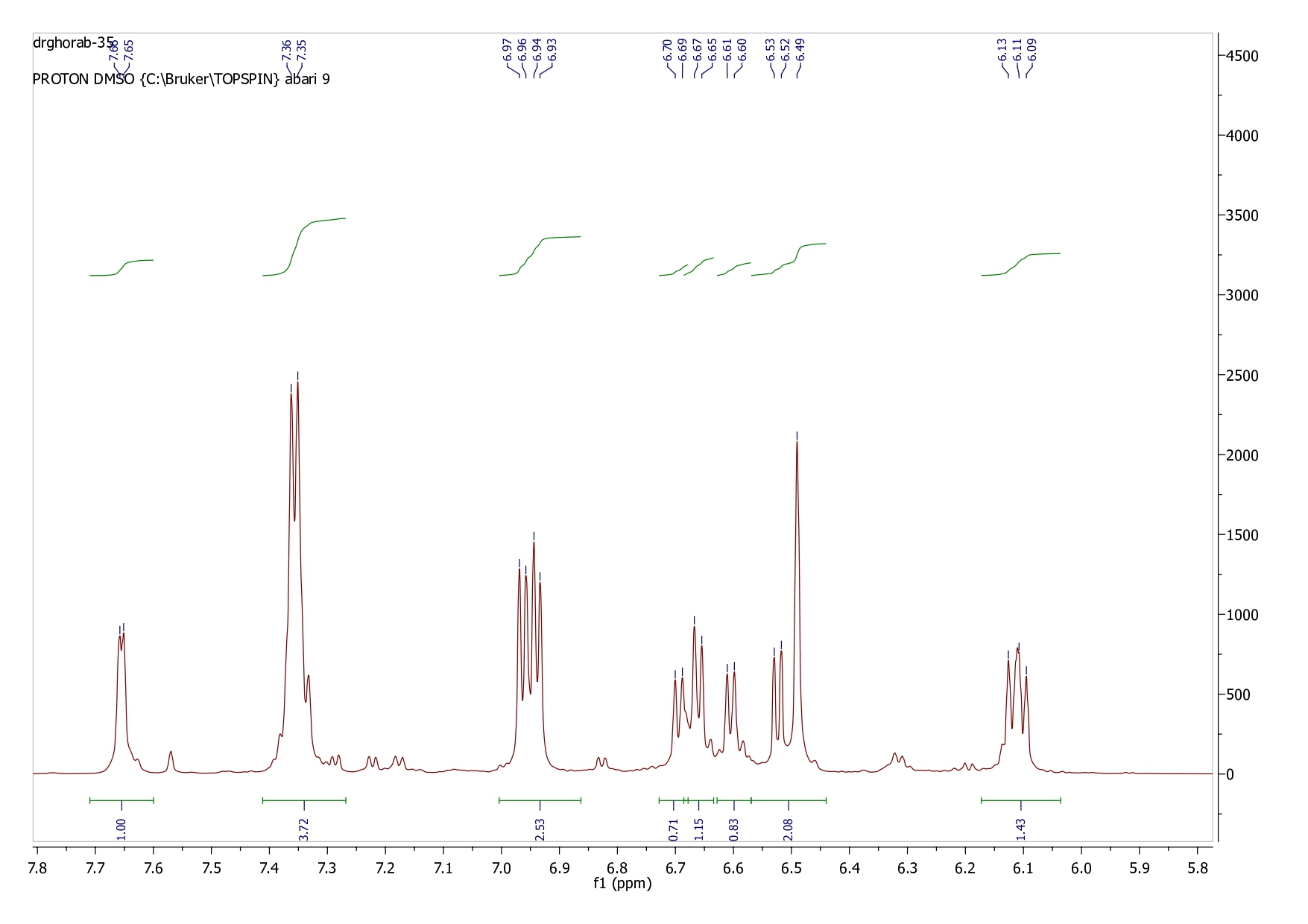


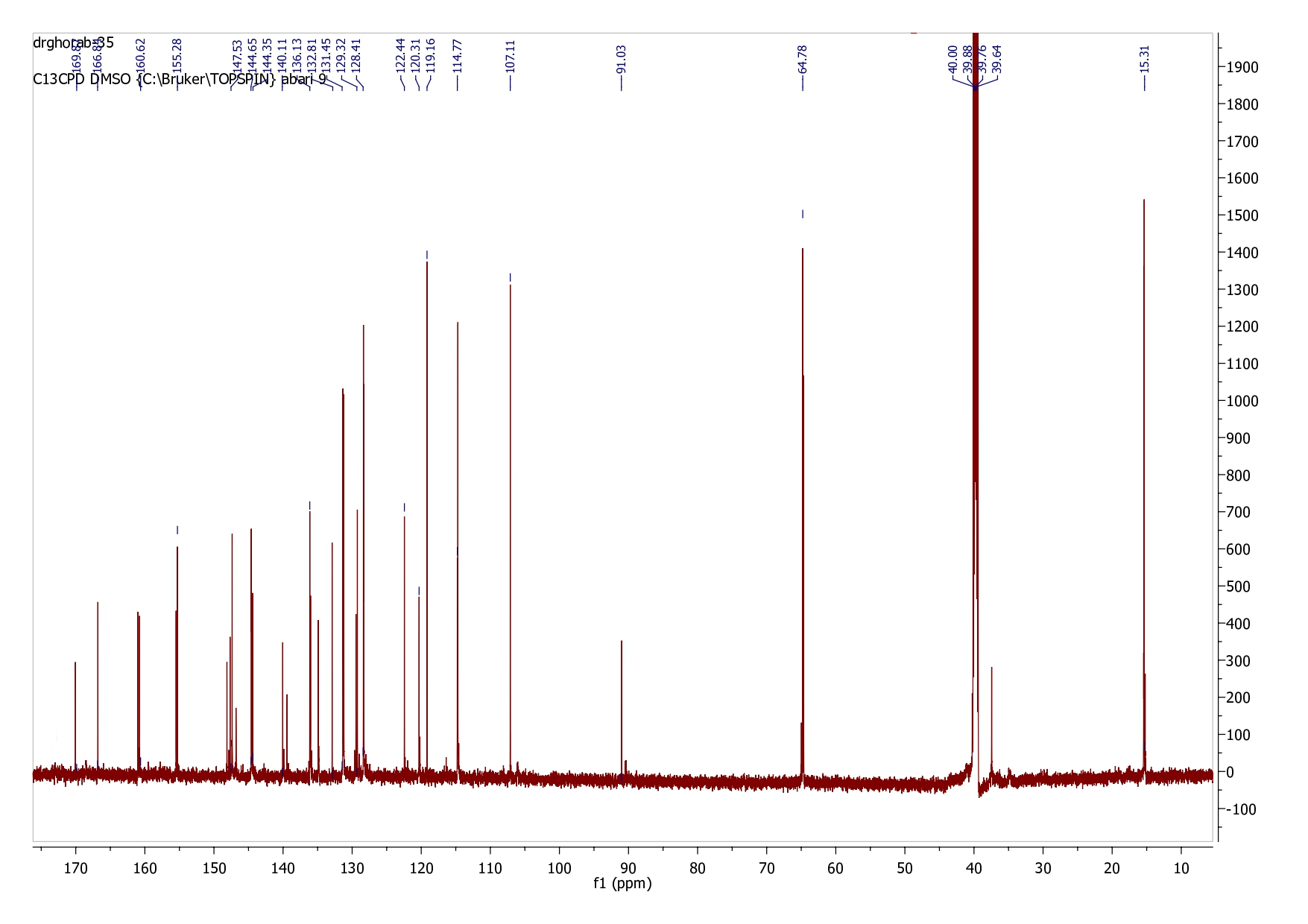


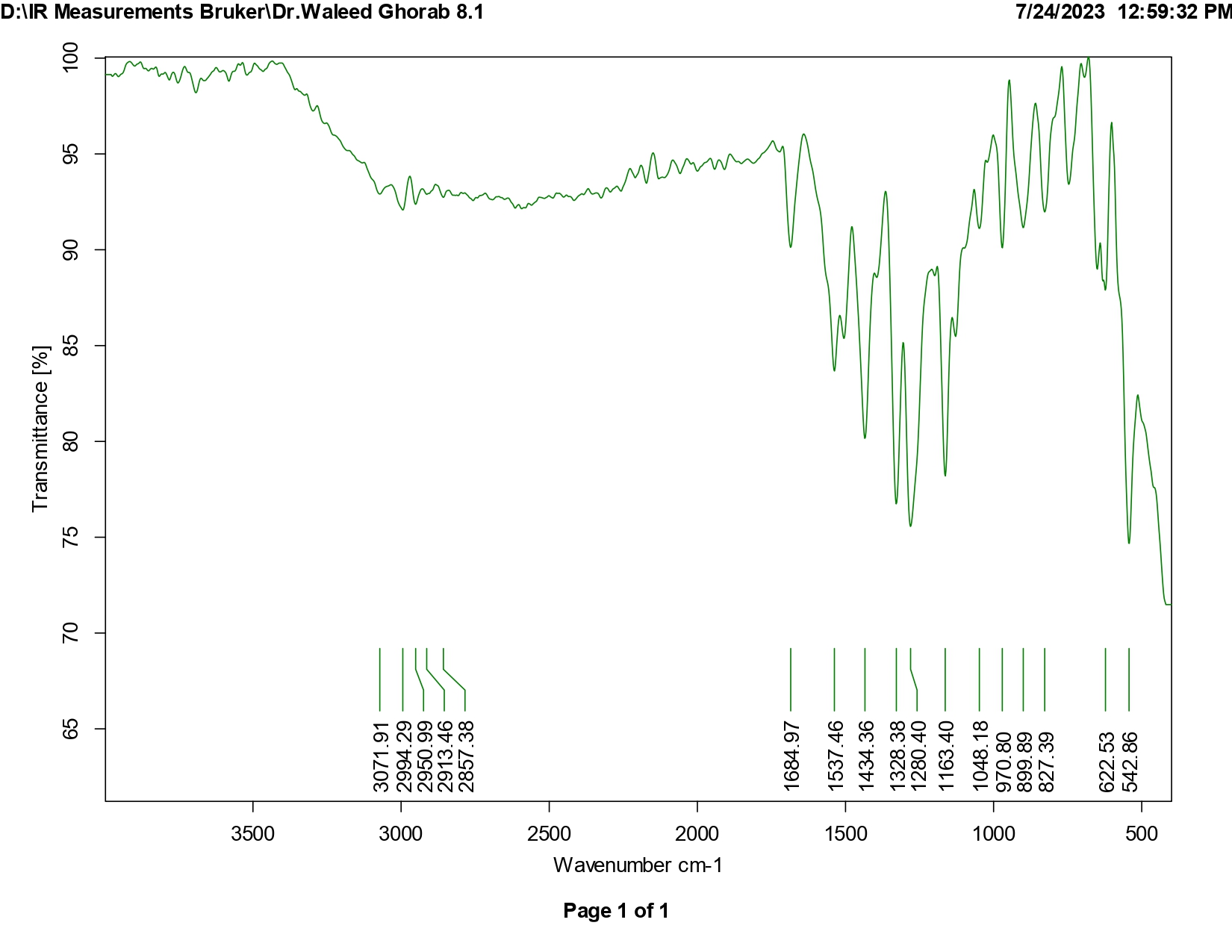


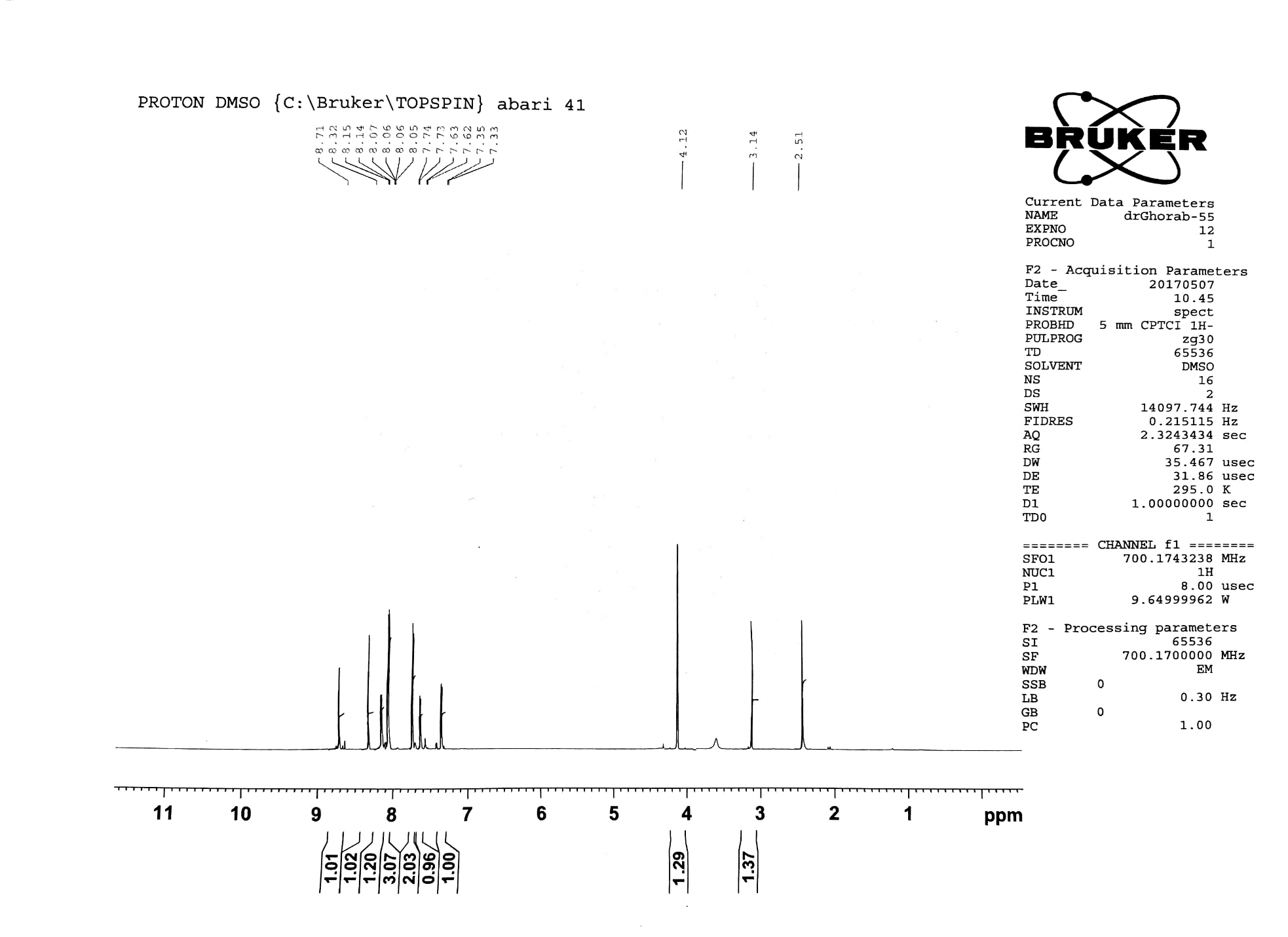


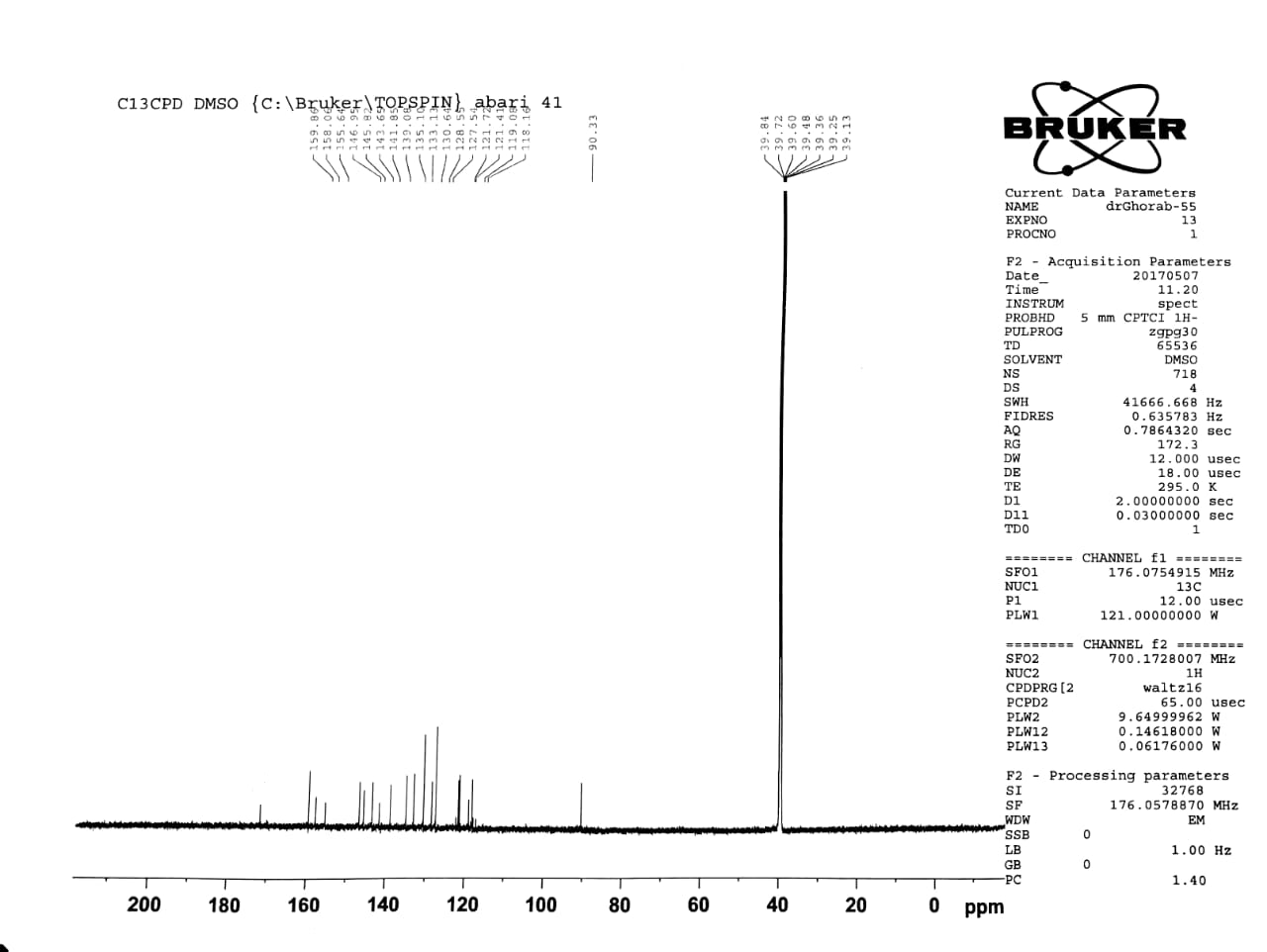


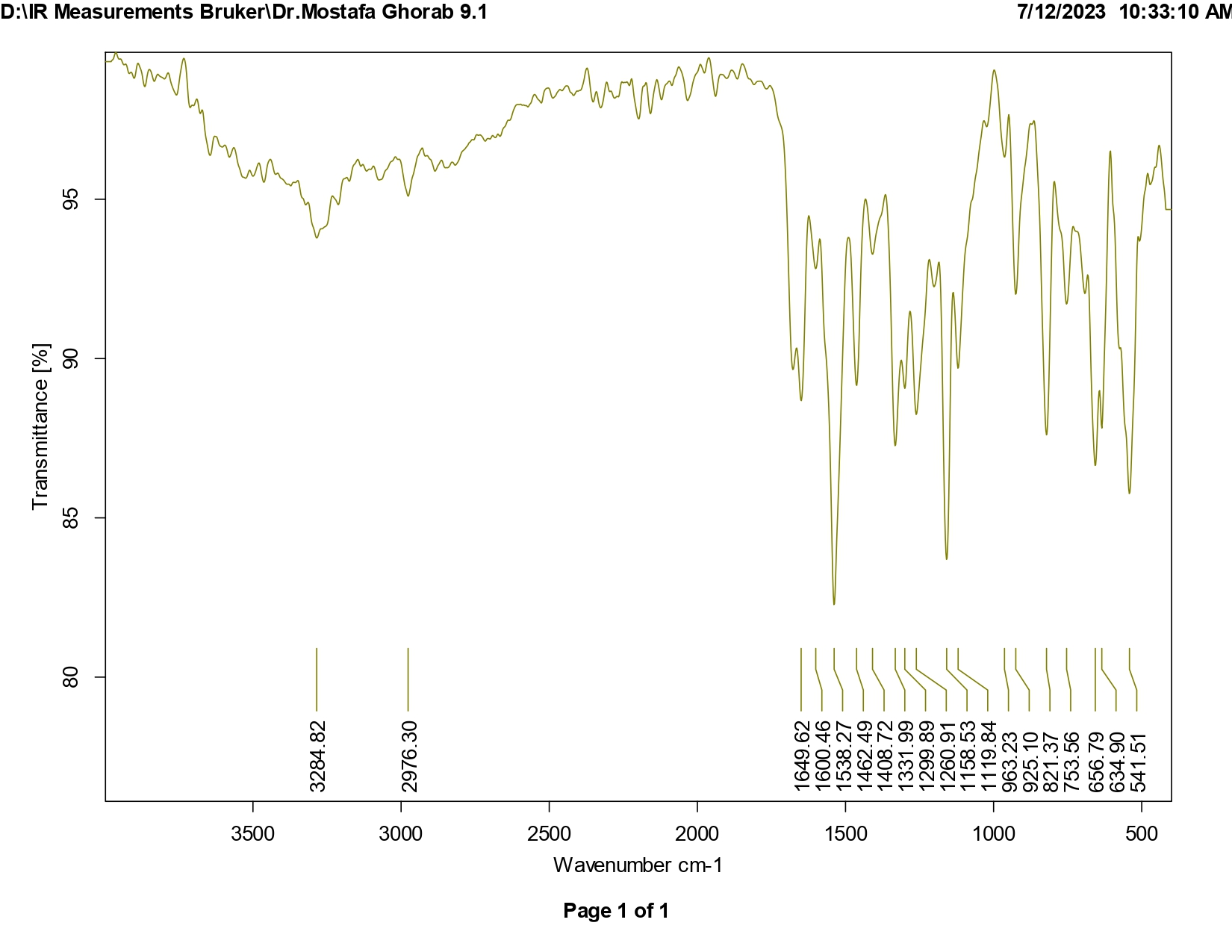


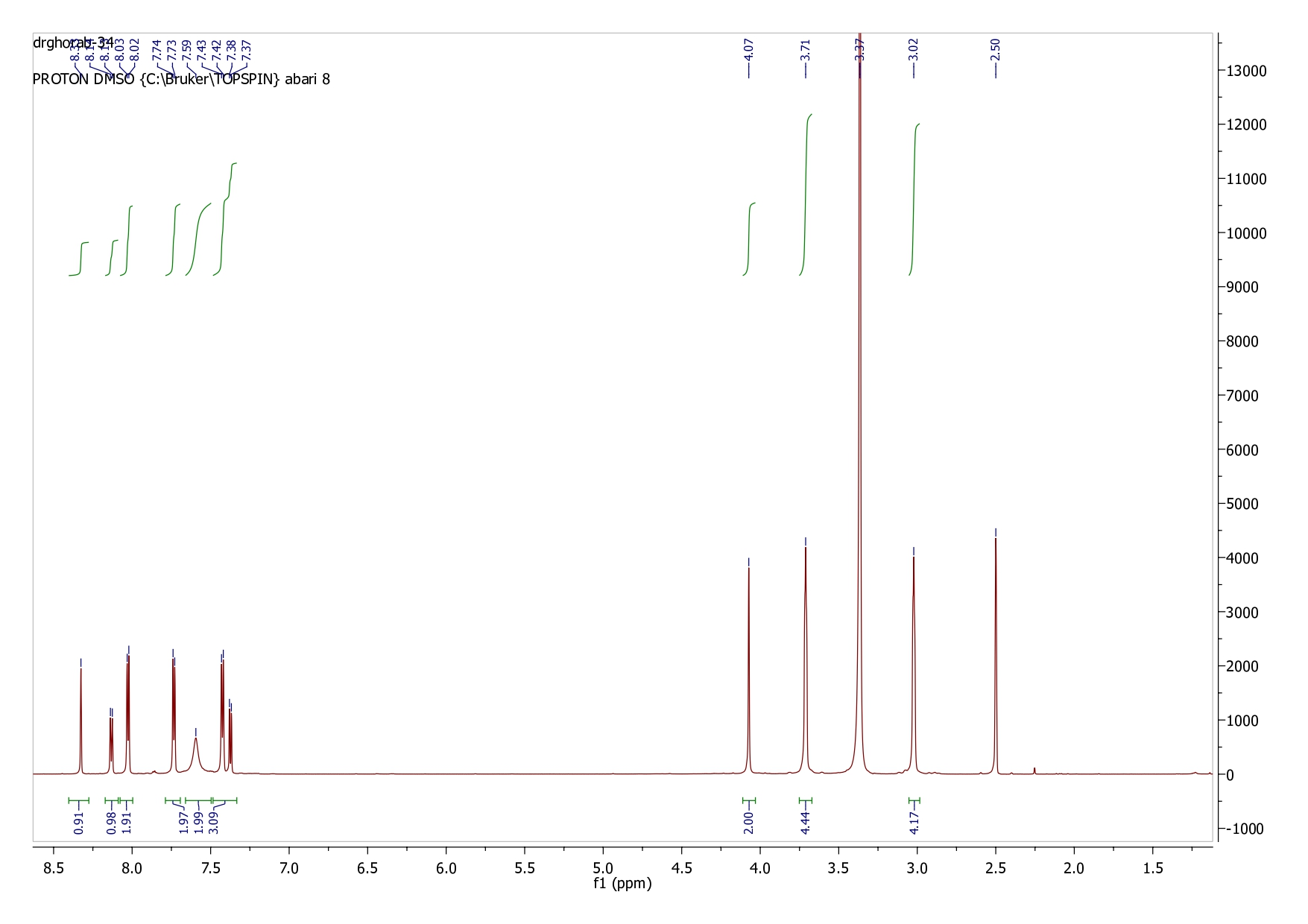


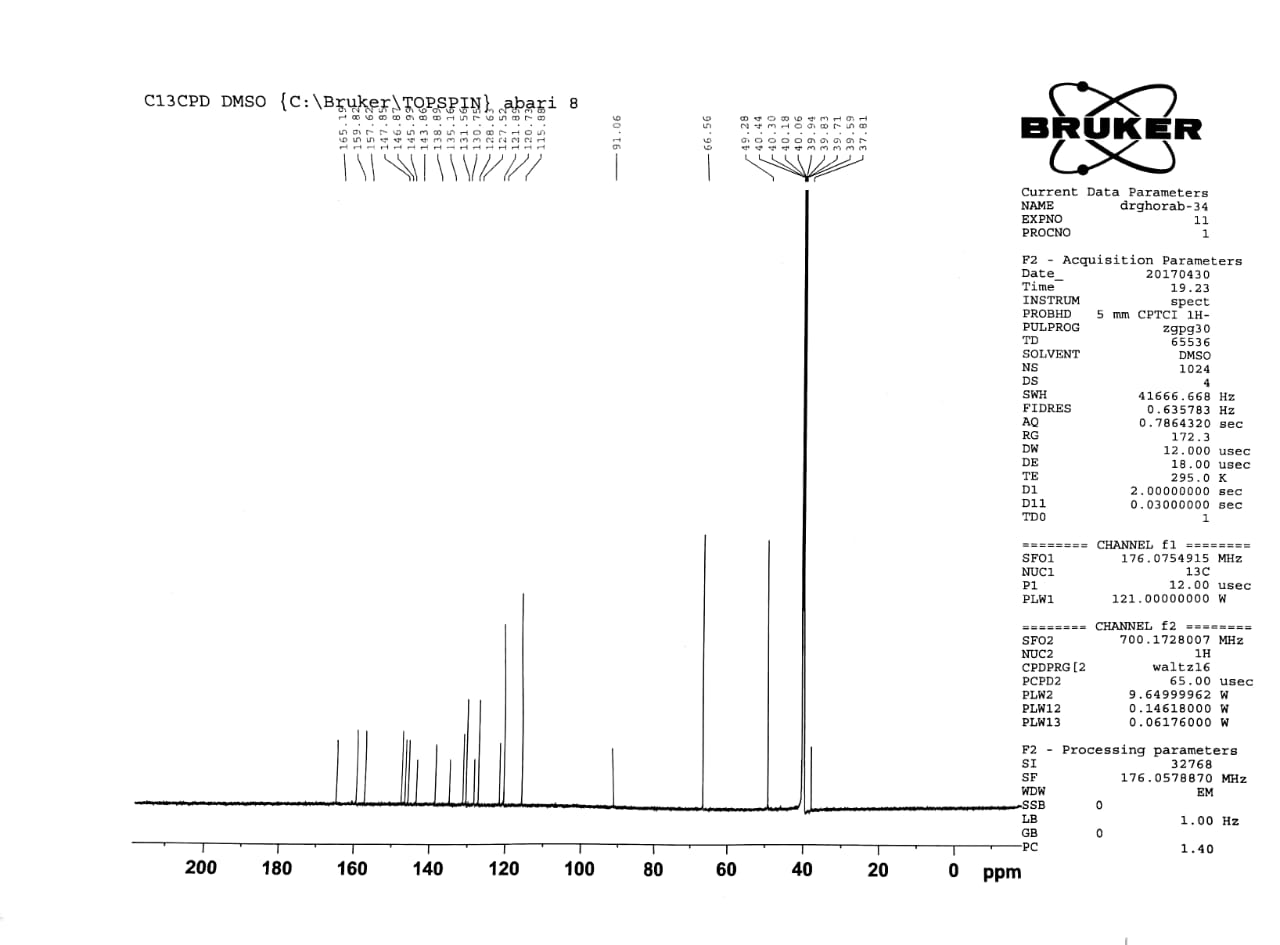


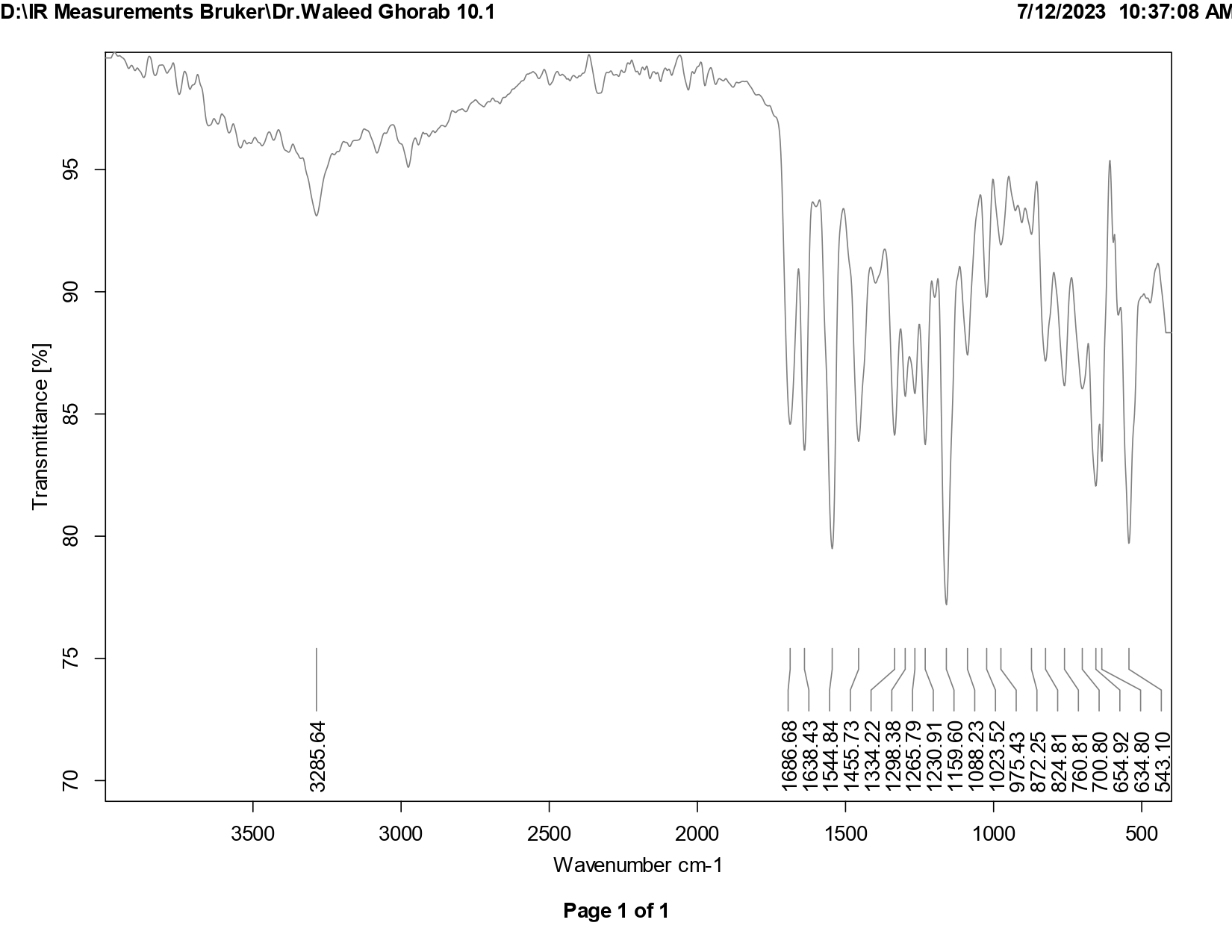


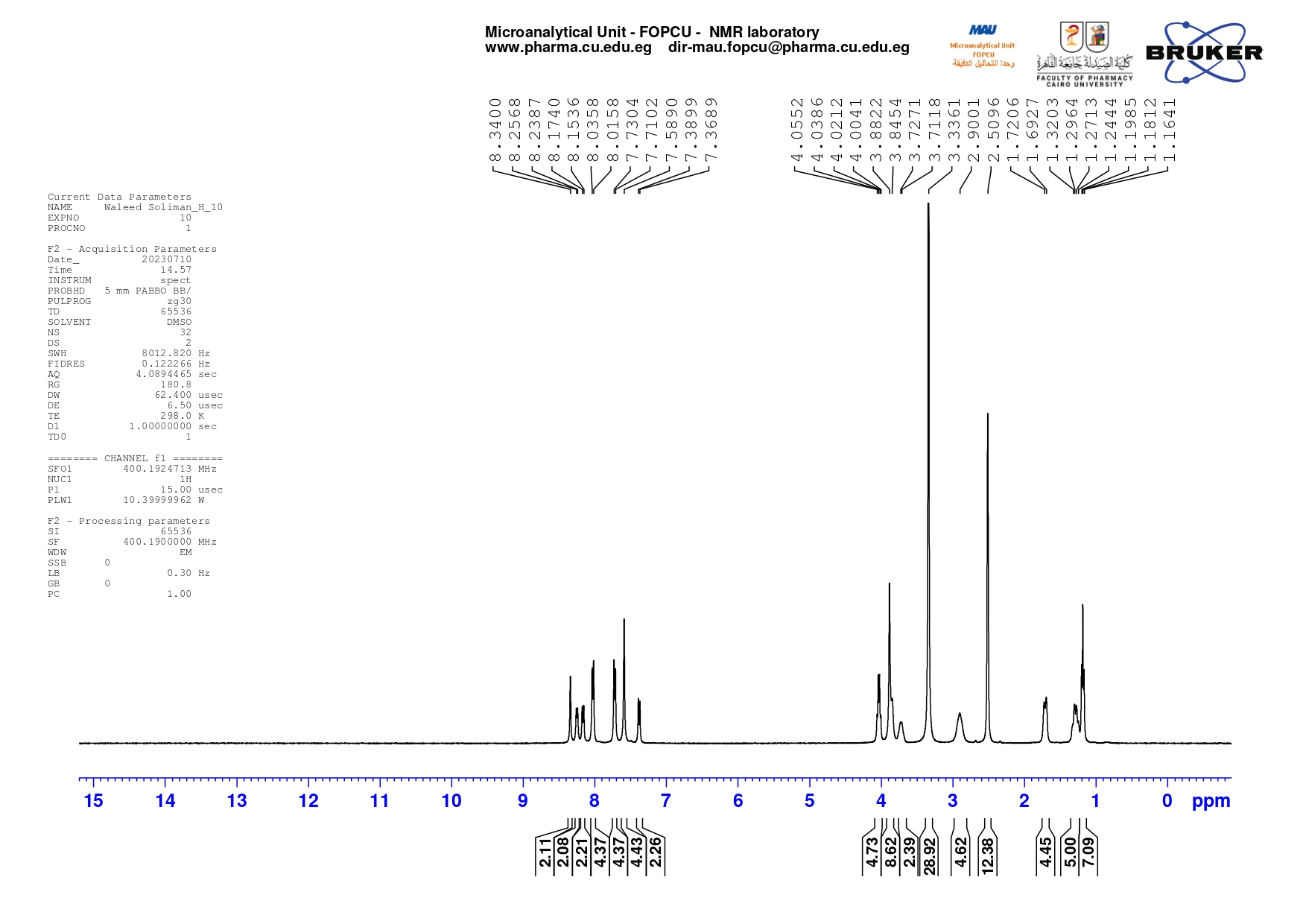


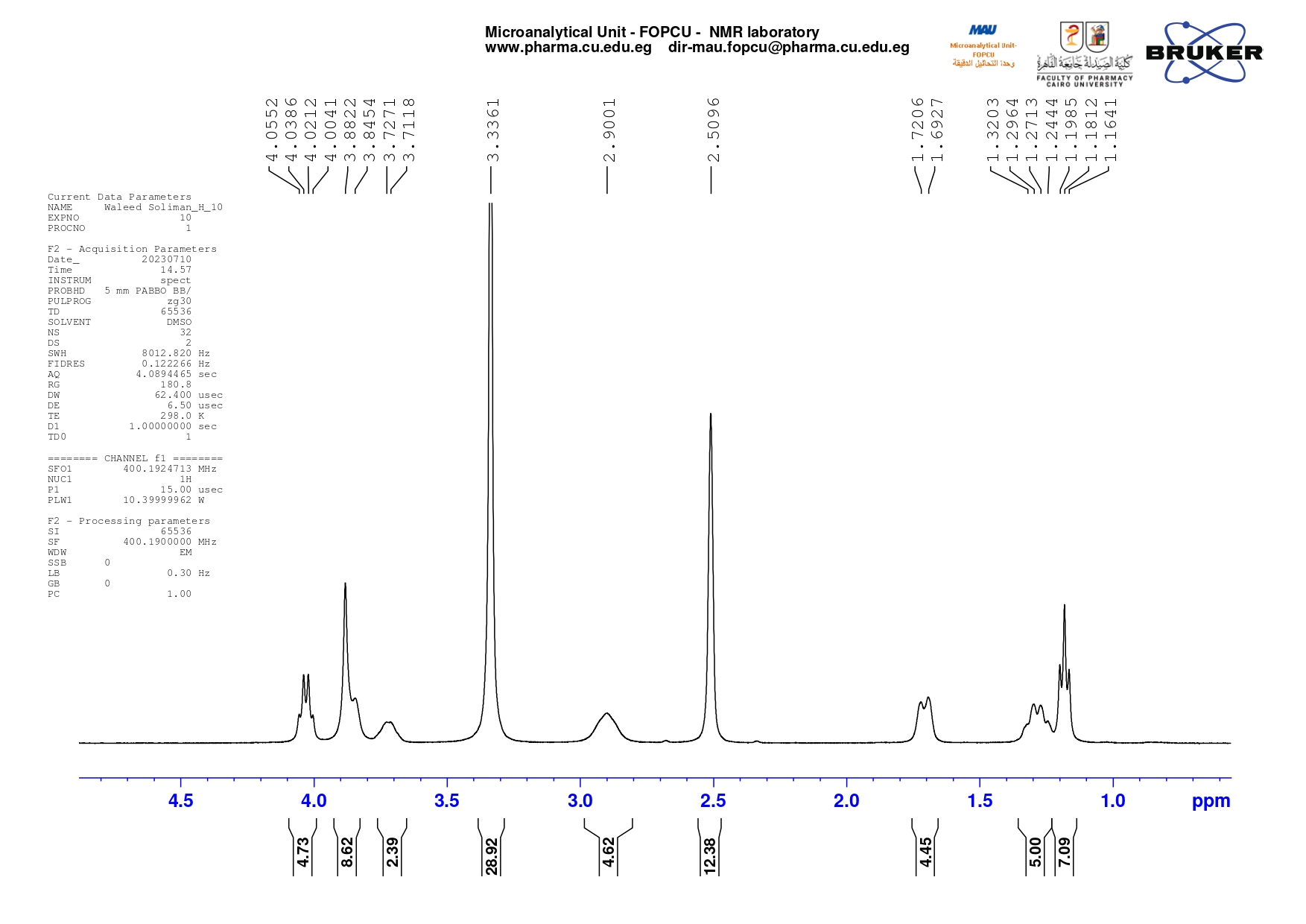


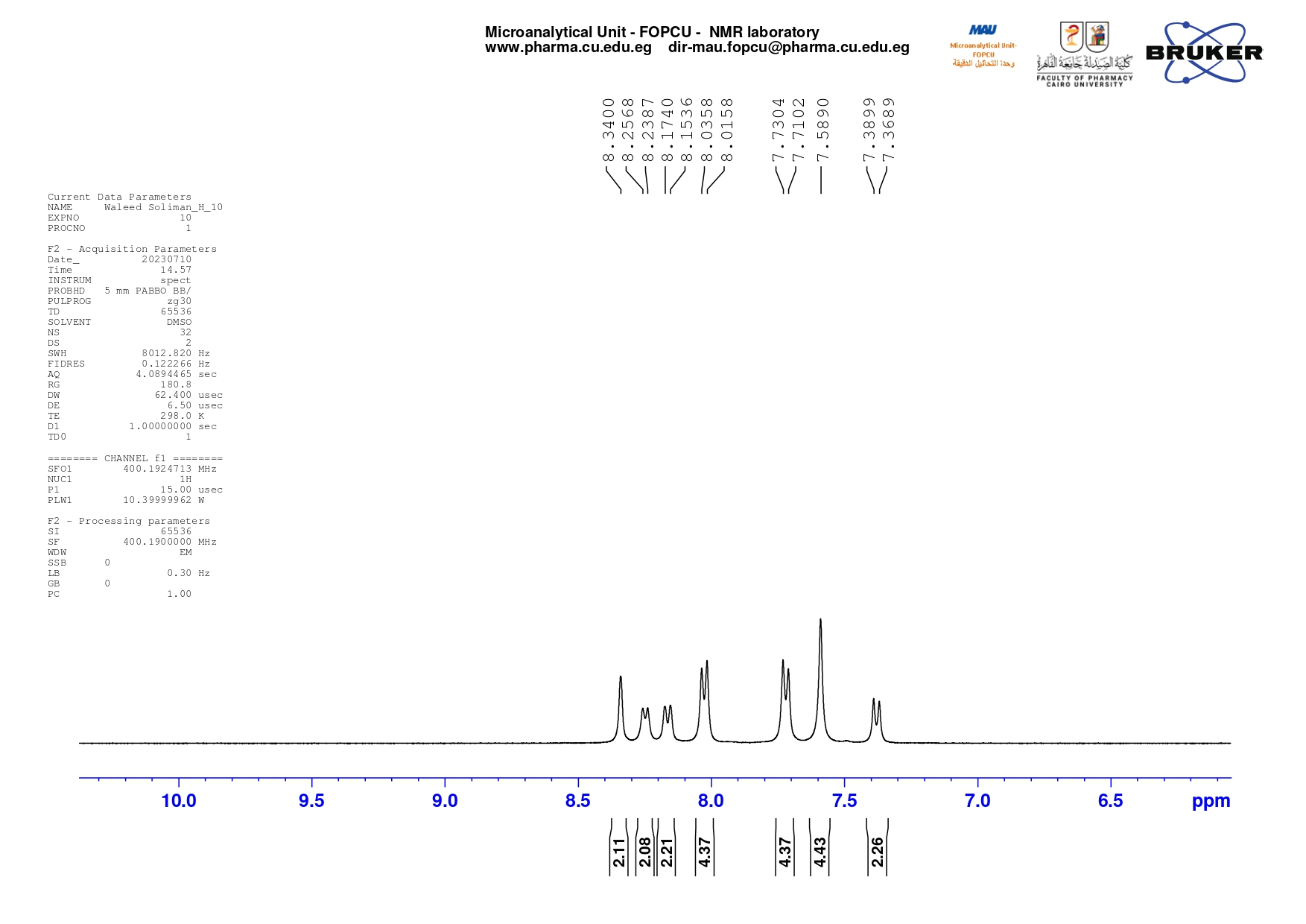


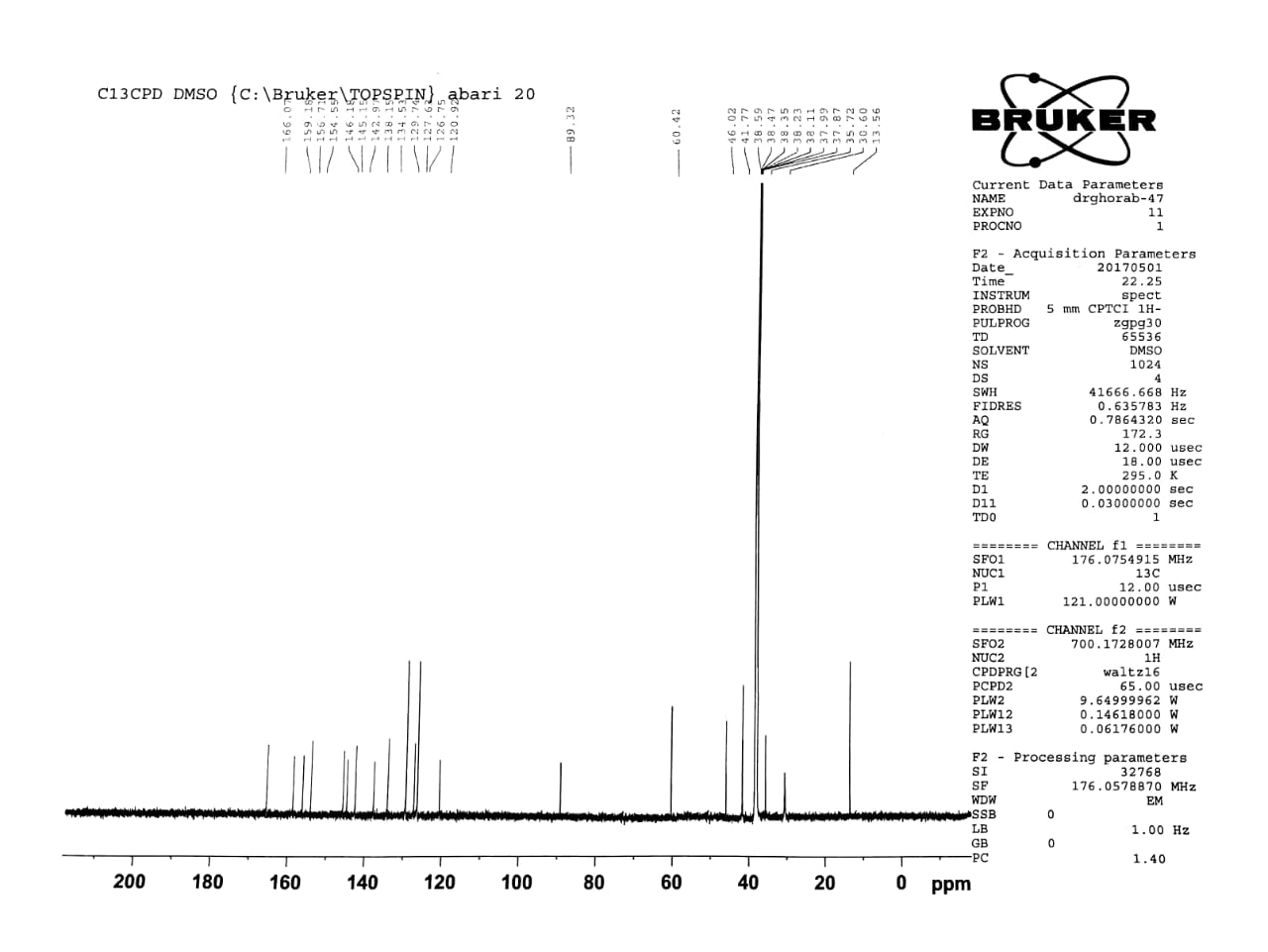


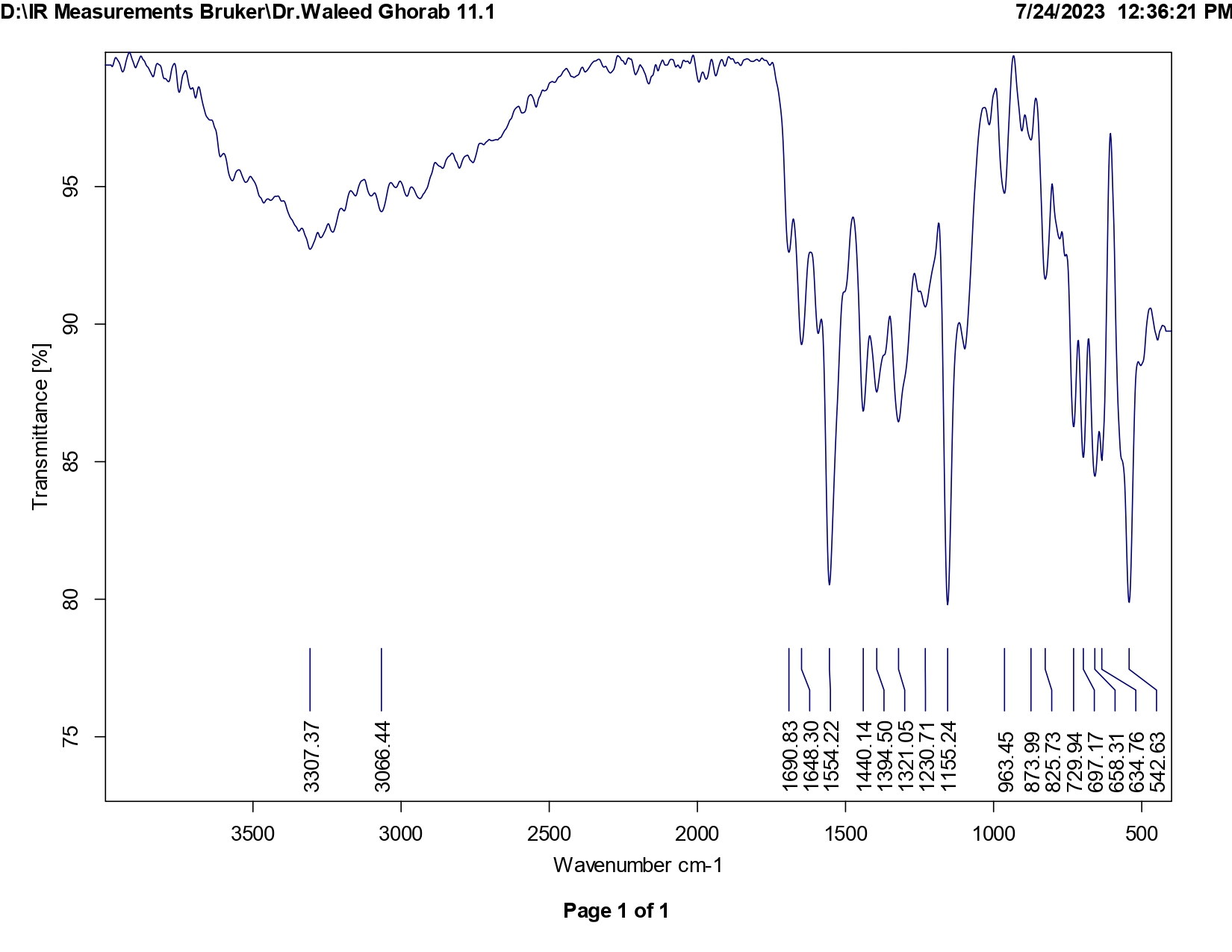


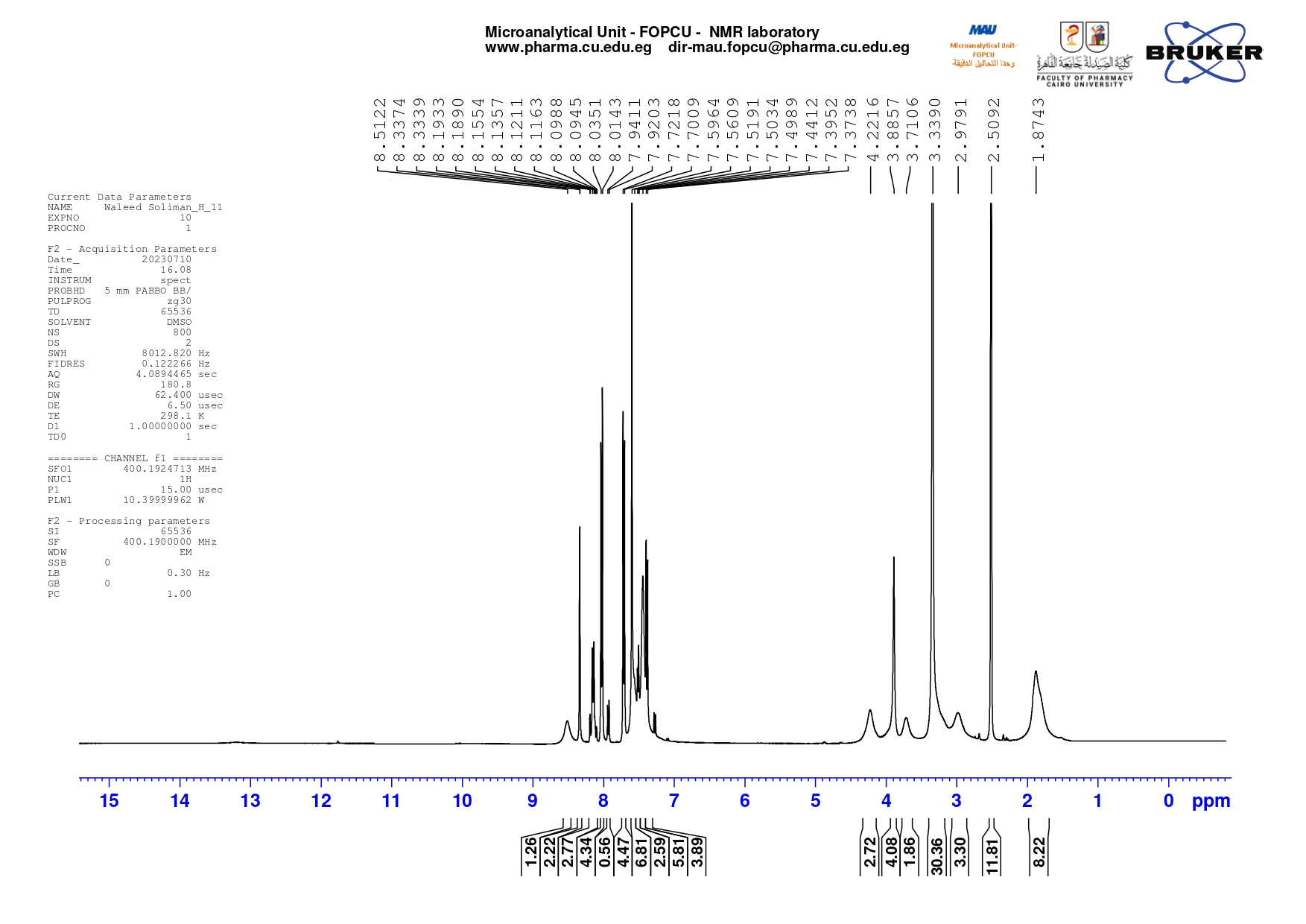


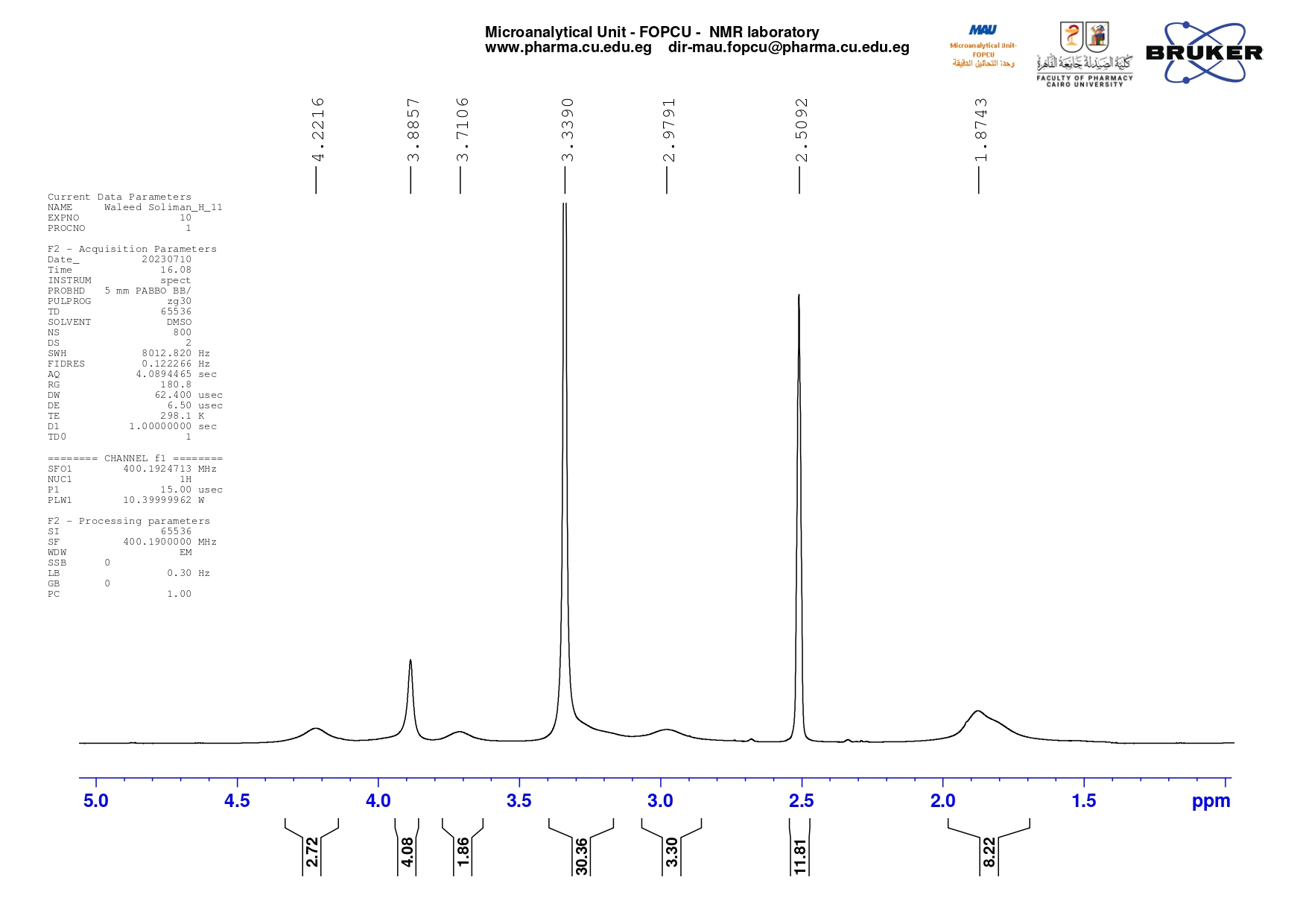


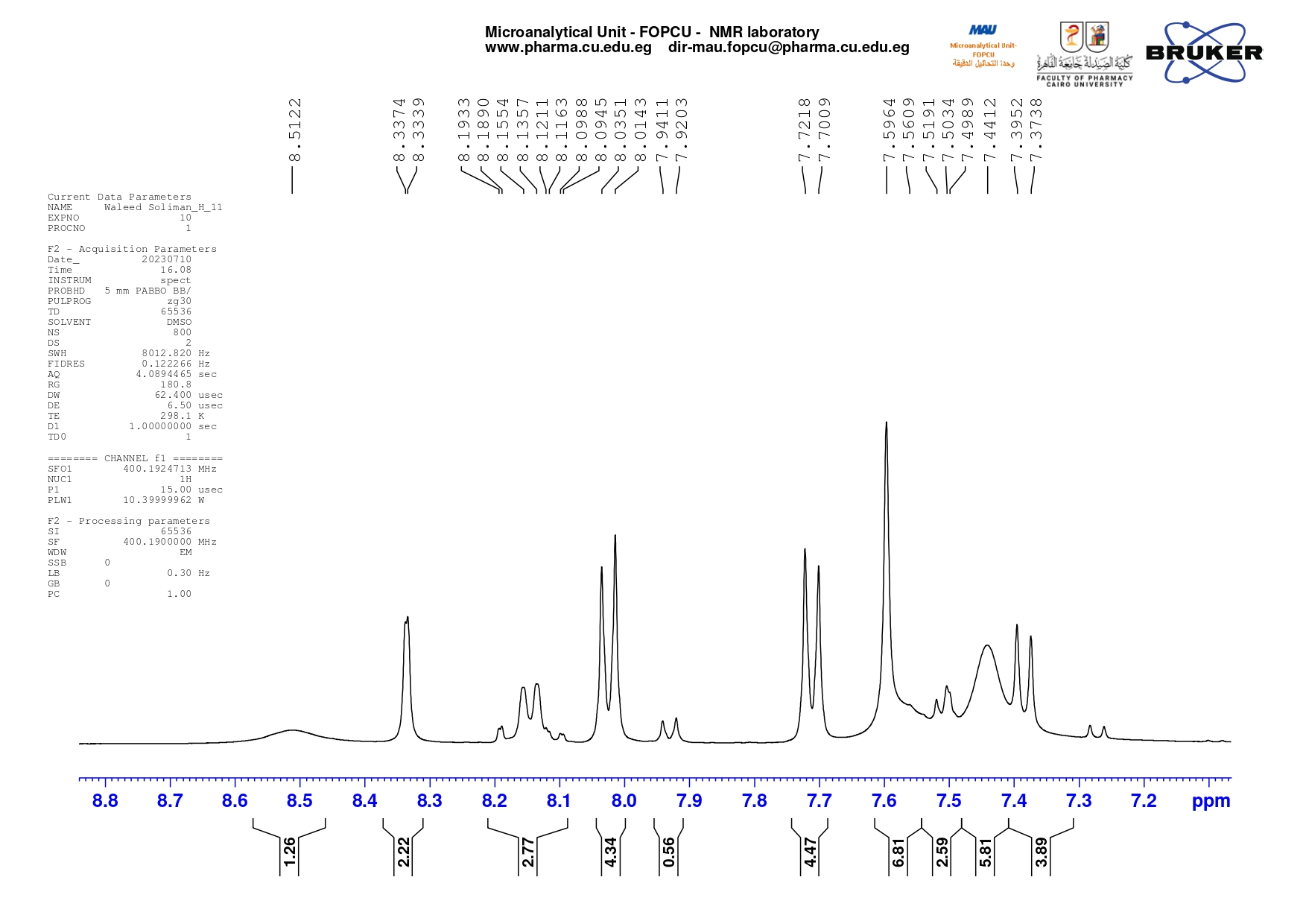


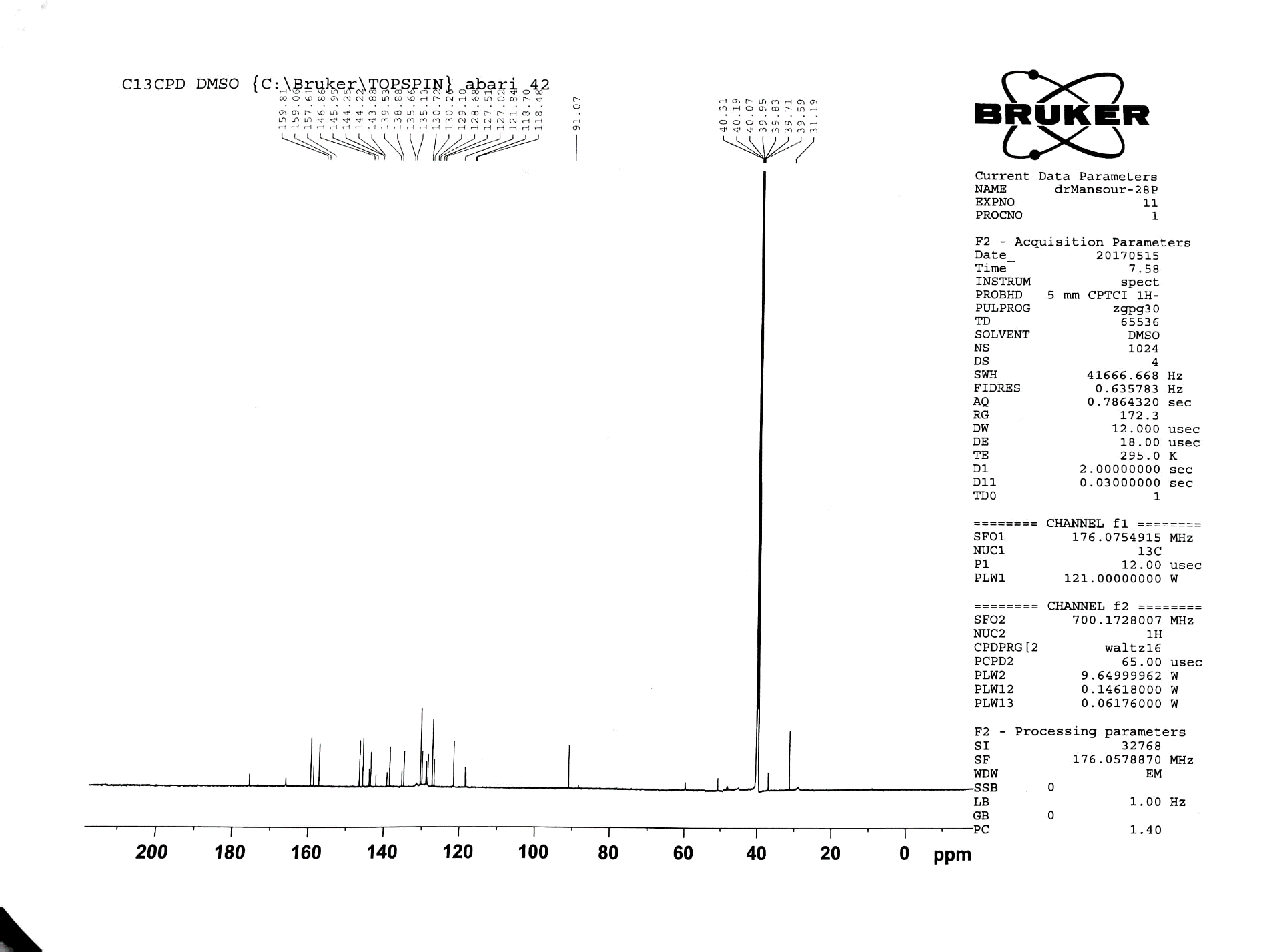


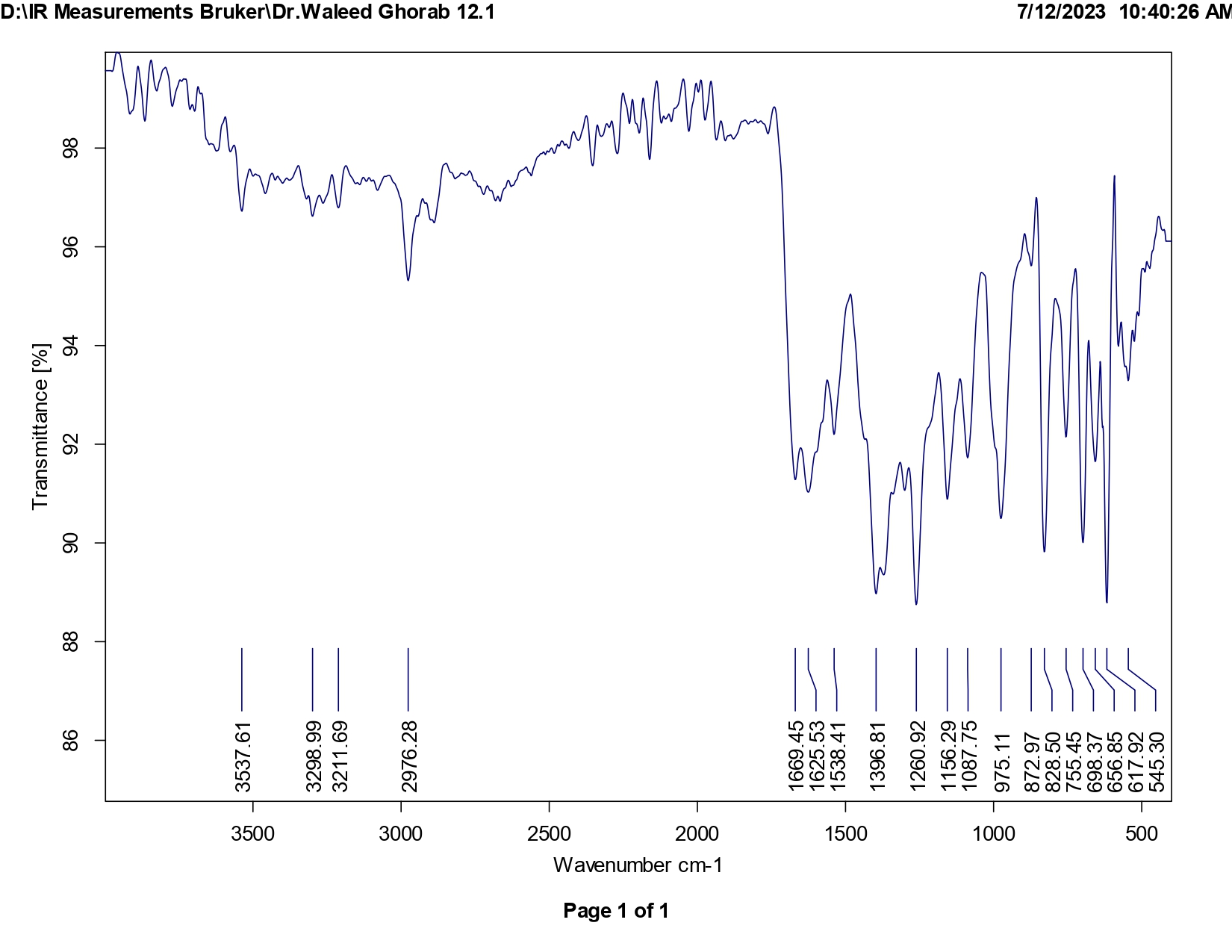


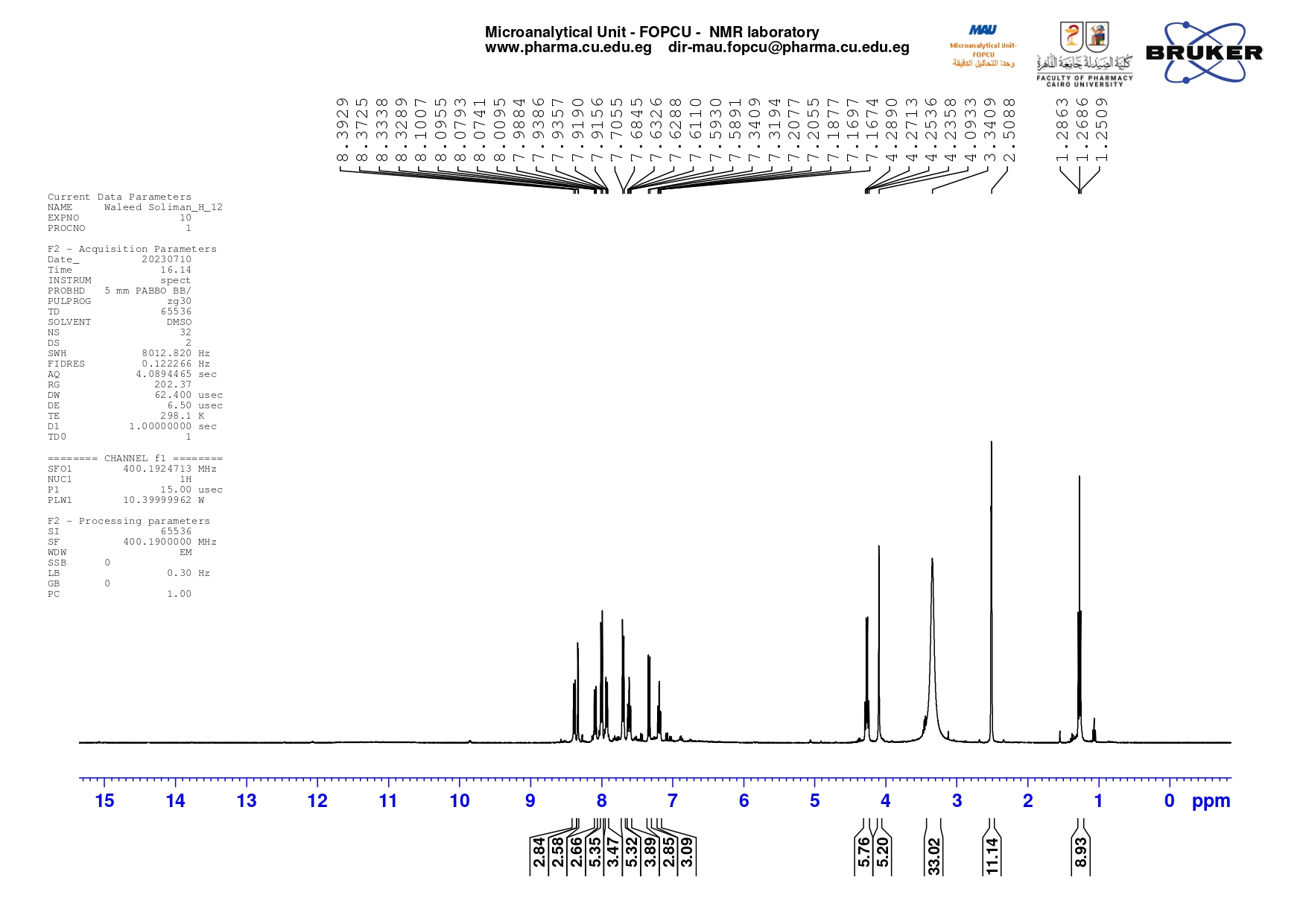


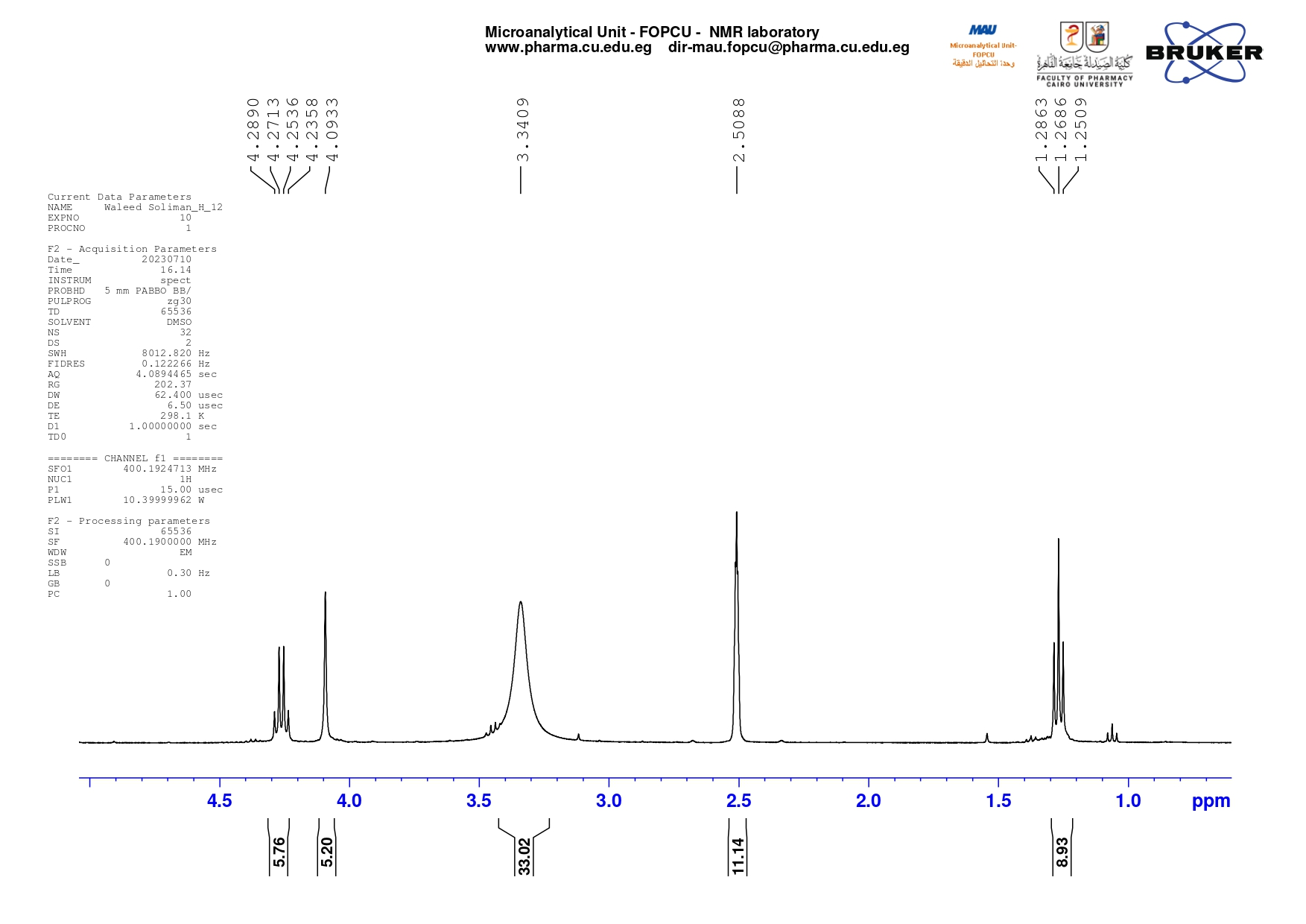


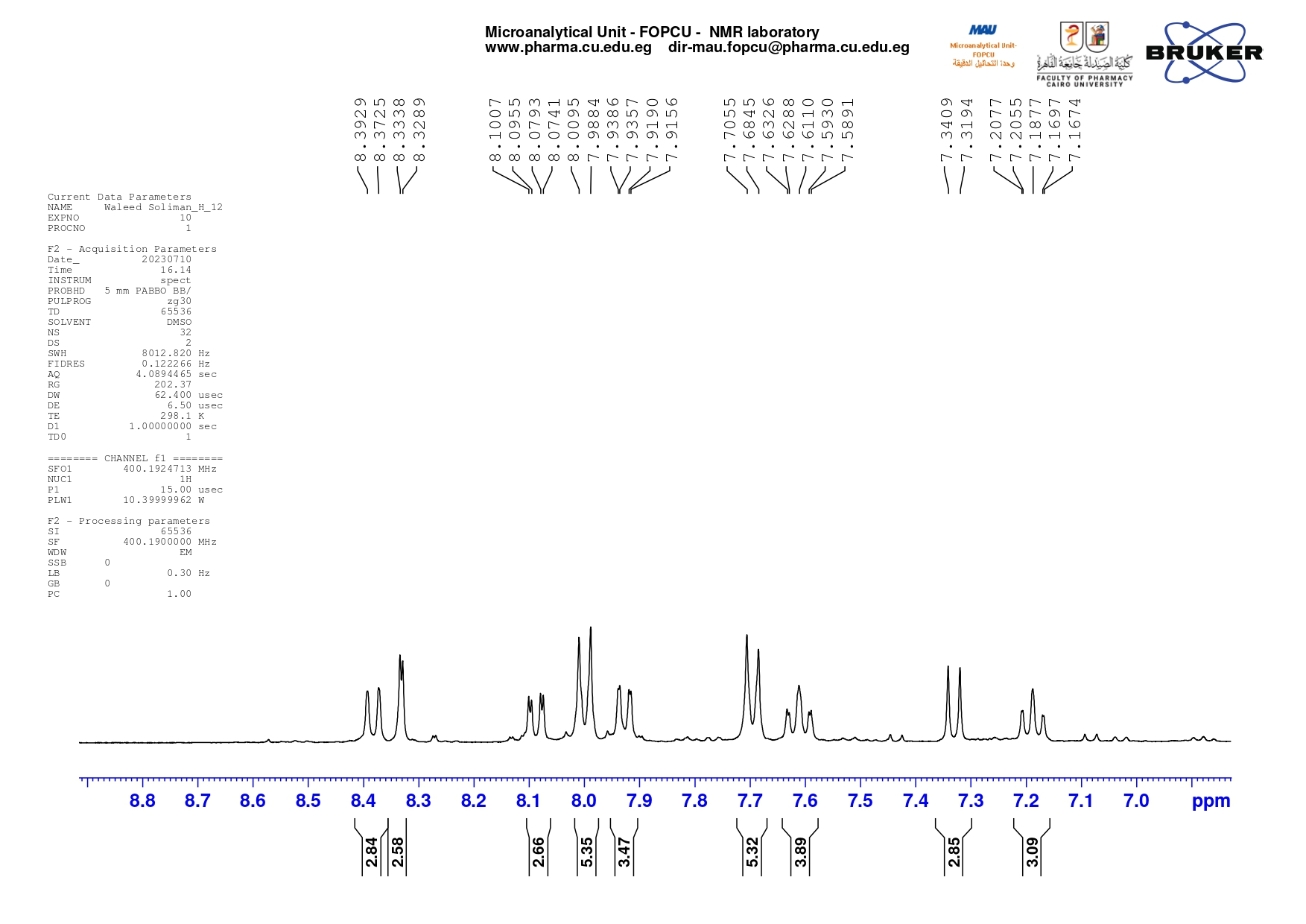

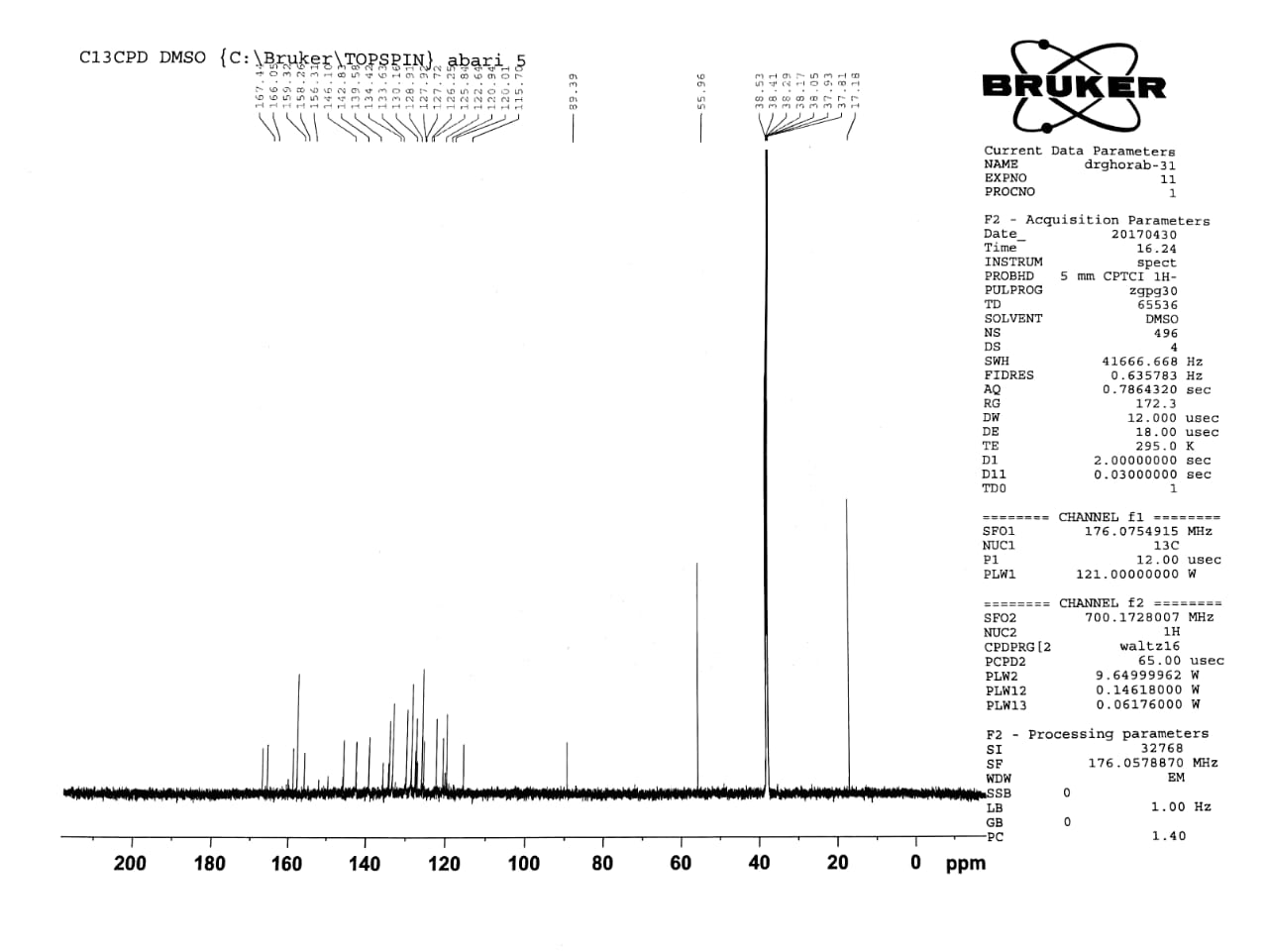


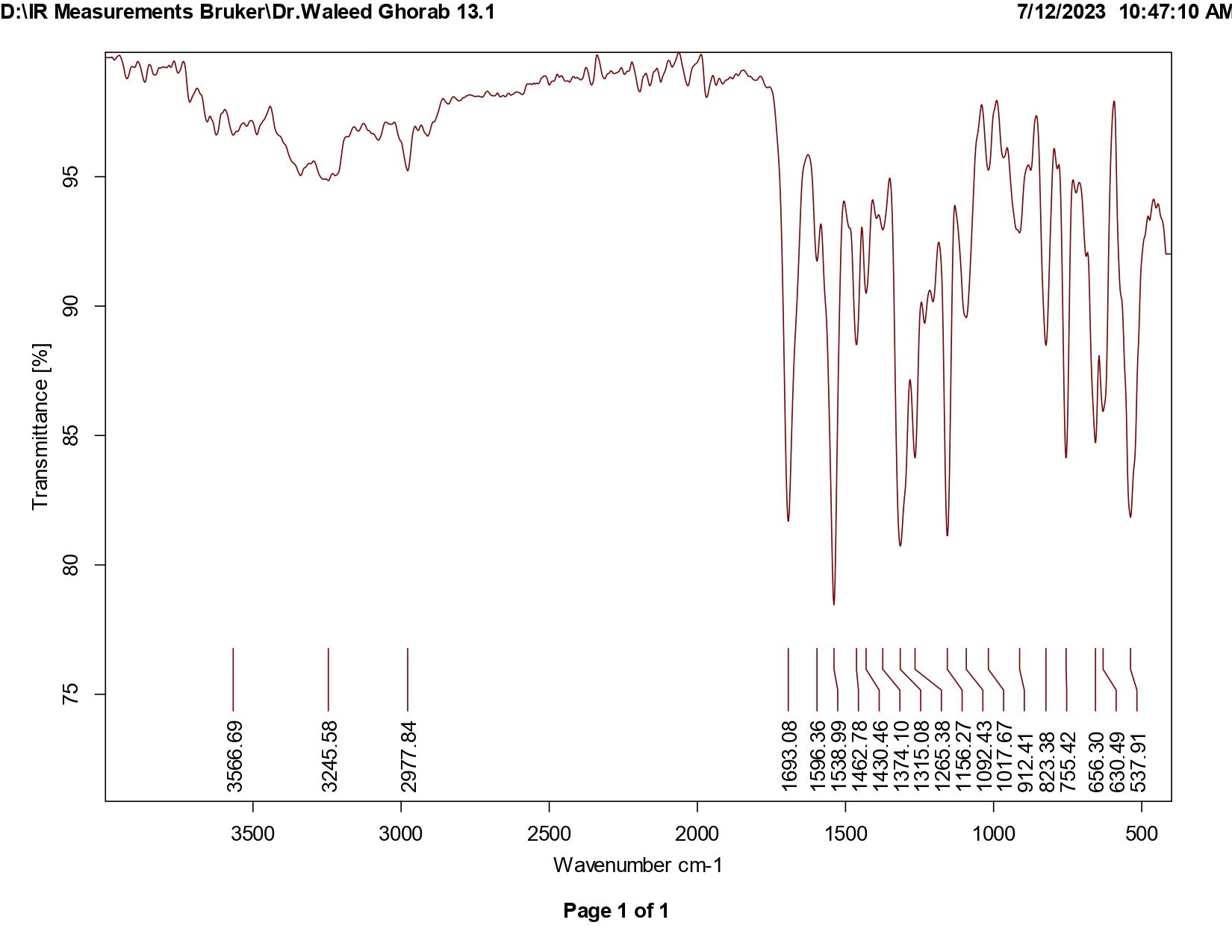


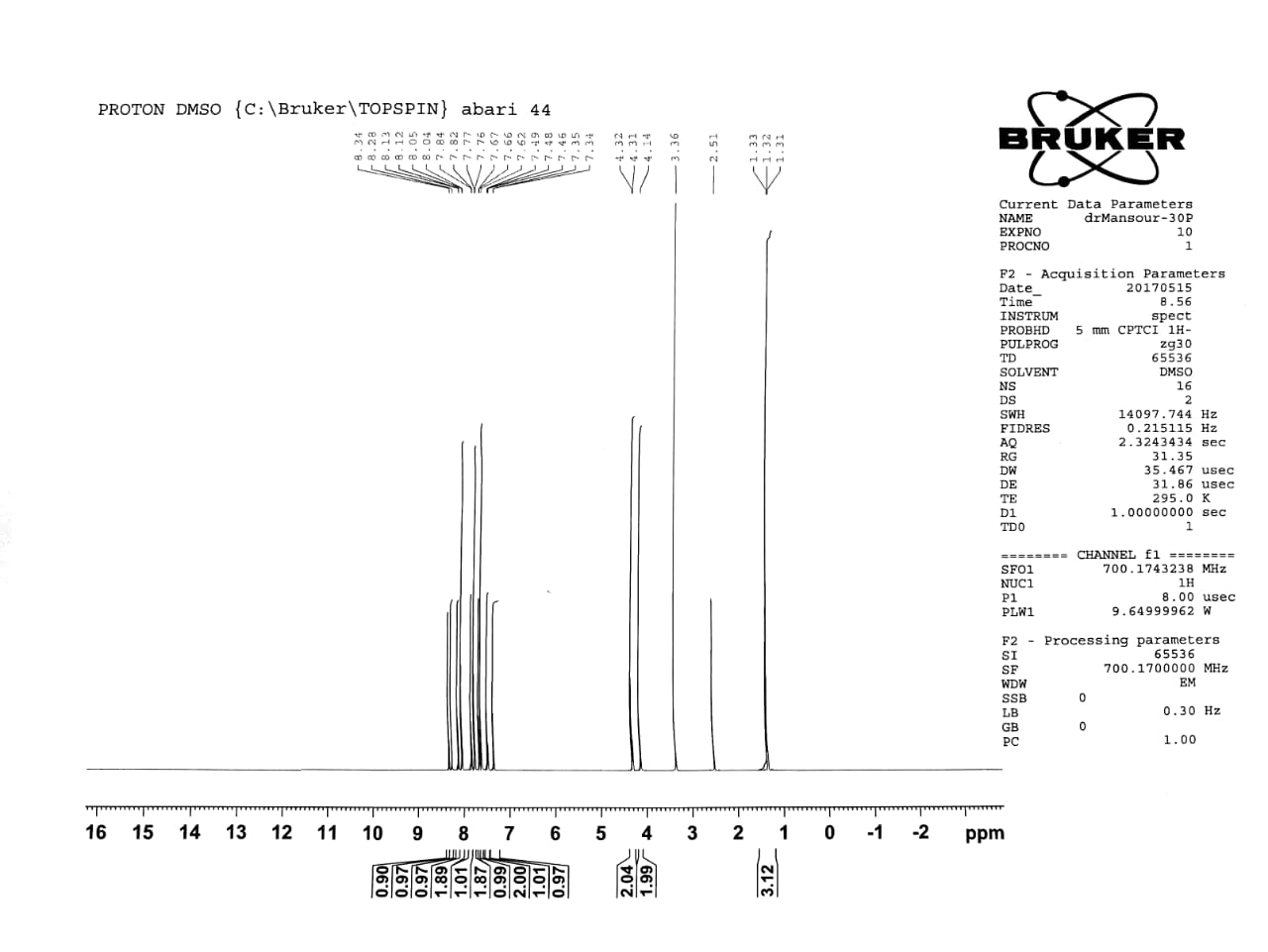


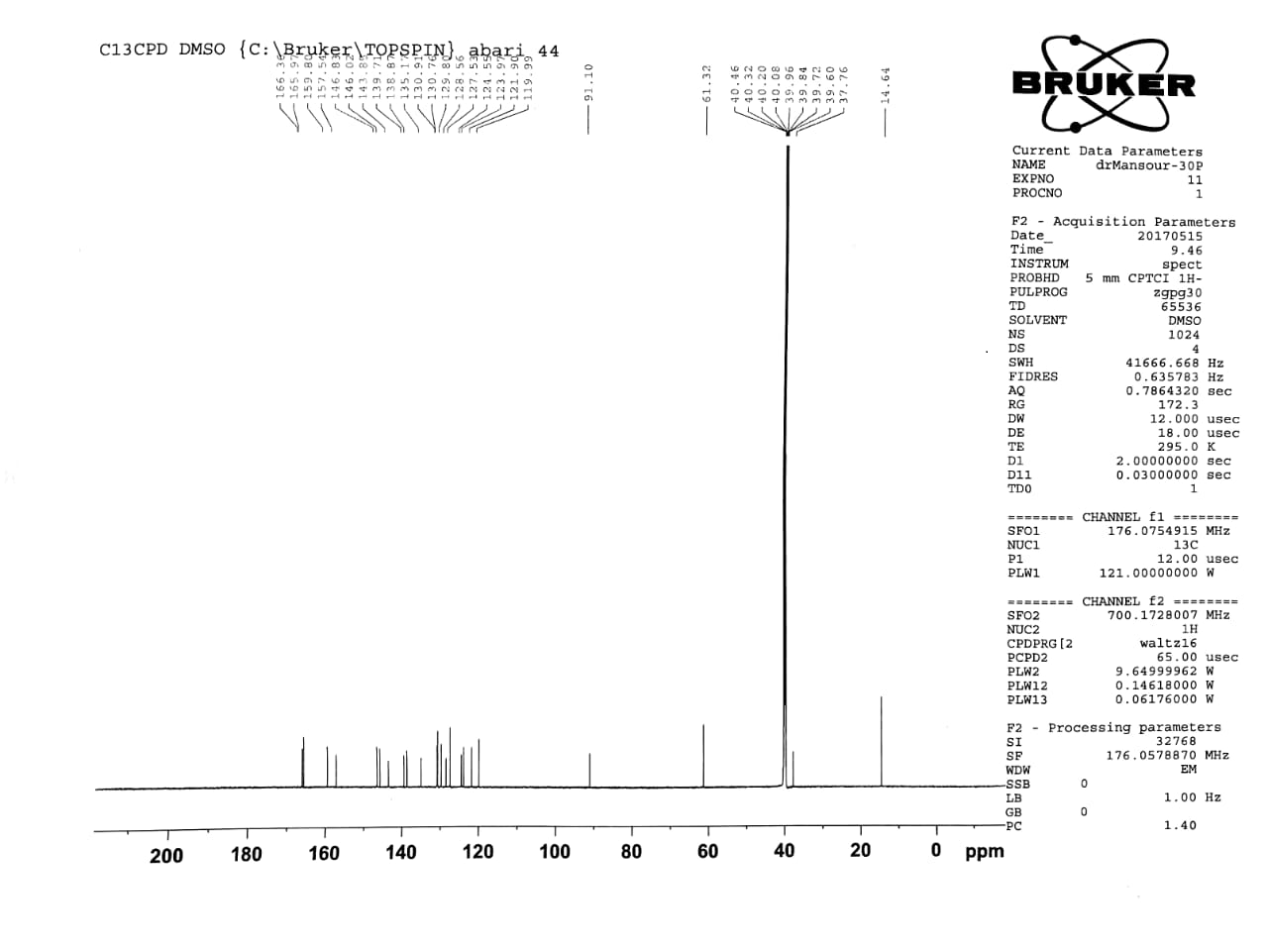


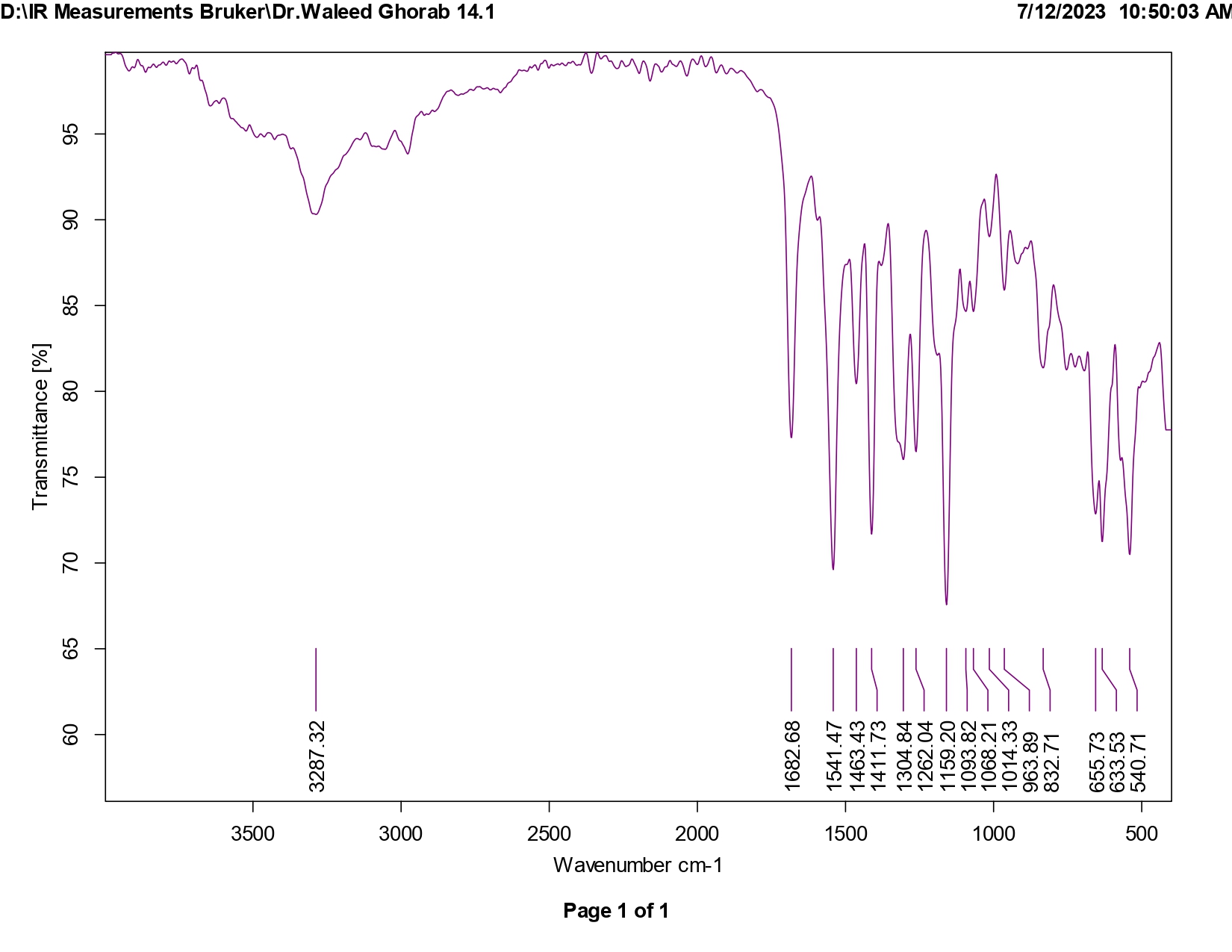


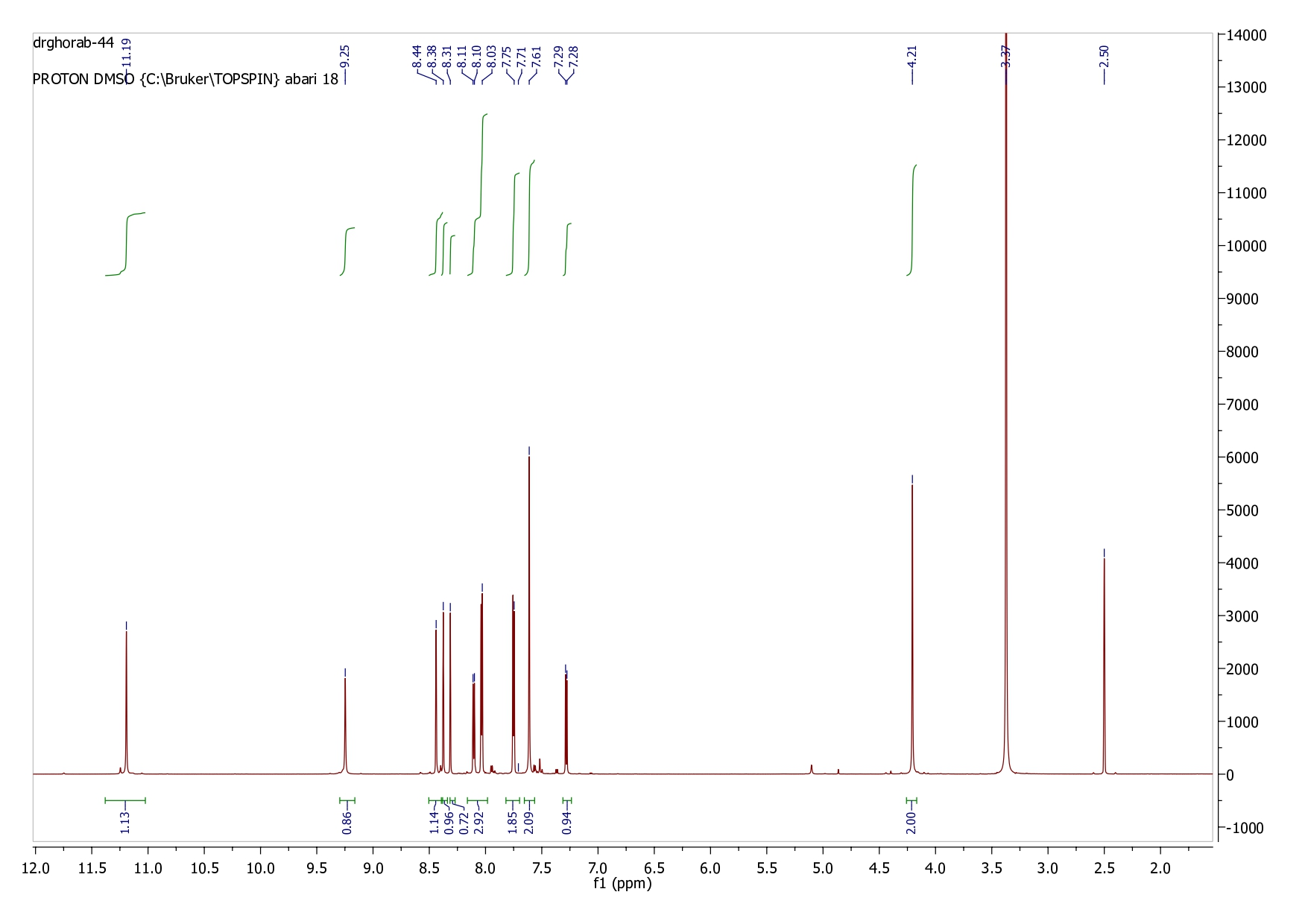


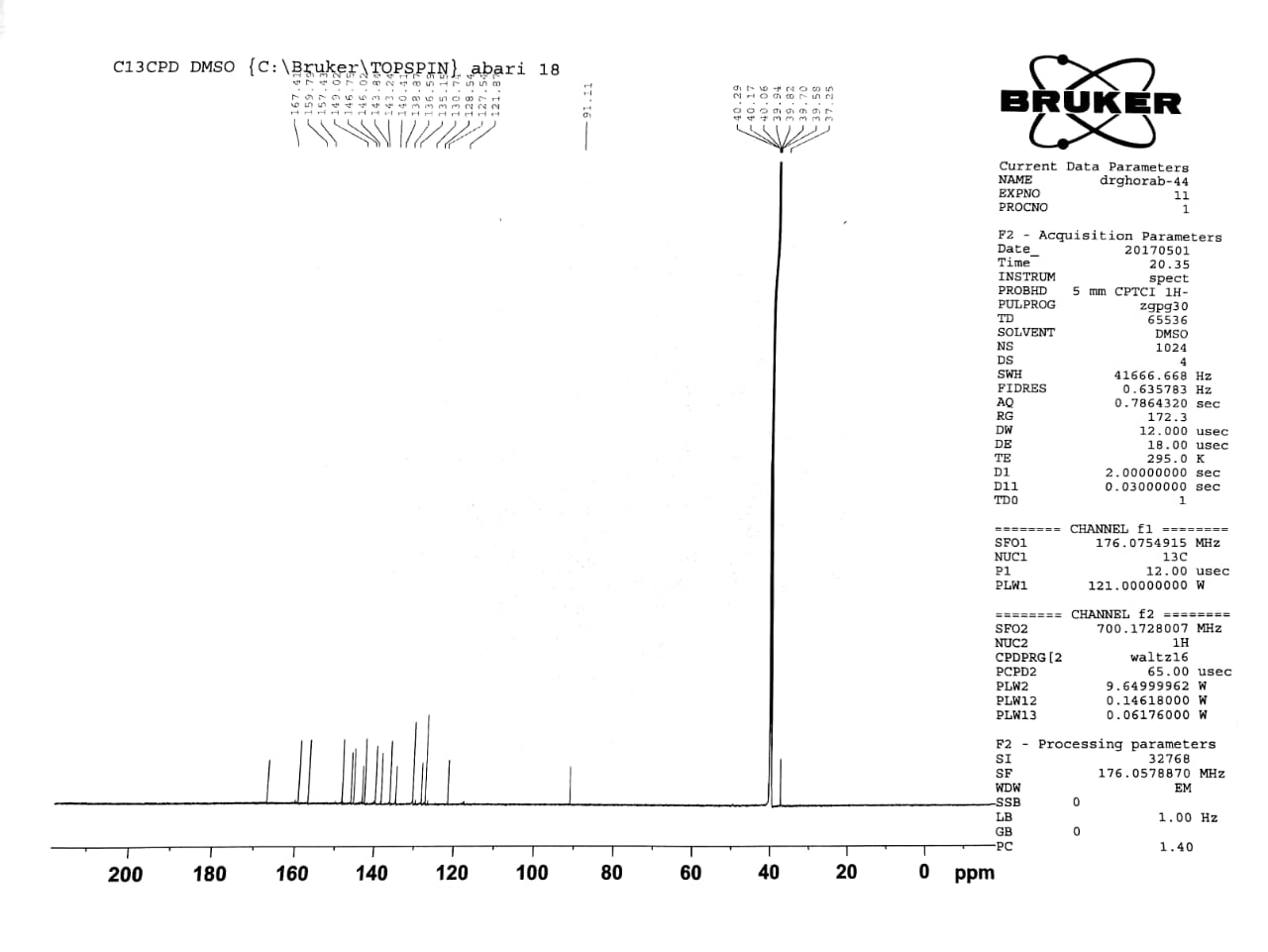


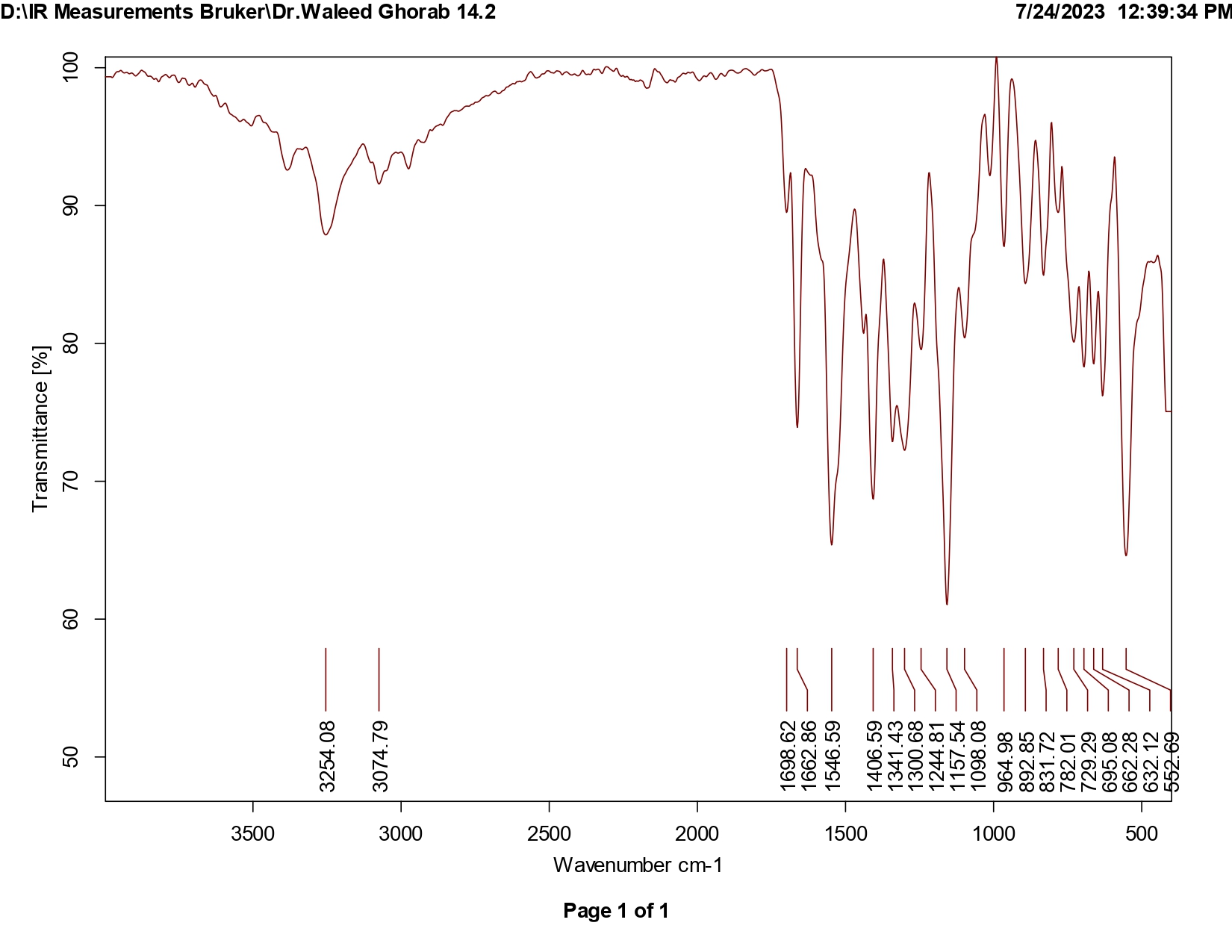


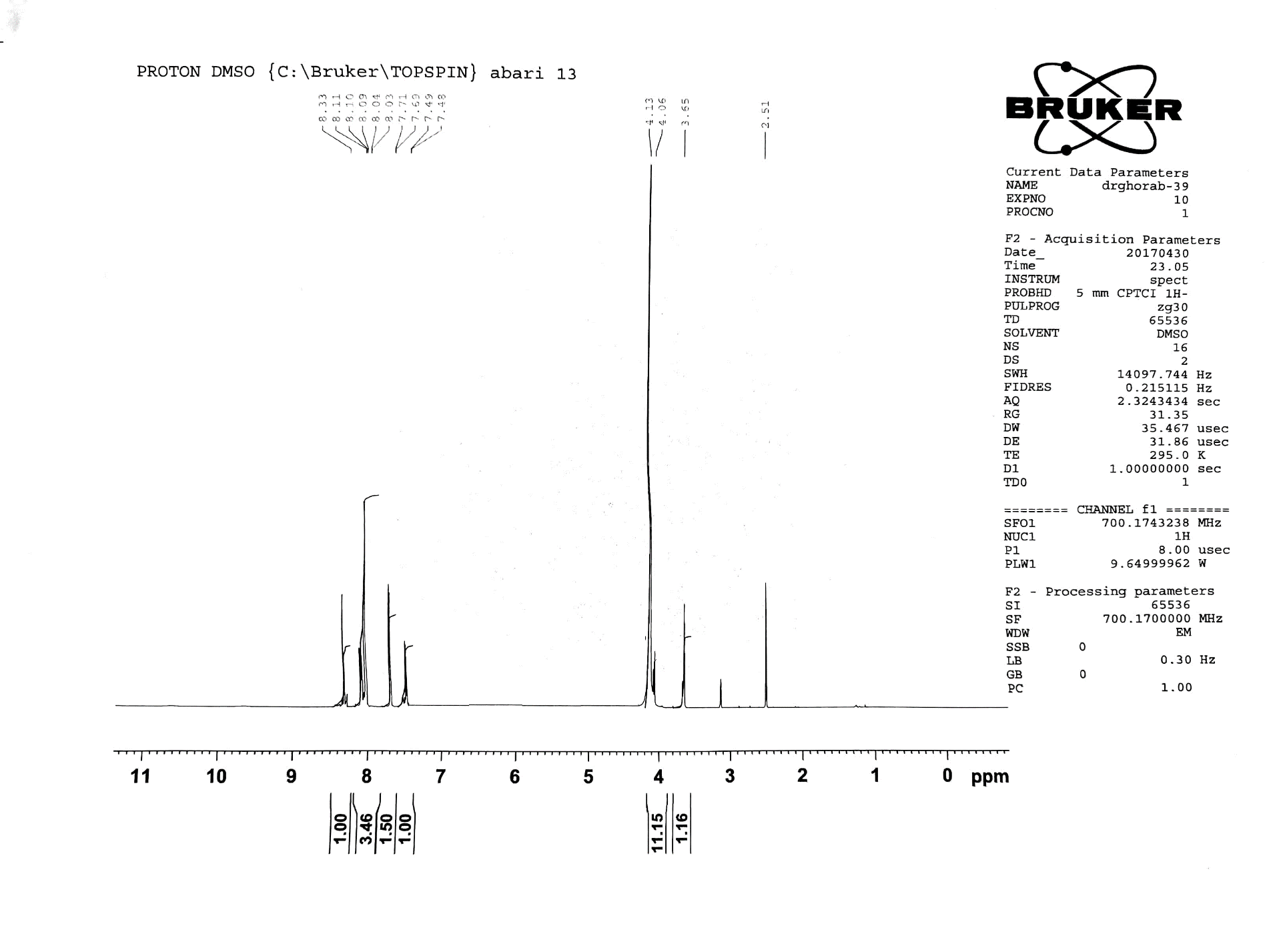


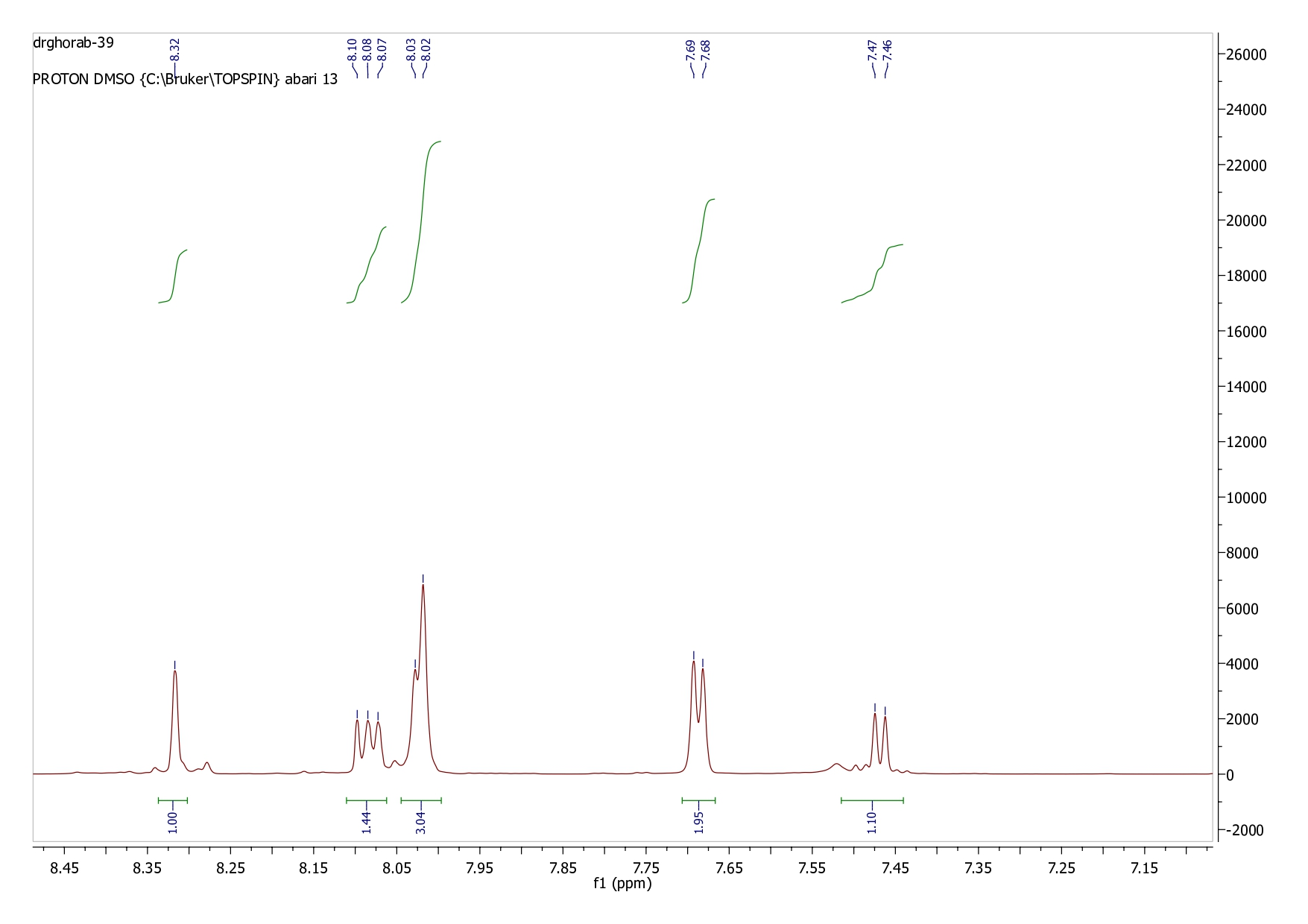


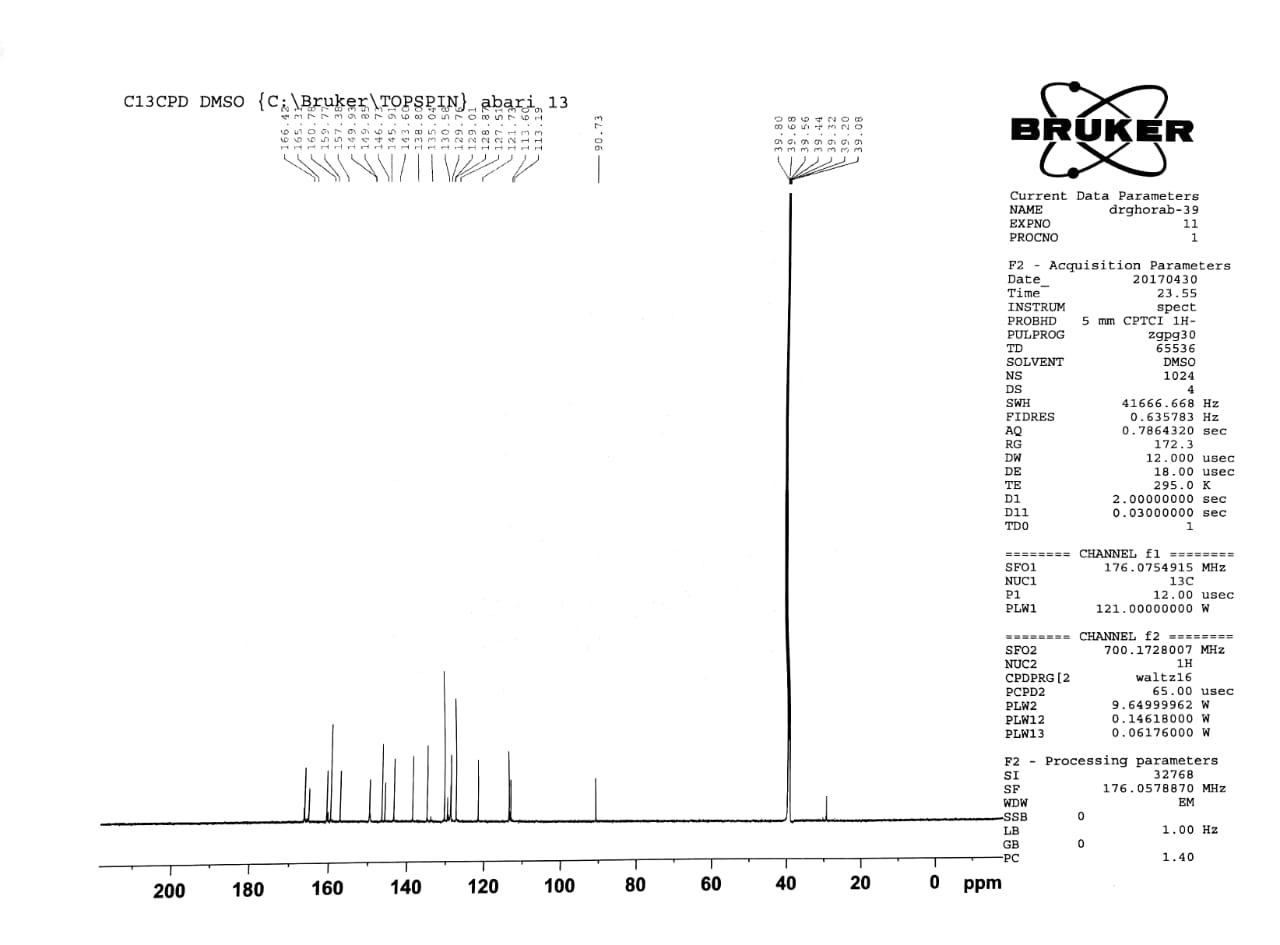


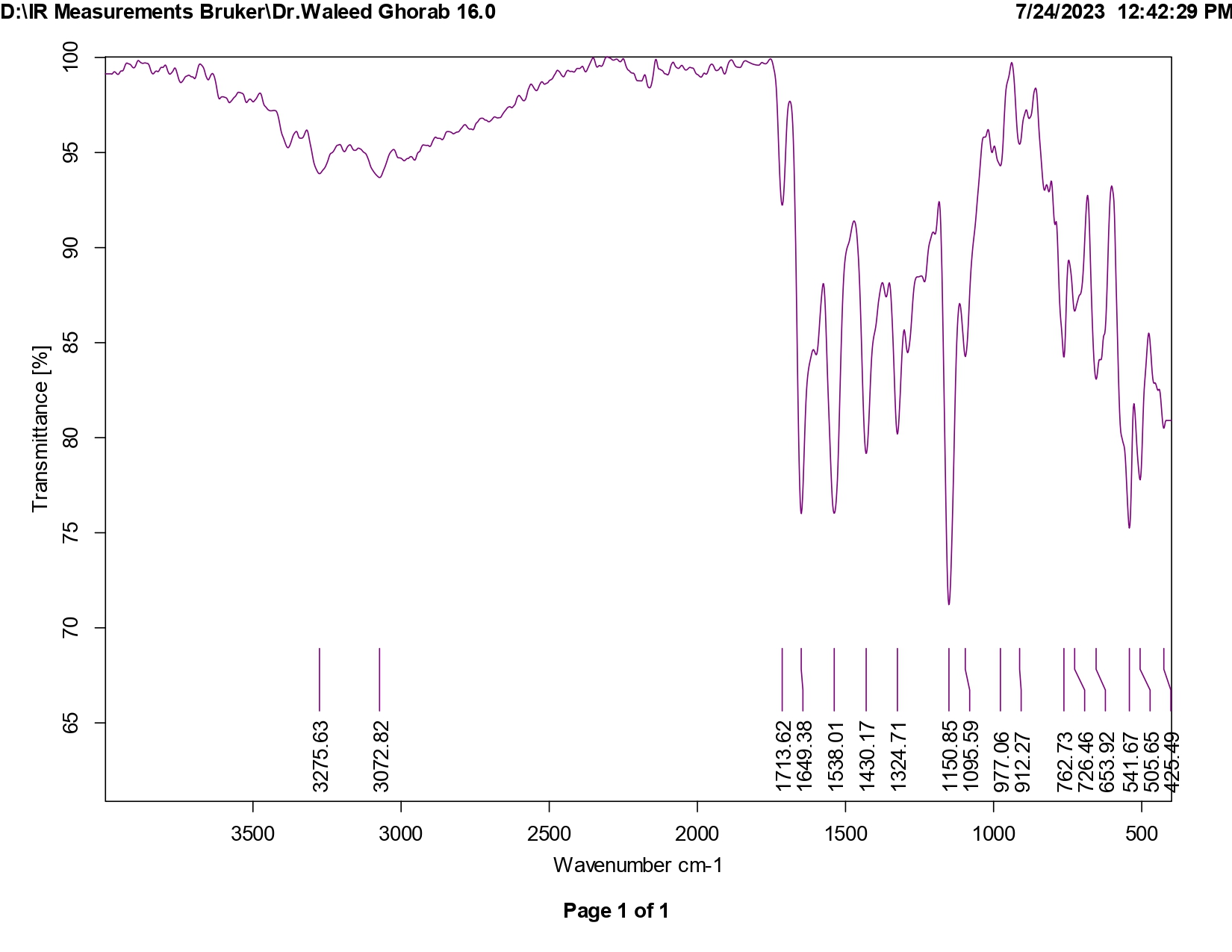


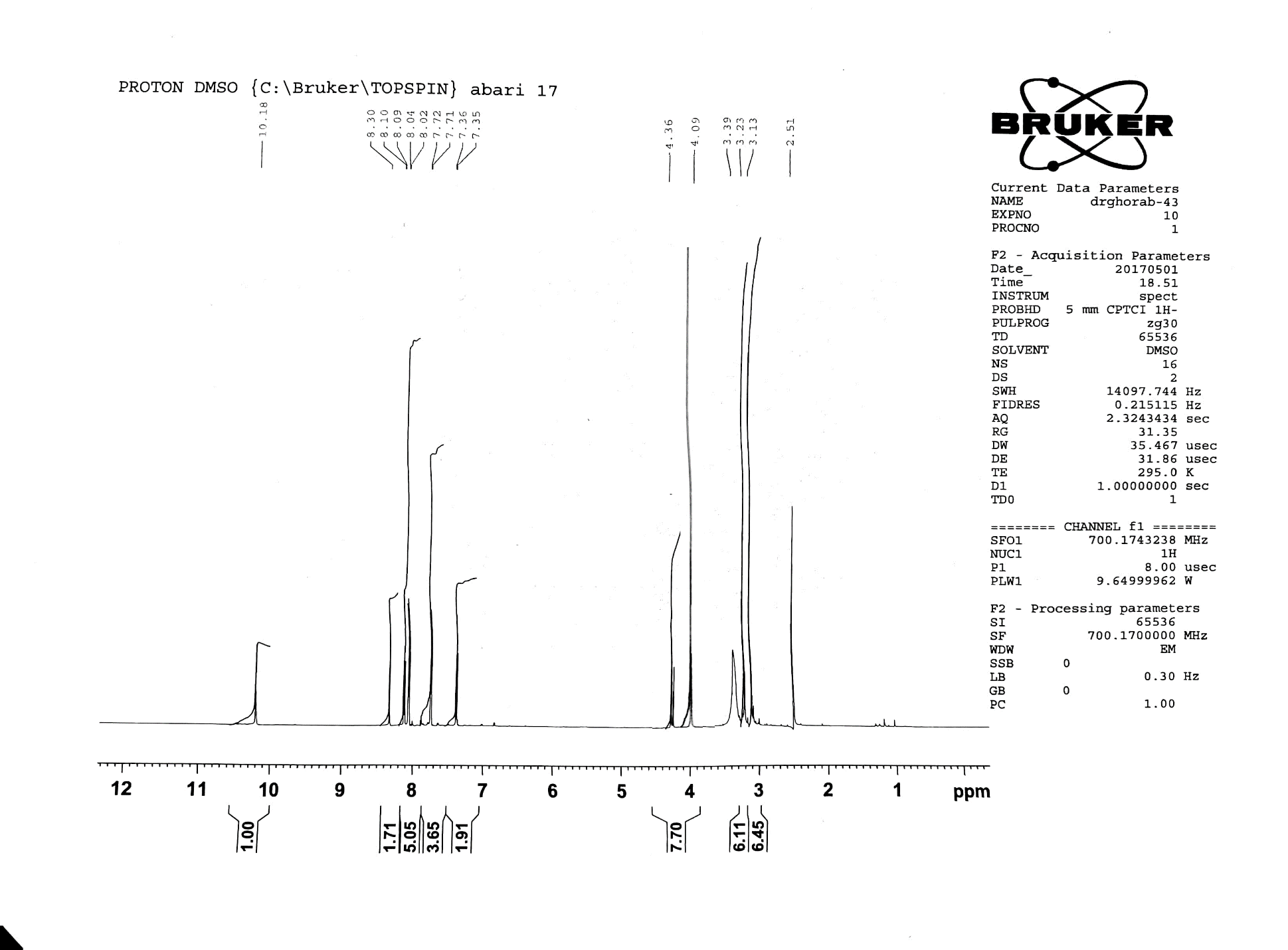


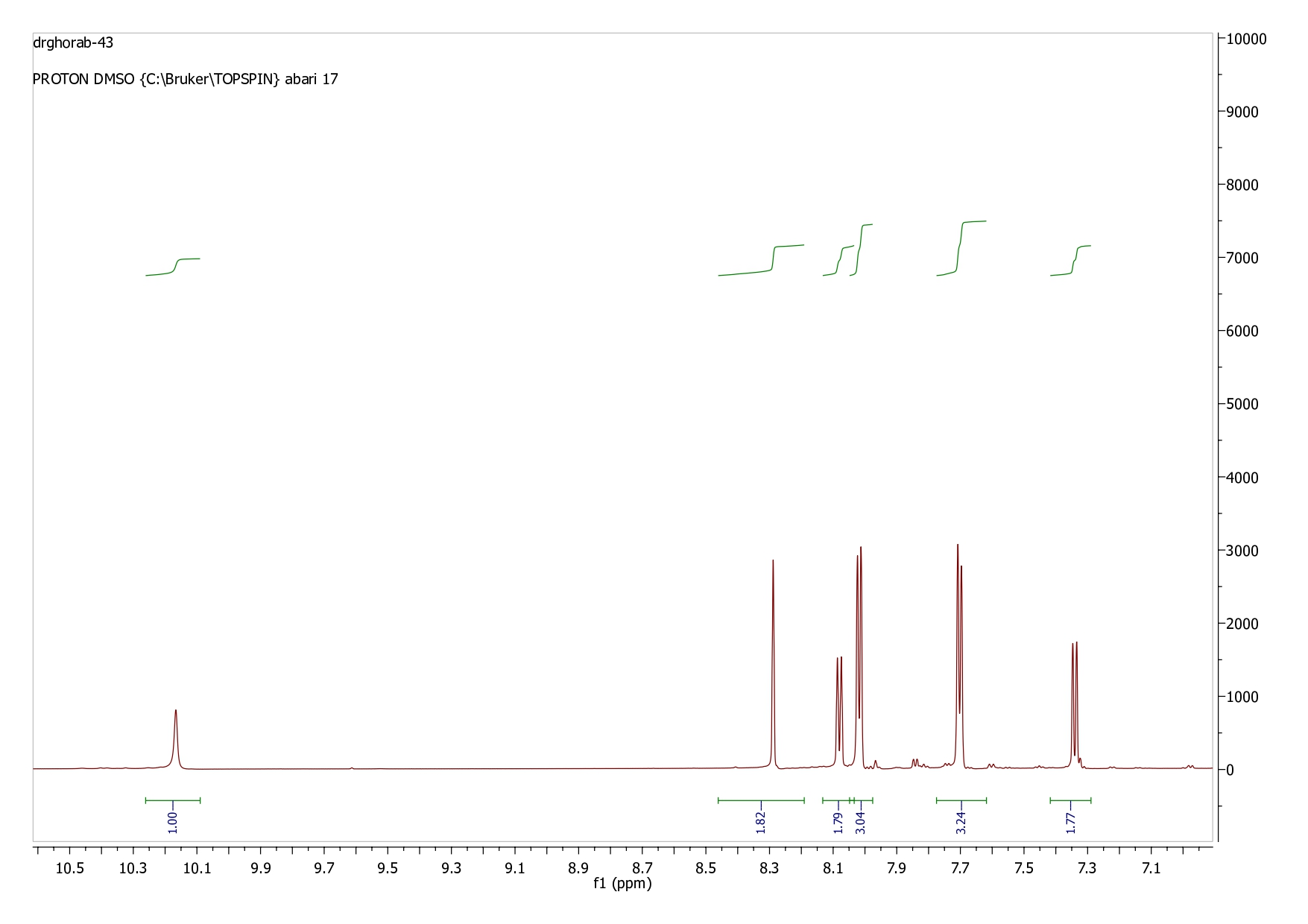


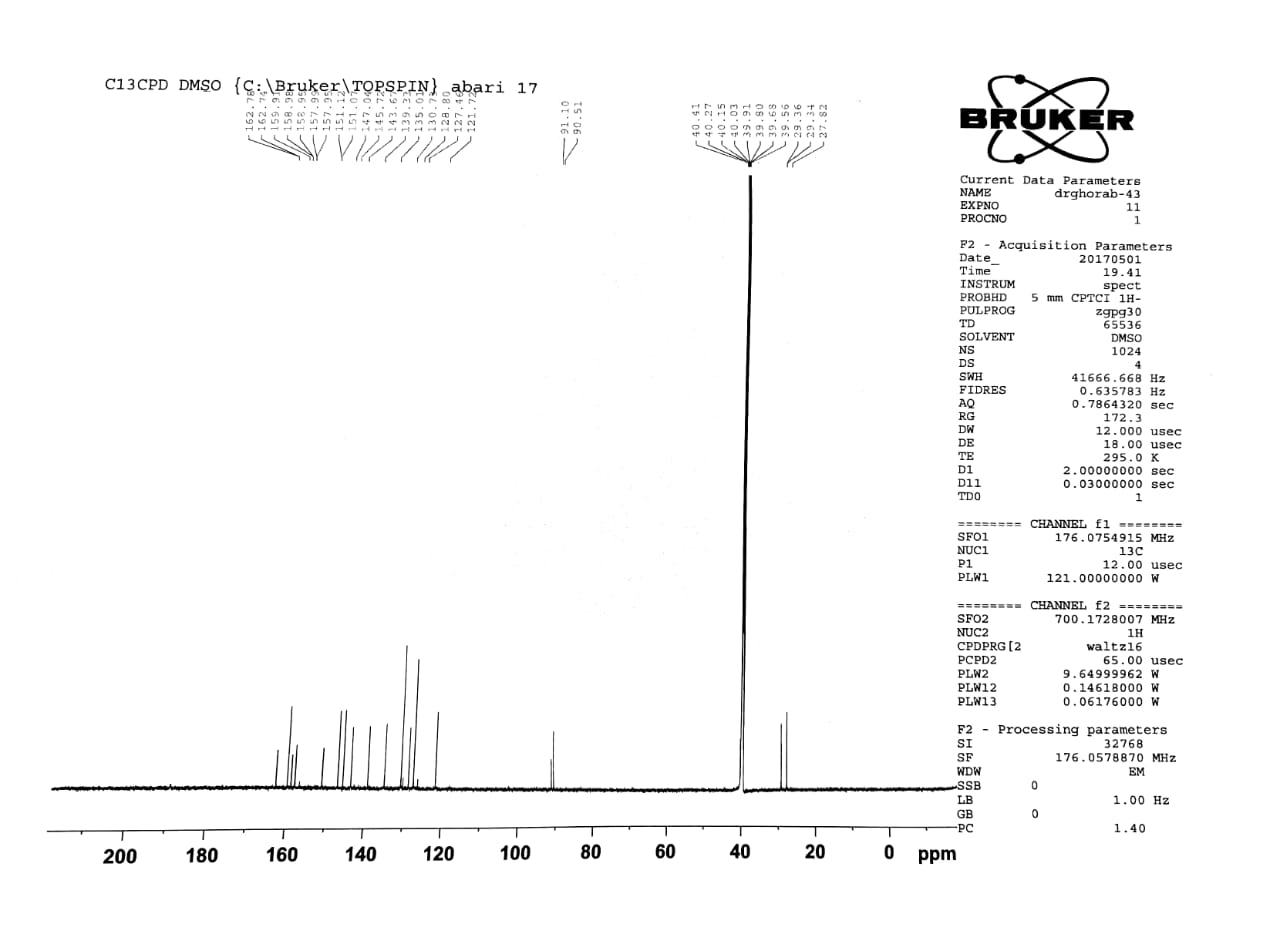


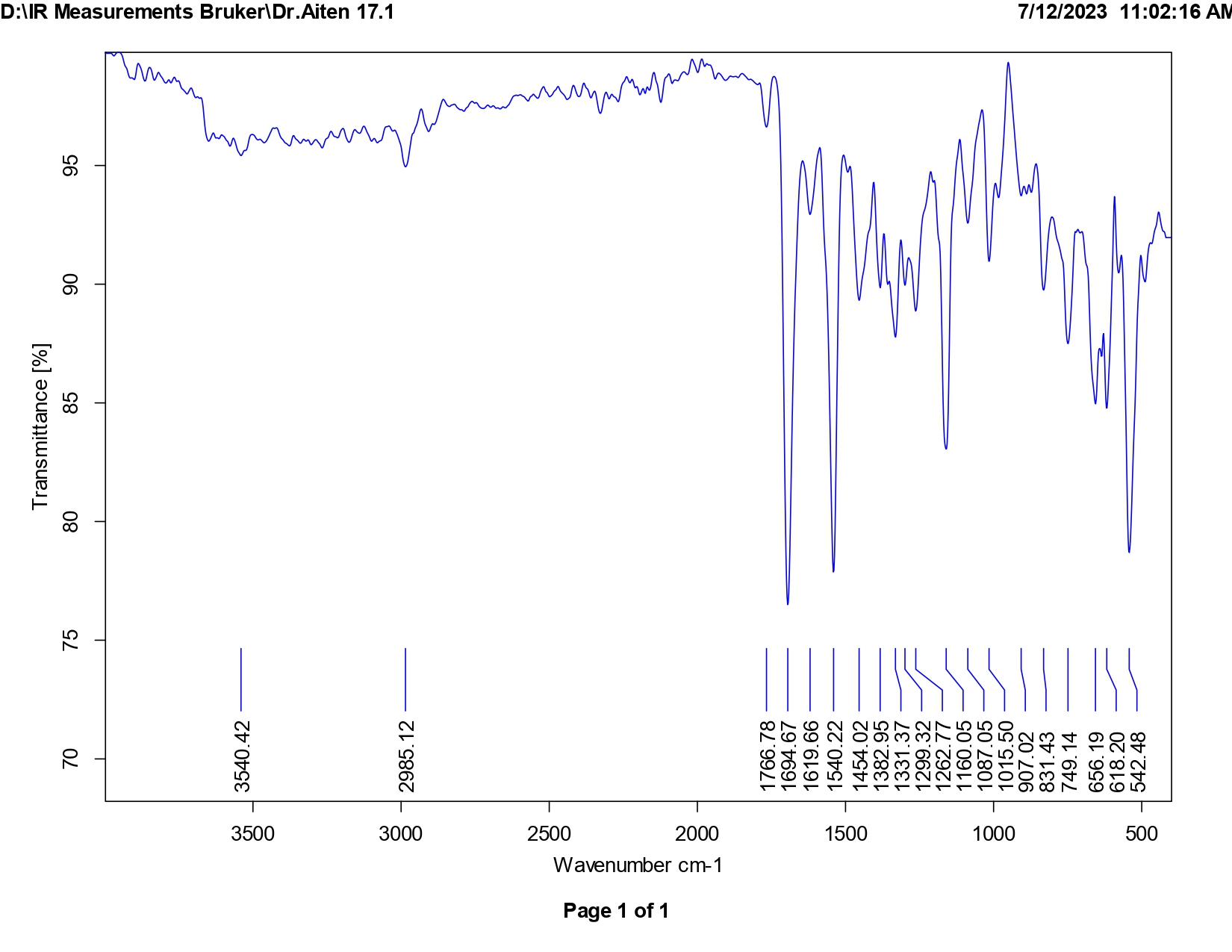


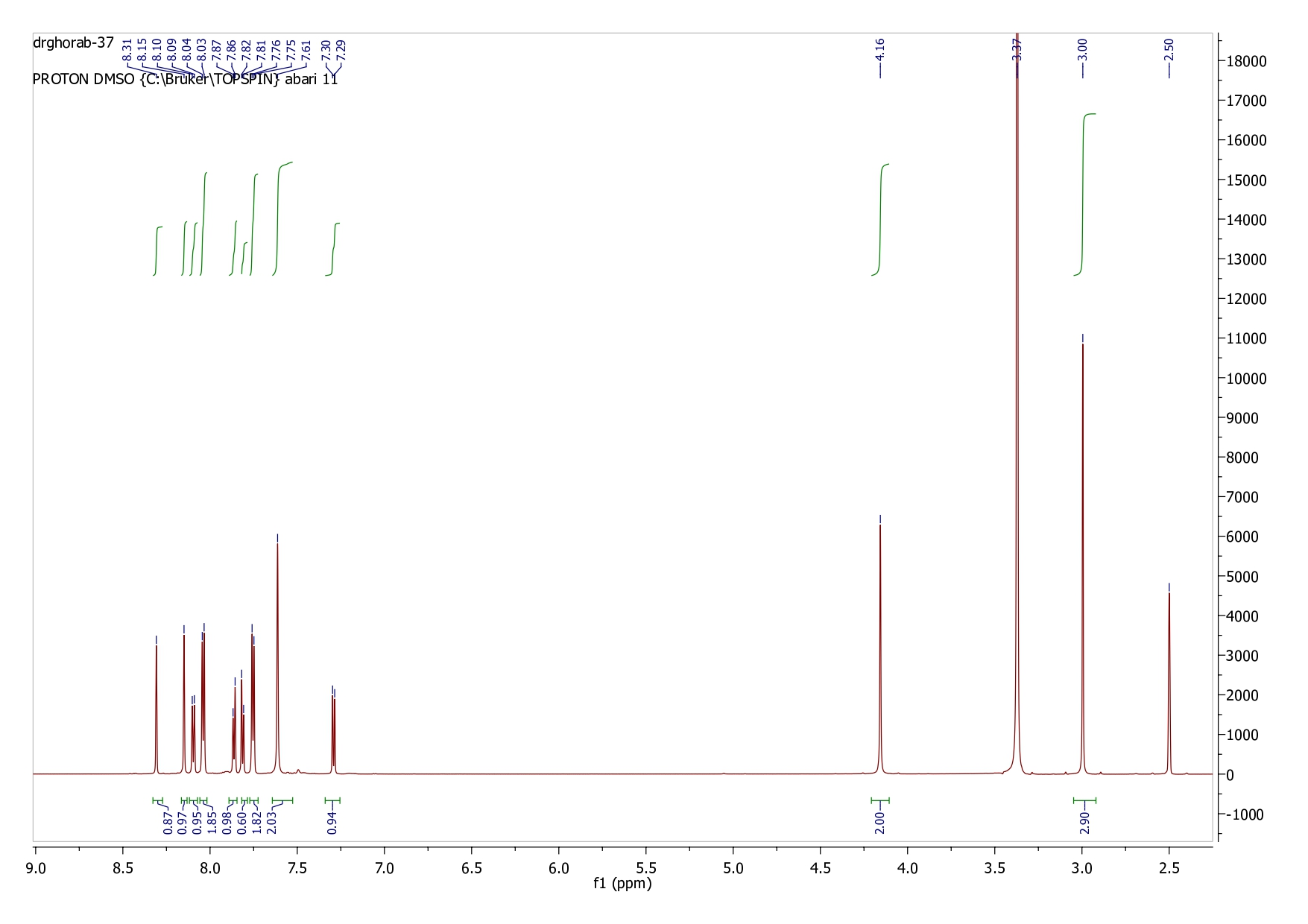


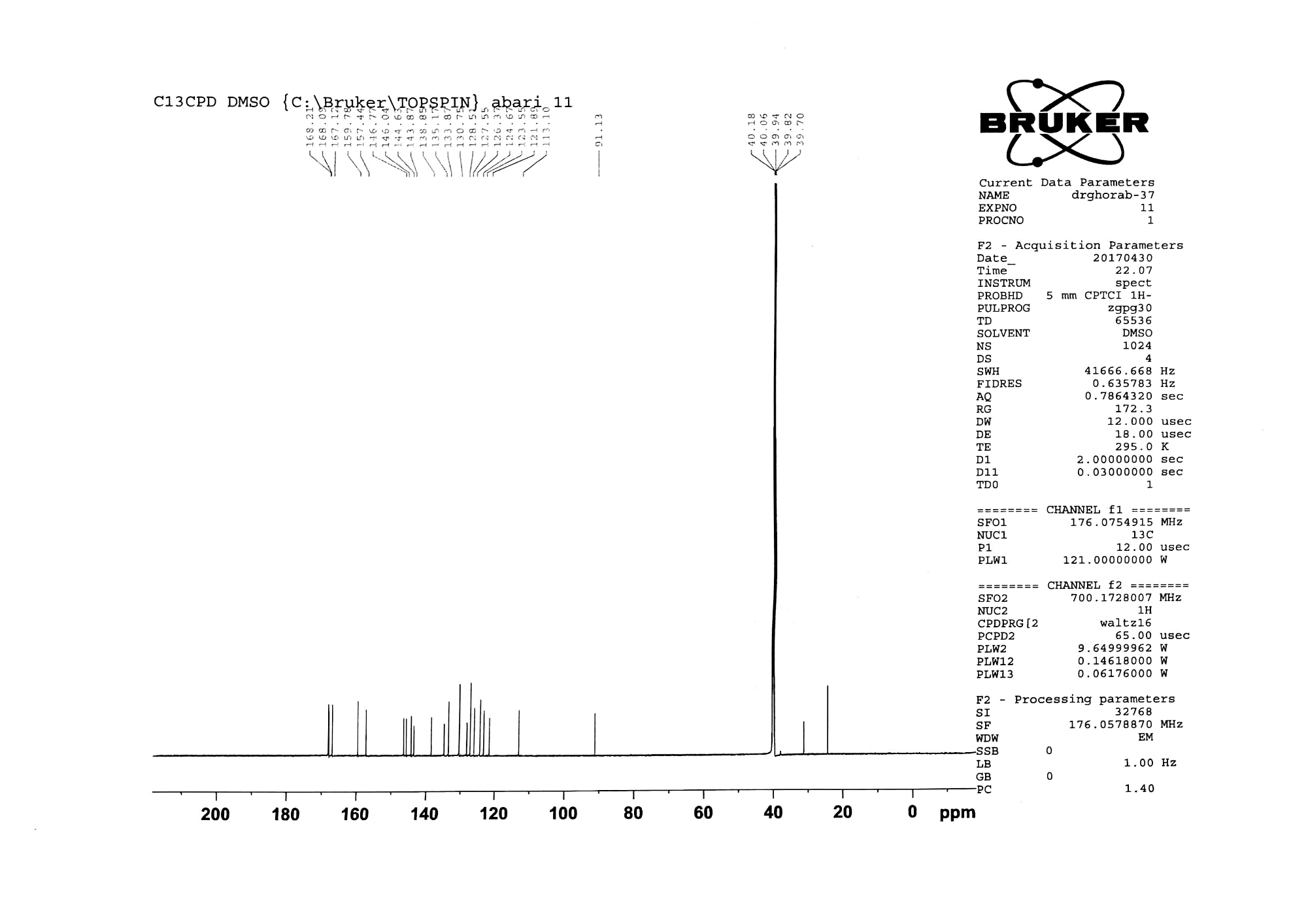

Supplement: Supplementary file 1 — Supplementary Information. [file 41598_2023_42239_MOESM1_ESM.docx]
